# Alternative splicing–triggered mRNA decay informs splice-switching targets for neurodevelopmental disorders

**DOI:** 10.1172/JCI197271

**Published:** 2026-02-12

**Authors:** Kaining Hu, Runwei Yang, Jiaming Qiu, Xinran Feng, Kayleigh J. LaPre, Jessica Tanouye, Yalan Yang, Xiaochang Zhang

**Affiliations:** Department of Human Genetics, The Neuroscience Institute, University of Chicago, Chicago, Illinois, USA

**Keywords:** Development, Neuroscience, Epilepsy, Neurodevelopment, RNA processing

## Abstract

Alternative splicing–triggered nonsense-mediated mRNA decay (AS-NMD) critically regulates gene expression, but the extent to which neuronal genes are regulated by AS-NMD remains understudied. Here, we identified over 3,000 developmentally regulated AS-NMD exons in mouse and human brains and validated them in cultured neurons. AS-NMD suppresses synaptic genes during brain development and differentially regulates more than 200 causal genes for neurodevelopmental disorders (NDDs). We detected a poison exon in *GRIA2* and identified splice-switching antisense oligonucleotides that suppressed *GRIA2* NMD and increased its functional isoforms. In summary, this study uncovers genes repressed by AS-NMD in the brain and nominates amenable splice-switching targets for treating dominant NDDs such as autism spectrum disorders and developmental epileptic encephalopathy.

## Abstract 2

This study reports alternatively spliced exons in the brain that trigger mRNA decay. Modulating such exons in disease-causal genes can potentially treat neurodevelopmental disorders.

## Graphical abstract

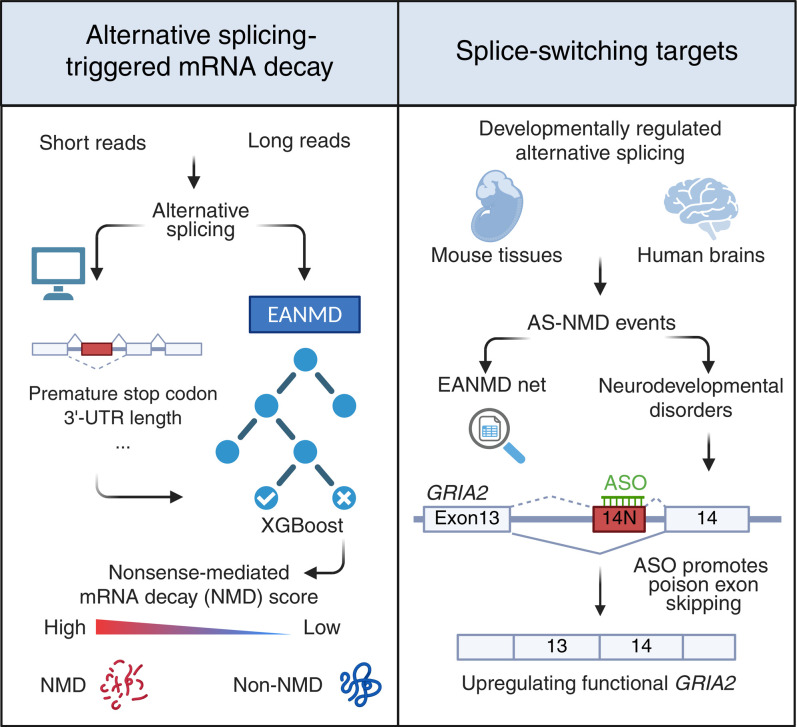

## Introduction

Alternative splicing (AS) enables the production of multiple mRNA isoforms from a single gene, thus playing an important role in gene regulation (1–3). AS happens between different species, tissues, and cell types (4–9), and it is modulated by *cis*-regulatory sequences and their associated RNA-binding proteins (RBPs) (10–13). AS has been reported as a primary source of phenotypic variation (14), and dysregulation of AS causes neurodevelopmental and neurodegenerative diseases (15).

Premature translational termination codons located approximately 50 nt or further upstream of splice junctions can trigger nonsense-mediated mRNA decay (NMD) in eukaryotes, mitigating the production of potentially harmful transcripts and proteins (16). In the canonical exon junction complex model, NMD is triggered during the pioneering round of translation and mediated by Upf1/2 and their associated proteins (17, 18). NMD has been reported to play a pervasive role in gene regulation (19), and blocking NMD in *Upf2*-knockout mice impairs cortical development (20). AS-triggered NMD (AS-NMD) regulates homeostatic expression of splicing regulators such as the SR proteins (21, 22) and cross-regulation between splicing regulators (23, 24). AS-NMD has been reported to regulate genes, especially chromatin regulators, in the brain (25).

Multiple human and mouse genetic studies have shown that dysregulation of individual AS-NMD exons can cause neurodevelopmental deficits. Dominant *SNRPB* mutations promoting *SNRPB* AS-NMD exon inclusion have been reported to cause cerebrocostomandibular syndrome (26). Dysregulation of cell-type–specific AS-NMD in *FLNA* has been reported to cause structural brain malformation (27). Abnormal AS-NMD exon inclusion in *SCN1A* causes epilepsy in humans (28). Deletion of the *Bak1* AS-NMD exon induces abnormal neuron loss and lethality in mice (29). Furthermore, dysregulation of RBPs such as Rbfox1 and TDP-43 triggers abnormal AS-NMD in neuronal genes and causes neurodevelopmental and neurodegenerative disorders (30–32). These observations suggest critical functions of AS-NMD in the brain.

Since the success of splice-switching oligonucleotides in treating spinal muscular atrophy (33, 34), AS-NMD exons have been increasingly recognized as promising therapeutic targets (35, 36). Splice-switching oligonucleotides have been developed to suppress AS-NMD and target *SCN1A* haploinsufficiency, which is currently under clinical trial to treat Dravet syndrome (37, 38). *SYNGAP1* haploinsufficiency is associated with a number of neurodevelopmental symptoms (39). Recent studies from us and others suggest that converting *SYNGAP1* AS-NMD isoform to the functional splice isoform can alleviate haploinsufficient phenotypes in mice and human cells (37, 40, 41). Thus, naturally occurring AS-NMD exons are potential switches for up- or downregulation of gene expression.

Despite the importance of AS-NMD exons in gene regulation and their potential in therapeutics, current reference gene annotations do not specify whether and which alternative exon triggers NMD. Importantly, there are few bioinformatics tools to predict the NMD potential of AS events (42, 43), and existing tools do not comprehensively consider noncanonical features of NMD (44–47). To fill this gap, we developed EANMD (Exon Annotation for NMD) and systematically annotated AS-NMD in developing mouse brains. We found that neuronal genes were suppressed by AS-NMD during early brain development. Sequence feature analysis for skipped exon–NMD (SE-NMD) events uncovered RBP motifs and targets of PTBP1/2. We further annotated AS-NMD exons in developing and adult human brains, validated them in human induced pluripotent stem cell-derived (iPSC-derived) neurons, and nominated target exons for splicing modulation, such as the AS-NMD exon in *GRIA2*. Collectively, this study identifies AS-NMD events that critically regulate gene expression and nominates amenable targets for therapeutic intervention.

## Results

### Identification of AS-NMD events.

**Figure 1:**
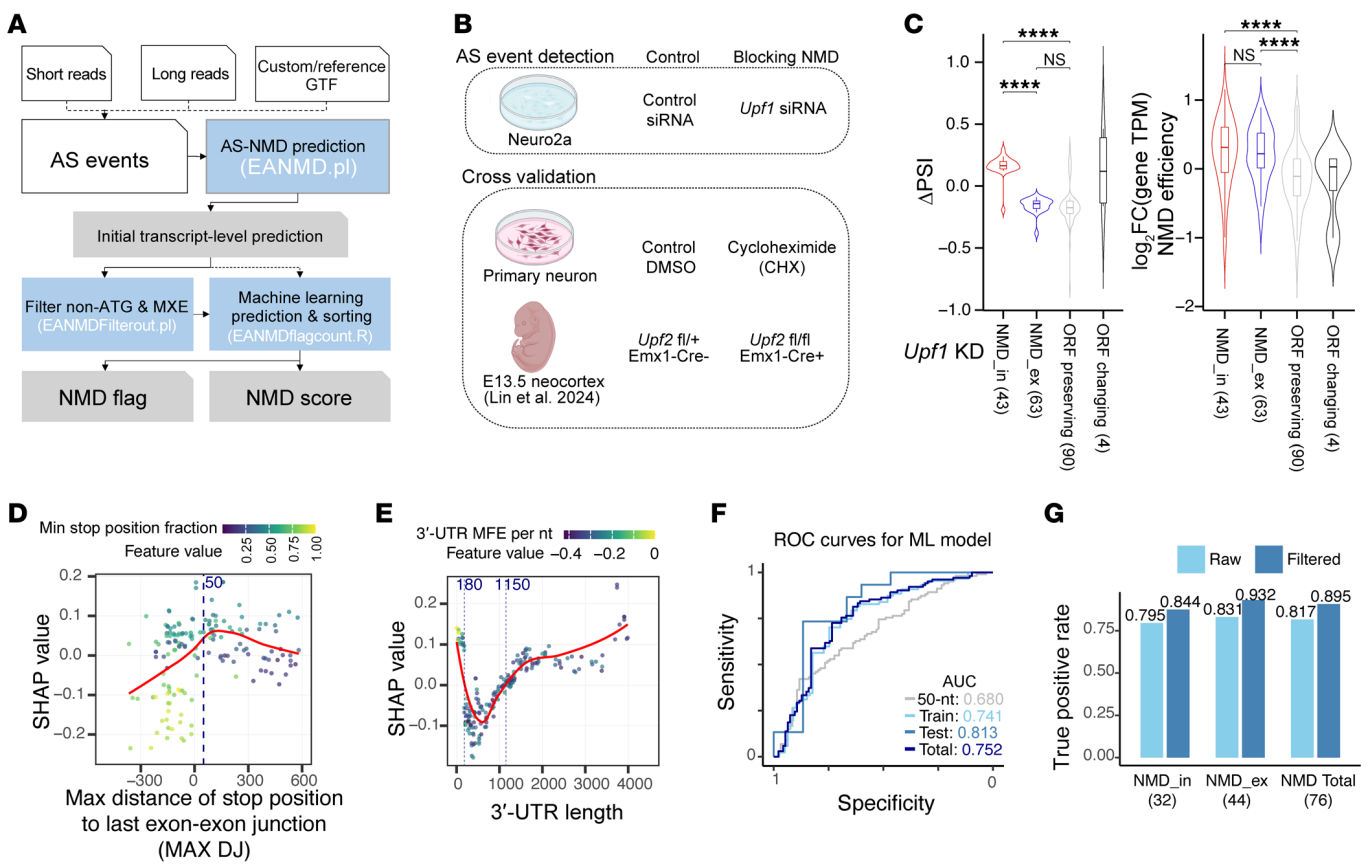
Identifying AS-NMD exons with EANMD. (**A**) An outline of the EANMD pipeline. AS events are identified from long- or short-read data, followed by transcript-level NMD prediction. The XGBoost machine learning model is applied to classify NMD flags and assign NMD scores. (**B**) Experimental datasets used for AS-NMD validation, including (a) *Neuro2a* cells with *Upf1* KD (*n* = 2 biological replicates), (b) primary mouse neurons treated with CHX (*n* = 2), and (c) *Upf2* conditional knockout in embryonic mouse neocortex (*n* = 2) (Lin et al., ref. 20). (**C**) Violin plots showing ΔPSI (left) and log_2_FC in gene expression (right) for NMD versus non-NMD SE events upon *Upf1* KD. Events triggering NMD exhibit increased transcript abundance after NMD inhibition, serving as a proxy for NMD efficiency. NMD_in (*n* = 43), NMD_ex (*n* = 63), ORF preserving (*n* = 90), and ORF changing (*n* = 4) events (1-way ANOVA followed by Tukey’s multiple-comparison test). (**D**) SHAP values of the minimum stop codon position fraction (Min stop Pos F) and the maximum stop codon distance to the last exon-exon junction (Max DJ), highlighting their interaction effect on NMD efficiency. (**E**) SHAP analysis of 3′-UTR length. NMD efficiency increased when 3′ UTRs were either longer than 1,150 nt or shorter than 180 nt. MFE, minimum free energy. (**F**) Receiver operating characteristic (ROC) curves for NMD classification. Using the 50 nt rule, the ROC-AUC is 0.680 (gray line). Incorporating the machine learning–predicted NMD score improves classification performance (AUC = 0.752, total SE events, navy blue line). ML, machine learning. (**G**) Filtering events based on NMD scores enhances AS-NMD detection.

EANMD identifies NMD exons based on the 50 nt rule and additional transcript-level features (Figure 1A and Supplemental Figure 1A; supplemental material available online with this article; https://doi.org/10.1172/JCI197271DS1) (48–50). Given the coordinates of an alternative exon and its flanking exons, EANMD retrieves all relevant transcripts, simulates transcript isoforms with or without the alternative exon, and computes the distance from the translational stop codon to the last exon-exon junction (distance junction [DJ]). After filtering out transcripts with non-ATG start codons or mutually exclusive exons (MXEs; see Methods and Discussion), AS events are classified as NMD_in (when the exon is included, poison exon) or NMD_ex (when excluded) when the DJ is greater than 50 nt or as customized. Non-NMD exons are also flagged based on their impact on the ORF and position in the untranslated regions (Supplemental Figure 1B). EANMD reconstructs isoforms in addition to reference transcripts, enabling the identification of previously unannotated AS-NMD events.

We evaluated a group of NMD-prone exons (25, 51), and EANMD correctly annotated all of them (Supplemental Table 1a). We further evaluated EANMD by blocking NMD with *Upf1* knockdown (KD) in Neuro2a cells (Figure 1B). *Upf1* KD significantly changed the expression for 732 genes, among which *Upf1* was the most significantly downregulated gene (Supplemental Figure 1C, |log_2_ fold change [log_2_FC]| > 1, adjusted *P* < 0.001). Among the 275 differentially spliced SEs upon *Upf1* KD (Supplemental Figure 1A; |Δ percent spliced in [ΔPSI]| > 0.1, FDR < 0.05), EANMD flagged 258 events (93.8%), including 44 NMD_in and 65 NMD_ex exons (Figure 1C, Supplemental Figure 1D, and Supplemental Table 1b). Transcripts for 52 out of the 109 EANMD-predicted AS-NMD events were annotated as NMD targets in the GENCODE M25 reference (Supplemental Table 1b). In addition to *Upf1* KD, we used 2 additional datasets to evaluate the accuracy of EANMD prediction (Figure 1B): (a) primary neurons treated with cycloheximide (CHX), a translation inhibitor (Supplemental Figure 1E; this study), and (b) E13.5 *Upf2*-knockout mouse brains (Supplemental Figure 1F; published dataset) (20). Across all 3 conditions, the inclusion of NMD_in exons was increased when NMD was blocked, while the inclusion of NMD_ex exons was decreased; the expression levels for both NMD_in and NMD_ex genes, but not non-NMD genes, were upregulated upon NMD inhibition (Figure 1C and Supplemental Figure 1G). These observations collectively suggest that EANMD is robust in identifying NMD exons.

We developed a predictive model for NMD efficiency (mRNA level increase upon NMD inhibition) with splicing and transcript features (Supplemental Figure 1H and Supplemental Table 1c). The optimal machine learning model (XGBoost) explained approximately 80% of the variance. SHAP (Shapley additive explanation) analysis revealed that the most predictive feature was the interaction between the relative position of the stop codon in the transcript and the maximal distance between the stop codon and the last splice junction (Max DJ). The AS events were less likely to trigger NMD if the stop codons were closer to the 5′ end of the transcripts (Figure 1D). When the stop codons were located in the last exons (Max DJ < 0), longer 3′-UTRs (>1,150 nt) enhanced the probability of mRNA decay (Figure 1E and Supplemental Figure 1I). Additional features also contributed to NMD efficiency, such as secondary structures in the 3′-UTR and the length of SEs. We determined the optimal cutoff based on NMD scores (the model-predicted NMD efficiency value) and improved NMD prediction (Figure 1, F and G).

We compared EANMD to 2 existing tools, SpliceTools-SETranslateNMD (42) and NMD Classifier (43) (based on Ensembl/NCBI annotations; Supplemental Table 1b), using the *Upf1*-KD dataset. The NMD Classifier annotated 18.2% of the 275 input events, in contrast to SpliceTools (85.8%) and EANMD (93.8%). While 23 NMD_in and 22 NMD_ex events were correctly predicted by all 3 tools (Supplemental Figure 2A), EANMD identified 2 NMD_in events missed by other methods (Supplemental Figure 2B). SpliceTools had 18 false positives, of which 12 were in-frame and 3 had DJ < 50 nt. EANMD showed the best precision-recall balance (F1 = 0.952 using the 50 nt rule; Supplemental Figure 2C and Supplemental Figure 3). In addition, EANMD identified 32 alternative 5′-splice site, 28 alternative 3′-splice site, and 87 intron retention events that could trigger NMD (Supplemental Figure 1A and Supplemental Table 1, d–f). Collectively, these results indicate that EANMD reliably identifies AS-NMD events.

### Brain-specific and developmentally regulated AS-NMD.

**Figure 2:**
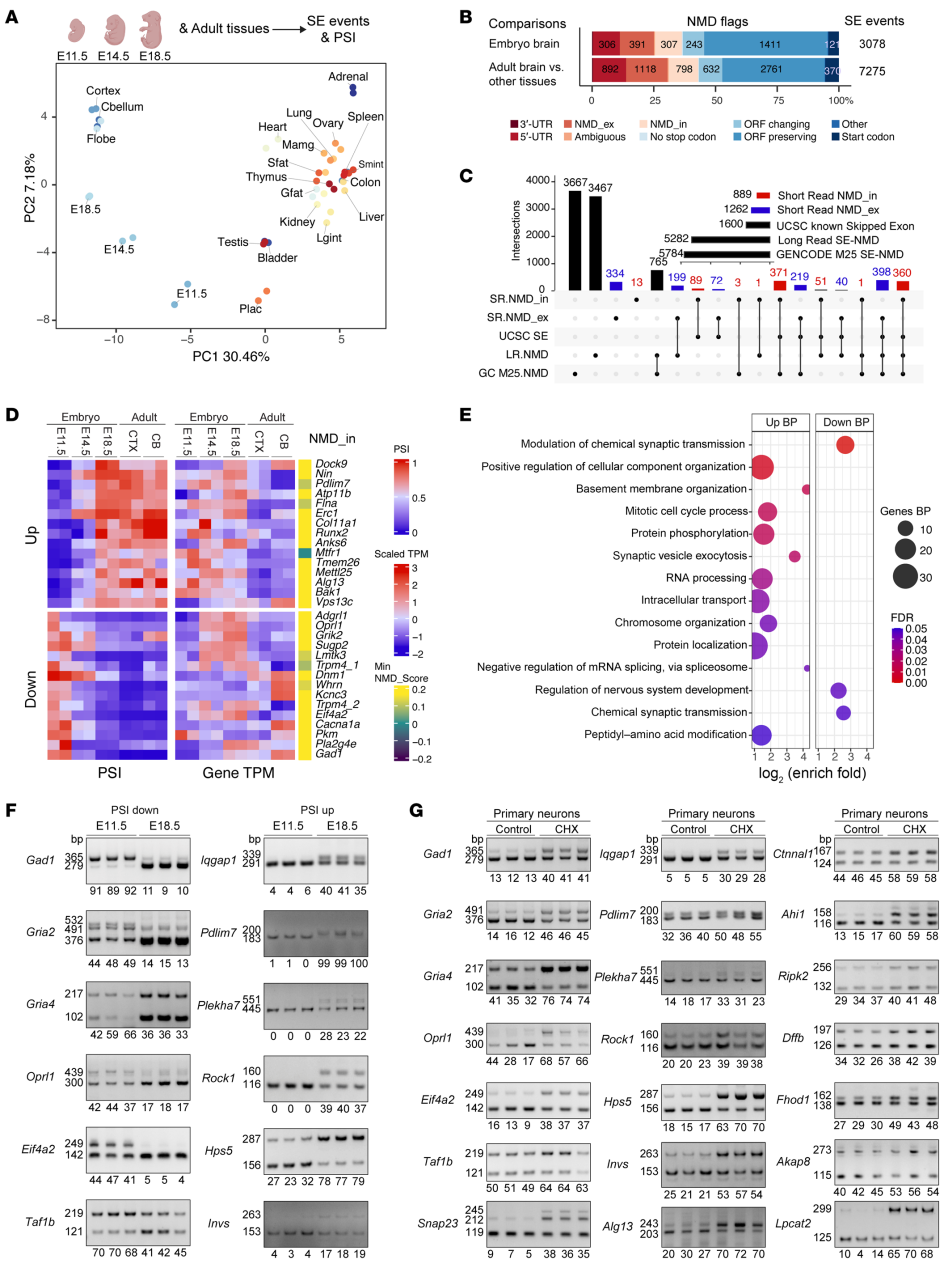
Dynamic AS-NMD in the developing mouse brain. (**A**) PCA of PSI values from dynamic SE events (0.03 < minimum PSI of all samples < 0.95, *n* = 22,464) across the developing and adult mouse brains and other adult tissues (2 biological replicates for each group). (**B**) Predicted AS-NMD events that were differentially spliced across different embryonic stages or between the adult mouse brain and other adult tissues. (**C**) UpSet plot summarizing SE-NMD events in developing and adult mouse brains, as well as long read–supported SE-NMD events (E14.5/E18.5), GENCODE M25, and UCSC cassette exons. (**D**) Heatmap of the top 15 developmentally upregulated (from E11.5 to E18.5 brains) and top 15 downregulated NMD_in SE exons showing their PSI (splicing, left) and TPM (gene expression, right) changes during mouse brain development. CTX, cortex; CB, cerebellum. (**E**) Gene Ontology and biological process (BP) term enrichment of genes carrying NMD_in exons. (**F**) RT-PCR validation and quantification of NMD_in events in mouse E11.5 and E18.5 dorsal forebrains. Numbers indicate PSI values. For all shown events, 2-tailed *t* test *P* < 0.05, *n* = 3 biological replicates per group. (**G**) RT-PCR validation and quantification of NMD_in events in mouse primary neurons treated with CHX. Numbers indicate PSI values. For events, 2-tailed *t* test *P* < 0.05, *n* = 3 biological replicates per group.

We investigated dynamic AS-NMD events during mouse brain development at the beginning (E11.5), peak (E14.5), and end of neurogenesis (E18.5), together with 20 adult tissues such as the cortex, cerebellum, and non-brain tissues (2 biological replicates for each tissue/stage) (52) (Figure 2A). We identified 22,464 SE events across all samples using rMATS (0.03 <minimum PSI < 0.95 for each sample). Principal component analysis (PCA) of the SE PSI values demarcated different tissues (Figure 2A). The brain samples stood out on PC1, and brain tissues from different developmental time points were further segregated on PC2, suggesting brain-specific and developmentally regulated splicing events (Figure 2A and Supplemental Figure 4A).

We performed pairwise comparisons and identified differential SE events (|ΔPSI| > 0.1, FDR < 0.05). MXEs were frequently annotated as SEs and were filtered out by imbalanced upstream and downstream junction coverages (see Methods and Discussion). Overall, 3,078 SE events exhibited significant dynamic changes in embryonic brains (E11.5, E14.5, and E18.5), among which EANMD identified 307 (10.0%) NMD_in and 391 (12.7%) NMD_ex events (Figure 2B). Among the 7,275 differential SEs between adult brain (cortex, frontal lobe, and cerebellum) and non-brain tissues, EANMD predicted 798 (11.0%) NMD_in and 1,118 (15.4%) NMD_ex events. Altogether, 889 NMD_in and 1,262 NMD_ex events were predicted out of 8,144 SEs (Figure 2B). We further analyzed PacBio long-read sequencing from the E14.5 and E18.5 mouse brains and found that 14.4% (8,636/59,680) and 10.4% (4,790/45,868) of coding sequence–containing isoforms, respectively, were predicted to be NMD targets. We found that more than half (50.4%, 637/1,262) of the EANMD-predicted NMD_ex exons from short reads were also supported by long reads (Figure 2C and Supplemental Table 2a). Compared with previously reported AS-NMD events (25, 53–56), we nominate 1,360 additional SE-NMD events in the mouse brain (Supplemental Table 2a).

We filtered the AS-NMD events using predicted NMD scores (Supplemental Figure 4, B and C) and found a negative correlation between NMD_in exon inclusion and gene expression levels between E11.5 and E14.5; in contrast, NMD_ex events exhibited a positive correlation (Supplemental Figure 4D). Comparisons between E14.5 and E18.5 brain samples showed the same trend (Supplemental Figure 4E). We classified the splicing events into 12 clusters for downstream analysis (see Methods). The majority of NMD_in (67%) and non-NMD events (ORF preserving, 68%; ORF changing, 63%), but not NMD_ex (35%), showed increasing PSI values during brain development (Supplemental Figure 4F). In *Upf1*-KD, CHX-treated, and *Upf2* conditional knockout samples, we observed a clear trend of increased PSI values for embryonic NMD_in events and decreased PSI values for NMD_ex events (Supplemental Figure 4G). These results suggest that the predicted NMD events were subjected to mRNA decay.

Dynamic NMD events spanning E11.5 to E18.5 illustrated that inclusion of NMD_in exons in neuronal genes (Supplemental Figure 5A) such as *Gad1* were downregulated (Figure 2D), and the inclusion of NMD_ex exons in neuronal genes such as *Scn3a* were upregulated during brain development (Supplemental Figure 5B). Gene Ontology enrichment analysis showed that developmentally downregulated (Down) NMD_in exons were associated with chemical synaptic transmission and developmentally upregulated (Up) NMD_in exons were associated with terms such as basement membrane and mitotic cell cycle (Figure 2E and Supplemental Table 2, b–e). By contrast, NMD_ex (Up) events were enriched in chemical synaptic transmission (Supplemental Figure 5C). RT-PCR experiments validated dynamic AS-NMD events between E11.5 and E18.5 (Figure 2F), which anticorrelated with mRNA levels of their host genes (Supplemental Figure 5, D and E). We treated primary cortical neurons with CHX and found the predicted NMD_in exons were upregulated (Figure 2G and Supplemental Figure 5, F–H) while the NMD_ex events were downregulated (Supplemental Figure 6, A and B). These results indicate that neuronal genes, especially genes encoding synaptic proteins, are suppressed by AS-NMD during brain development (Supplemental Figure 6C and Supplemental Table 2).

### Cis-regulatory sequences for AS-NMD exons.

**Figure 3:**
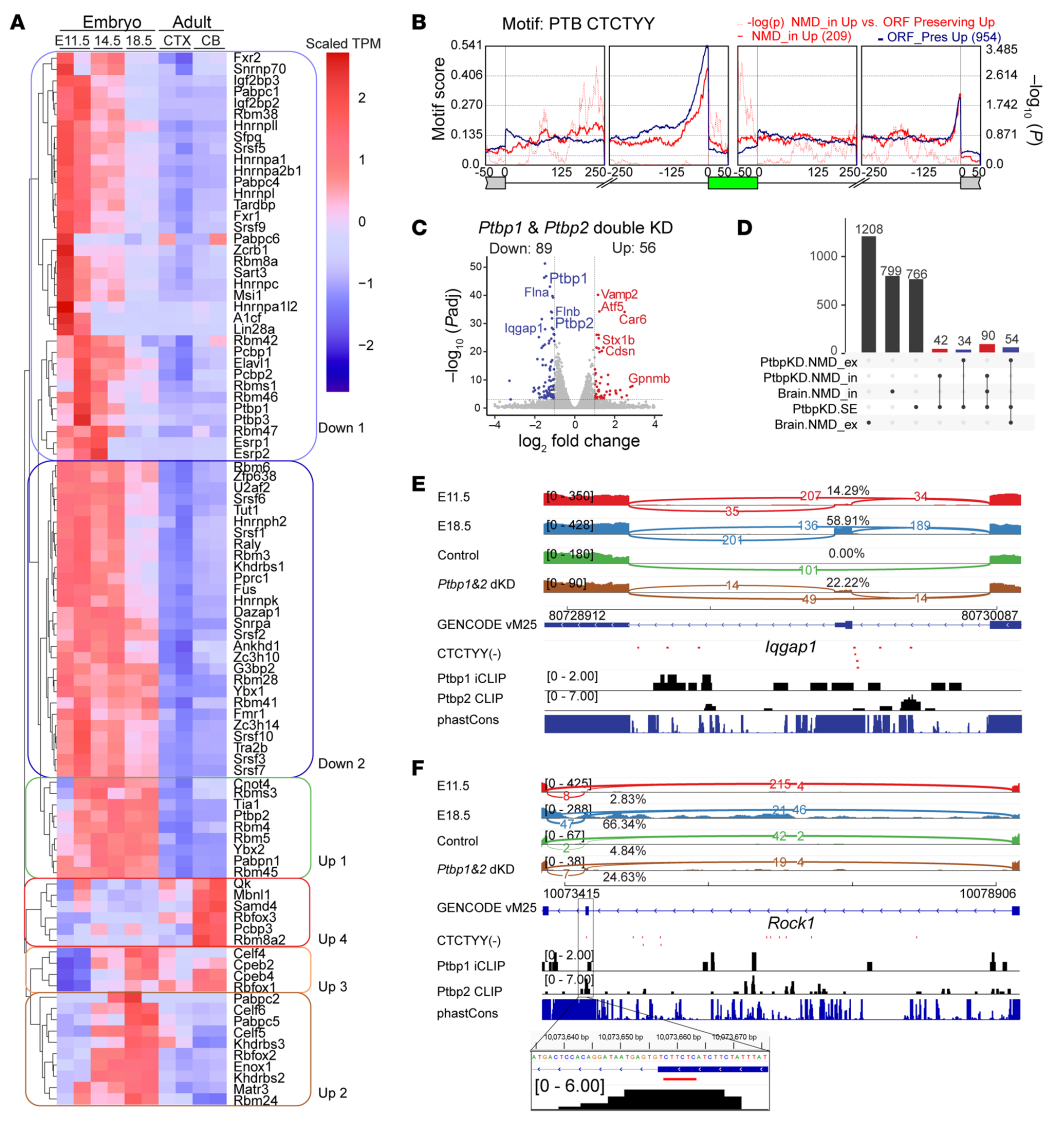
RBPs and sequence motifs associated with AS-NMD exons. (**A**) Heatmap showing expression levels of 93 RBPs, binding motifs of which were significantly enriched in flanking sequences of SE-NMD events (Supplemental Figure 8). RBPs were grouped into 6 clusters based on their expression patterns during brain development. (**B**) PTBP binding motifs were significantly enriched within the SEs compared with the ORF_preserving Up exons. Red, test group; blue, background; dashed line, –log_10_ (*P*) from Wilcoxon’s rank-sum test. (**C**) Volcano plot showing differentially expressed genes upon *Ptbp1/2* double KD in Neuro2a cells (*n* = 2 biological replicates for each condition, |log_2_FC| ≥ 1, adjusted *P* < 0.001). (**D**) UpSet plot illustrating the AS-NMD events that were differentially spliced in the developing mouse brain and significantly affected upon *Ptbp1/2* KD. (**E**) The AS-NMD_in exon in *Iqgap1* (chr7:80729643–80729691, mm10) showed higher inclusion in E18.5 and in sh*Ptbp1/2* cells. (**F**) The AS-NMD_ex *Rock1* exon 28 (chr18:10073656–10073700) showed higher inclusion in sh*Ptbp1/2* cells, Ptbp1/2 binding motifs, and Ptbp1/2 CLIP-seq tags.

To identify potential splicing regulatory sequences that may be under selection pressure, we compared the conservation score (UCSC PhastCons 60) of the SE-NMD exons and their flanking introns and exons (Supplemental Figure 7). The upstream and downstream introns of SE-NMD_in events showed higher conservation than those in non-NMD SE events (Supplemental Figure 7, D and E), suggesting the presence of regulatory elements. Next, we performed motif enrichment analyses by contrasting NMD and non-NMD SE events using rMAPS2 (57). By comparing NMD_in Up/Down, NMD_ex Up/Down, ORF_preserving Up/Down, and nonsignificantly changed SEs between E11.5 and E18.5, we discovered 114 significant RBP motifs (Supplemental Figure 8; *P* < 0.05). These motifs corresponded to 93 distinct RBPs, the majority of which exhibited dynamic gene expression during mouse brain development (Figure 3A) and were hierarchically clustered into 6 groups based on expression patterns. Notably, most RBPs were downregulated during embryonic brain development (Figure 3A). These observations suggest that different combinations of RBPs may fine-tune gene expression through AS-NMD.

In the comparison of NMD_in Up to ORF_preserving Up exons, the most significantly enriched RBP motif in the upstream intron was A[AG]AG[AG][AG][AG] for SRSF10 (58) (Supplemental Figure 8 and Supplemental Figure 9A). The most significantly enriched motif in the downstream intron across all comparisons was [AT]C[AT][AT]C for SRSF3 (58) (Supplemental Figure 9B). These observations suggest that SR proteins synergistically regulate AS-NMD exons during brain development. The comparison of NMD_in Up to Down events indicated significant enrichment of the CUCUYY motif for PTBPs (Supplemental Figure 8). NMD_in Up events displayed a higher density of the PTBP1 motif upstream of SEs (Supplemental Figure 9C). *PTBP1* expression decreased during brain development (Figure 3A) and may act as a suppressor of certain NMD_in Up exons. Comparison of NMD_in Up to ORF_preserving Up exons showed that the PTBP1 motifs were significantly enriched inside SEs (Figure 3B; *P* < 0.01), suggesting that PTBPs may directly bind to and repress the target NMD_in exons. We also found enrichment of other motifs, such as ACAG, in the upstream intron (Supplemental Figure 9, D–F), implying additional sequence features in regulating NMD exons.

### AS-NMD exons regulated by Ptbp1 and Ptbp2.

Given their enriched motifs, we performed *Ptbp1/2* shRNA KD and RNA-seq in Neuro2a cells to evaluate their functions in regulating AS-NMD (Figure 3C and Supplemental Figure 10A). Differential gene expression analysis confirmed that *Ptbp1* and *Ptbp2* were significantly decreased after KD (Figure 3C and Supplemental Figure 10, B and C). *Ptbp1/2* double KD caused more genes to decrease expression than to increase. Several known *Ptbp1/2* target AS-NMD genes such as *Flna* were recapitulated (Figure 3C). We identified 604 differential SEs in *Ptbp1* KD, 16 SEs in *Ptbp2* KD, and 727 SE events in *Ptbp1/2* double KD, totaling 986 SE events across all comparisons (Figure 3D). The Ptbp binding motifs were significantly enriched for differentially spliced exons (Supplemental Figure 10D). We reanalyzed the *Ptbp1/2* cross-linking immunoprecipitation sequencing (CLIP-seq) datasets (see Methods) and found that *Ptbp1/2* CLIP peaks were significantly enriched in the upstream regions of these 986 differential exons (Supplemental Figure 10E). Applying EANMD to the *Ptbp1/2* shRNA KD samples uncovered 132 NMD_in and 88 NMD_ex events (Figure 3D). We examined the *Ptbp1/2*-responsive AS-NMD events in the *Upf1* KD dataset and found 137 of them showed increased transcripts per million reads (TPM) and consistent PSI changes when NMD was blocked (Supplemental Figure 10F and Supplemental Table 3a; NMD_in, 76; NMD_ex, 61). Among these, there were 22 AS-NMD genes associated with the synapse (Supplemental Table 3b). Overall, we observed more NMD_in exons than NMD_ex in *Ptbp1/2* KD (Supplemental Figure 10G). These results suggest that Ptbp1/2 tends to promote NMD_in. We examined CLIP peaks within the 100 nt upstream or on the SE using the FIMO tool (59) and identified 57 NMD_in and 10 NMD_ex events that were associated with both Ptbp1/2 motifs and CLIP peaks (Supplemental Table 3a). Ptbp1/2-regulated NMD_in events were illustrated in *Iqgap1* (Figure 3E) and *Rock1* (Figure 3F), as well as the NMD_ex exon in *Gabbr1* (Supplemental Figure 10H). In summary, we identified dozens of AS-NMD events that were directly regulated by Ptbp1/2 proteins.

### Identification of AS-NMD exons in developing and adult human brains.

**Figure 4:**
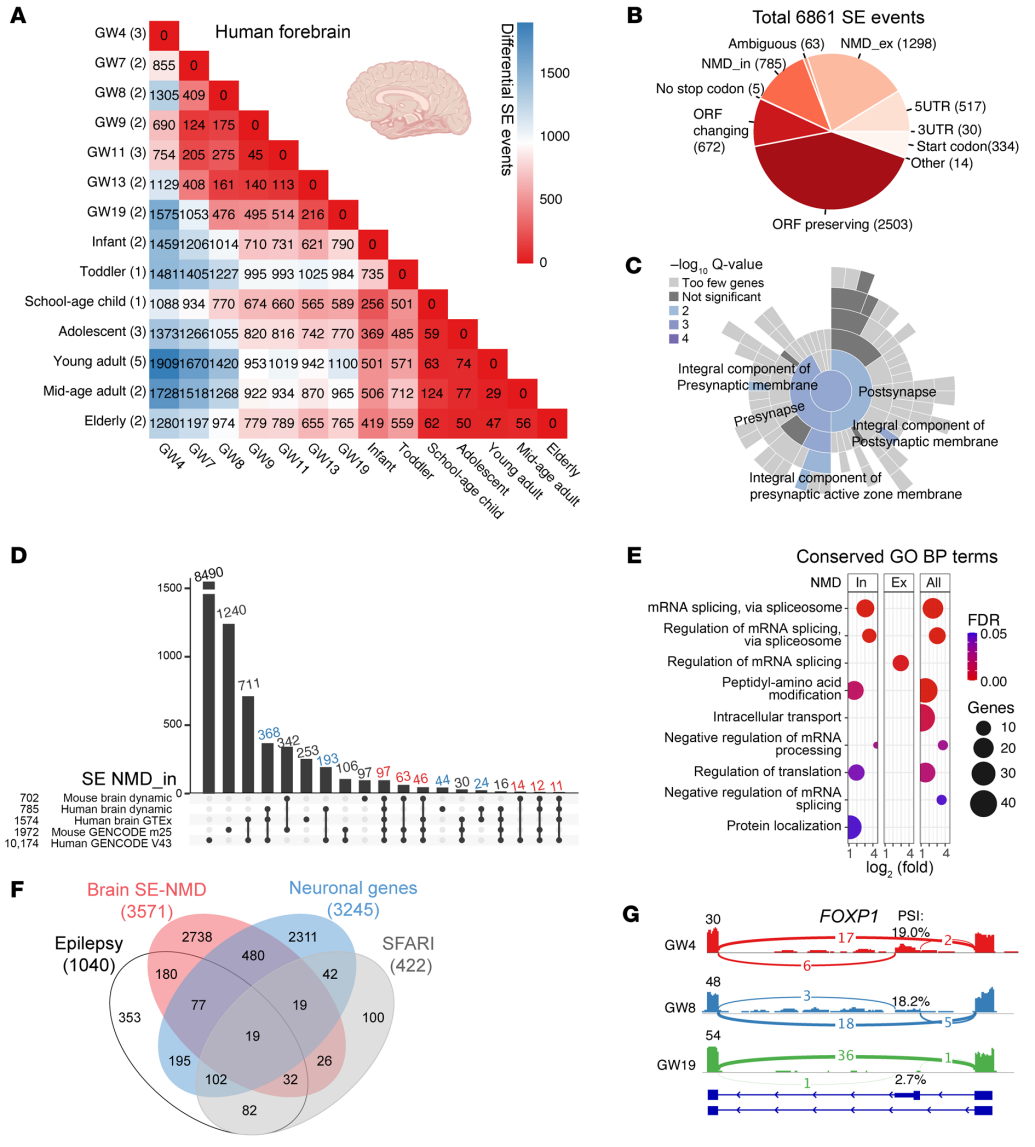
Developmentally regulated AS-NMD in the human brain. (**A**) Heatmap showing the number of differentially spliced SEs across 14 developmental time points in the human forebrain (numbers of samples are indicated in parentheses; see Methods). (**B**) A pie chart showing over 2,000 dynamic AS-NMD events detected in the human brain. (**C**) SynGO enrichment of NMD suppressed genes (ΔPSI < –0.1) in the GW11–13 group compared with the infant-toddler group. Developmentally downregulated NMD_in and upregulated NMD_ex targets were enriched in synapse, especially the presynaptic and postsynaptic membranes. (**D**) UpSet plot showing conserved SE-NMD_in events between human and mouse datasets. (**E**) Enriched biological processes for human-mouse conserved SE-NMD genes. GO, Gene Ontology; BP, biological process. (**F**) Venn diagram showing the intersection of human and mouse brain SE-NMD–targeted genes with neuronal genes (GO), Gene4Epilepsy-annotated genes, and autism spectrum disorder genes (SFARI). (**G**) Sashimi plot showing a representative AS-NMD_in exon in *FOXP1* (GRCh38, chr3:70972010–70972180).

We applied EANMD to 32 human forebrain samples (European Nucleotide Archive, PRJEB26969) across 14 time points (gestation week 4 [GW4] to GW19, infant, toddler, school-age child, adolescent, young adult, middle-aged adult, and elderly). In total, 88,976 SEs were detected, and PCA of the PSI values suggested developmentally regulated splicing profiles in the human brain (Supplemental Figure 11A). After filtering out MXE events, we identified 6,861 dynamic SEs (Figure 4A; |ΔPSI| > 0.1, FDR < 0.01). EANMD flagged 785 NMD_in and 1,298 NMD_ex events in the dynamic SEs (Figure 4B, Supplemental Figure 11B, and Supplemental Table 4a). We identified 1,737 NMD-sensitive events based on NMD scores (cutoff: 0.131; Supplemental Figure 11, B and C). Comparing GW11–13 with infant-toddler groups, developmentally downregulated SE-NMD host genes (NMD_in ΔPSI < –0.1 or NMD_ex ΔPSI > 0.1) were enriched in the synapse (48 out of 333 genes, *P* < 0.001), particularly integral components of pre- or postsynaptic membranes (Figure 4C), such as *GRIA1*, *GRIA2*, *GRIA3*, *GRIK2*, *KCNC3*, *KCNC4*, *KCNN2*, *PTPRS*, *TRPC1*, *ABCC8*, and *CACNA1A*. The SynGO enrichment analysis suggested that synaptic genes were suppressed by AS-NMD in early brain development (Figure 4C).

We analyzed human brain samples in the GTEx v7 dataset (60), and EANMD identified 183 NMD transcripts that were annotated as protein-coding transcripts in GENCODE 38; conversely, 21 transcripts classified as NMD by GENCODE were predicted to be protein-coding transcripts by EANMD (Supplemental Figure 11D and Supplemental Table 5). Manual inspection showed that the majority (18/21) failed to meet the 50 nt rule, with the DJ ranging from 17 to 46 nt (33 ± 10). We annotated brain region–specific SEs and identified 1,574 NMD_in and 1,462 NMD_ex events (Supplemental Figure 11D and Supplemental Table 5a). We further investigated AS events detected by SUPPA2 from GENCODE v43 annotation and identified 10,277 SE-NMD_in and 6,479 SE-NMD_ex events (Supplemental Table 6). In total, we identified 1,653 SE-NMD targeted genes that had not been annotated as NMD targets in the GENCODE reference (Supplemental Figure 11E).

We lifted the mouse brain SE event coordinates (mm10) to the human genome hg38 (7,280, 89.4%) and identified conserved human SEs for 3,295 (45.3%) of the lifted mouse SE events (Supplemental Figure 11F). Notably, the majority of lifted SEs retained their AS-NMD annotation across species: 130 out of 151 NMD_in and 178 out of 191 NMD_ex SEs in humans were consistent with annotations in mice (Figure 4D, Supplemental Figure 11G, and Supplemental Table 4a). These human-mouse conserved AS-NMD events were enriched for genes associated with mRNA splicing (Figure 4E and Supplemental Table 5b). Human-specific SE-NMD genes were enriched for DNA repair and other processes (Supplemental Figure 11H and Supplemental Table 5c). In total, we identified 3,571 SE-NMD–regulated genes: 2,607 in the human brain (Supplemental Table 5d) and 1,697 in the mouse brain (Supplemental Table 2f), including 595 genes as neuronal genes, 308 genes with Gene4Epilepsy annotations, 96 genes associated with autism spectrum disorders (SFARI, score = 1 or syndromic = 1), and 353 genes associated with neurodevelopmental disorders (Figure 4F).

Suppressing naturally occurring AS-NMD exons has been shown to be a promising strategy to treat haploinsufficient diseases (37, 40). To identify AS-NMD in haploinsufficient genes, we first utilized the probability of loss-of-function intolerance (pLI) score, which measures the probability of being loss of function-intolerant based on large-scale whole-exome and whole-genome data (61, 62). For genes with pLI > 0.9, we identified 194 NMD_in and 217 NMD_ex events from human brain dynamic SEs (Supplemental Table 4a), including 83 events in 61 genes, such as *FOXP1* and *GRIA2*, that are causal for neurodevelopmental disorders (Supplemental Table 4b). The *FOXP1* NMD_in exon was expressed in early brain development and downregulated in later stages (Figure 4G and Supplemental Figure 11I), and 3 intronic *FOXP1* mutations may influence the NMD_in exon inclusion (Supplemental Figure 11J).

### Validation of AS-NMD exons in human iPSC-derived neurons.

**Figure 5:**
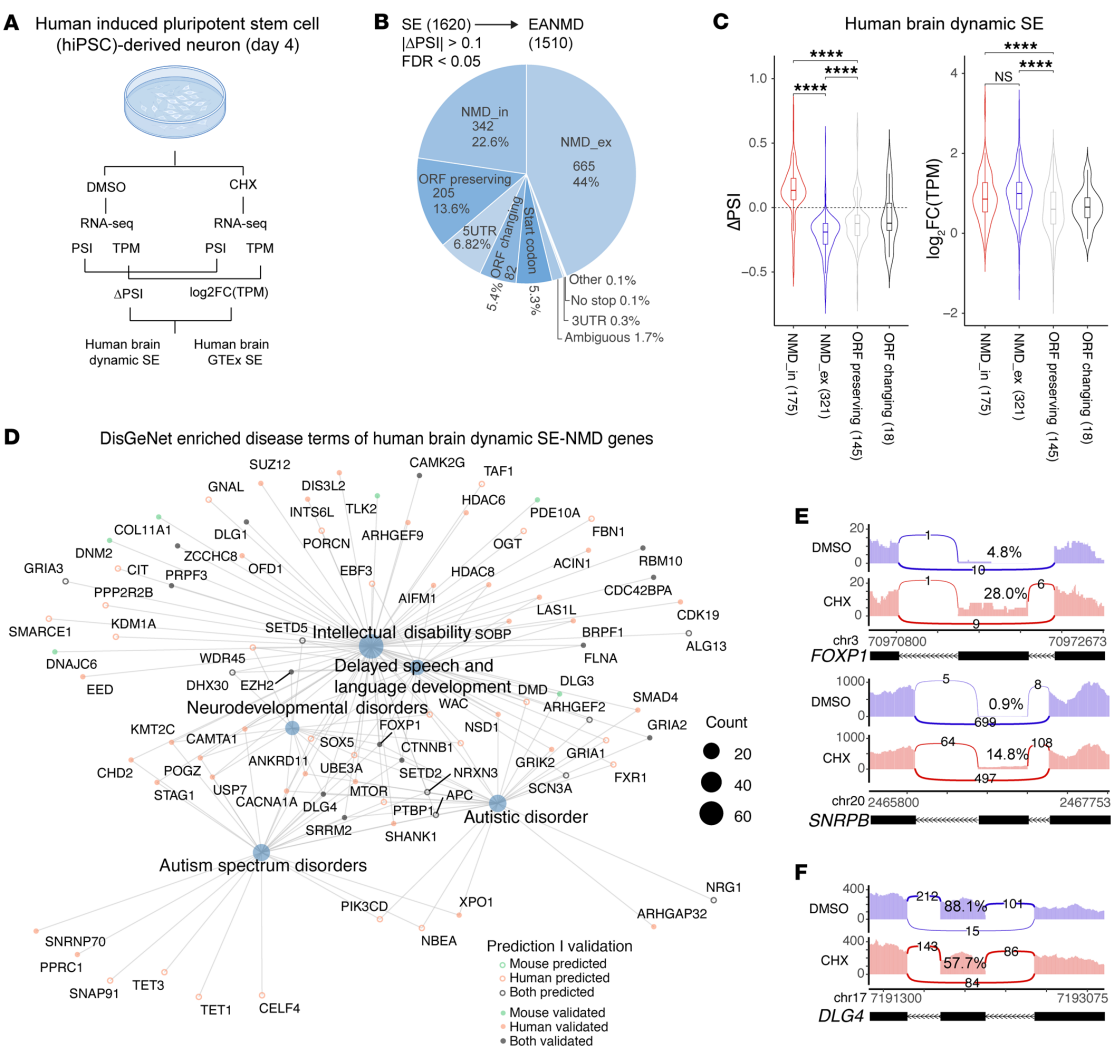
Validation of AS-NMD exons in human iPSC-derived neurons. (**A**) Human iPSC-derived neurons (iNeurons) were treated with DMSO (*n* = 3 biological replicates) or CHX (*n* = 3) for splicing analyses. (**B**) A pie chart showing differentially spliced SEs and the fractions of predicted AS-NMD exons when iNeurons were treated with CHX. (**C**) Violin plot of PSI and TPM for EANMD-predicted human brain SEs with significantly changed SEs (FDR < 0.05, minimum NMD score > 0.132) in iNeurons (NMD_in, *n* = 175; NMD_ex, *n* = 321; ORF preserving, *n* = 145; ORF changing, *n* = 18; 1-way ANOVA followed by Tukey’s multiple-comparison test); 141 NMD_in and 302 NMD_ex events had expected changes in CHX-treated iNeurons. (**D**) DisGeNET enrichment analysis for haploinsufficient genes (pLI > 0.9) regulated by SE-NMD exons in the human brain. Solid dots indicate that AS-NMD exons were validated with RNA-seq and/or RT-PCR. (**E**) Sashimi plots showing validated NMD_in exons in *FOXP1* and *SNRPB* (hg38). (**F**) Sashimi plot showing the NMD_ex exon in *DLG4* had decreased PSI in CHX-treated iNeurons (hg38).

We used iPSC-derived neurons to validate AS-NMD events identified in the human brain. Human iPSCs were induced to glutamatergic neurons (iNeurons) by NGN2 expression using an established protocol (63), treated with CHX, and subjected to RNA-seq (Figure 5A and Supplemental Figure 12, A and B). Among the 1,620 differentially spliced SEs upon CHX treatment, 66% of them were predicted to trigger NMD, showed higher mRNA levels (Figure 5B), and were enriched in mRNA processing, RNA splicing, and other processes (Supplemental Figure 12C). The predicted NMD events in CHX-treated iNeurons in general showed higher gene expression levels than host genes of ORF-preserving exons, and the trends of PSI value changes were as expected (Supplemental Figure 12D).

Importantly, 445 predicted AS-NMD events (NMD_in, 141; NMD_ex, 304) in the human brain were validated in the iNeuron dataset: the PSI values of NMD_in and NMD_ex exons were significantly increased or decreased, respectively, and host genes of the 445 exons showed higher mRNA levels in CHX-treated iNeurons (Figure 5C). Similarly, a subset of NMD events identified in the GTEx brain samples was validated (Supplemental Figure 12E). SE-NMD events in disease-associated genes (enriched in DisGeNet) were validated in human and/or mouse RNA-seq datasets or RT-PCR of independent samples (Figure 5D and Supplemental Table 4c). For instance, the NMD_in exons in *FOXP1*, *SNRPB* (Figure 5E), and other genes (Supplemental Figure 12F) showed increased inclusion in CHX-treated iNeurons; the NMD_ex exon in *DLG4* (Figure 5F) and additional disease-associated genes (Supplemental Figure 12G) showed higher exon skipping in CHX-treated iNeurons. We have deployed EANMDnet, an interactive online web portal, to make the results of this study accessible (Supplemental Figure 12H).

### Upregulation of GRIA2 by suppressing a poison exon.

**Figure 6:**
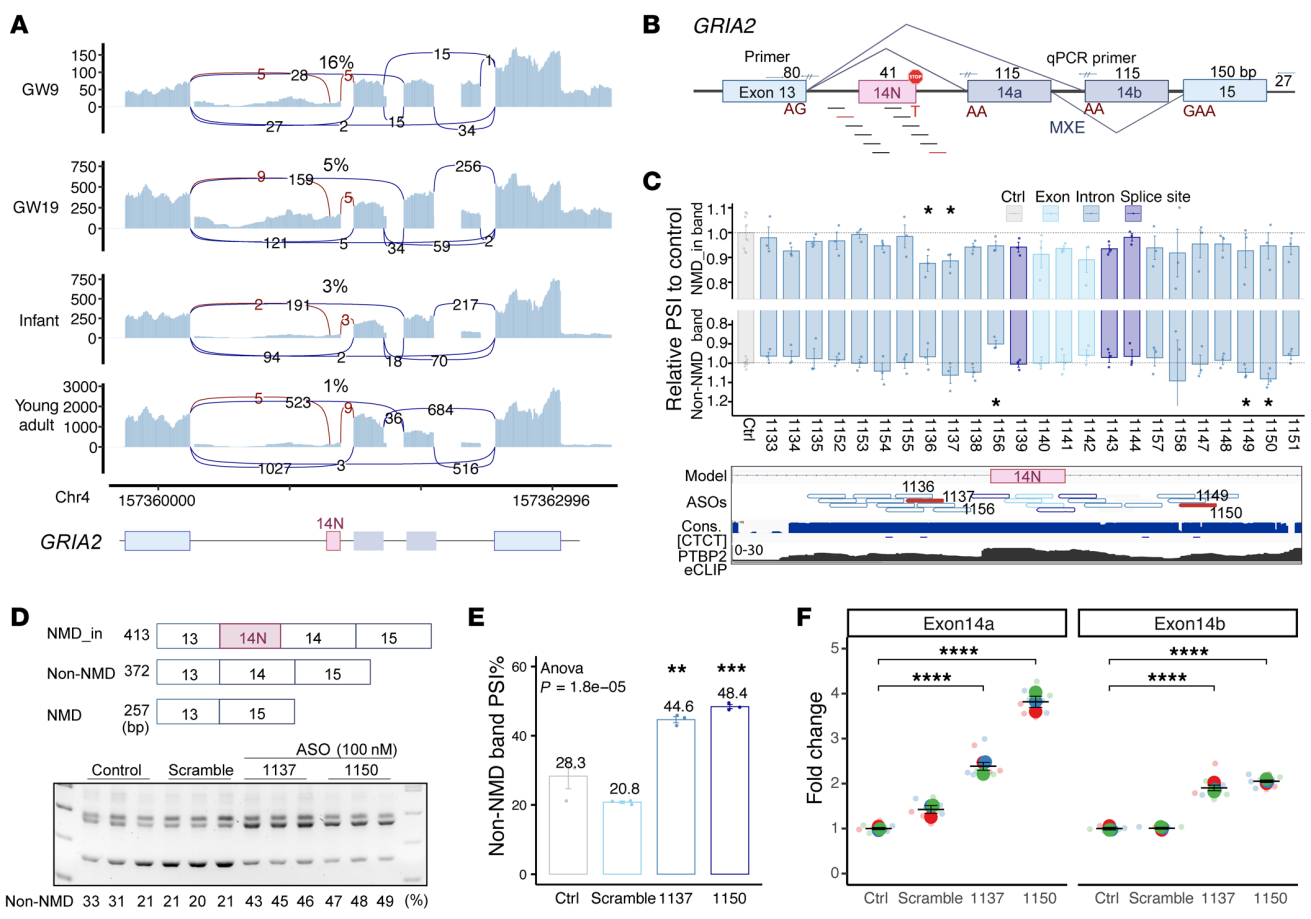
Upregulation of *GRIA2* expression by suppressing a poison exon. (**A**) Sashimi plots showing the *GRIA2* AS-NMD exon 14N inclusion in human brains (hg38). (**B**) Schematic of the *GRIA2* AS-NMD exon 14N locus, the minigene construct spanning exons 13–15, and the splice-switching ASOs. Exons 14a and 14b are the MXEs. (**C**) Effects of ASOs on *GRIA2* exon 14N inclusion in the HEK293T-GRIA2 stable cell line. Bar plots show FCs of PSI for NMD_in and non-NMD isoforms (with CHX treatment). Data represent mean ± SE, Wilcoxon’s rank-sum tests (control, *n* = 8; ASO, *n* = 3 biological replicates). The bottom tracks illustrate the positions of ASOs, PTBP1/2 binding motifs, and PTBP2 eCLIP tags. (**D** and **E**) RT-PCR (**D**) and quantification (**E**) results showing that ASO1137 and ASO1150 significantly increased the expression of the non-NMD *GRIA2* isoform. Data represent mean ± SE, 1-way ANOVA followed by Tukey’s multiple-comparison test, *n* = 3 biological replicates per group. (**F**) qPCR results showing that ASO1137 and ASO1150 significantly increased levels of functional *GRIA2* transcripts, for both exon 13-14a– and exon 13-14b–containing isoforms. Data represent mean ± SE, *n* = 3 biological replicates per group (3 technical replicates for each biological replicate), 1-way ANOVA followed by Tukey’s multiple-comparison test.

The *GRIA2* gene encodes the glutamate ionotropic receptor AMPA type subunit 2, and de novo *GRIA2* mutations have been reported to cause intellectual disability and neurodevelopmental deficits (64). Our analysis of the developing brains uncovered the developmentally regulated AS-NMD exon 14N, which is upstream of the flip-flop exons 14a/14b and introduces premature translational termination codons (Figure 2, F and G, Figure 6A, and Supplemental Figure 13, A–C). We constructed a stable cell line, HEK293T-GRIA2, that expressed the *GRIA2* minigene and recapitulated exon 14N inclusion (Figure 6B and Supplemental Figure 13, D and E). The exon 14N inclusion was increased upon CHX treatment, suggesting exon 14N inclusion triggers mRNA decay (Figure 2G and Figure 6).

Because multiple pathogenic *GRIA2* mutations cause loss of function (64), we sought to restore its expression by suppressing the AS-NMD exon 14N splicing/inclusion with antisense oligonucleotides (ASOs; Supplemental Table 7). Using window-sliding and BWA alignment filtering (65), we designed and screened 24 ASOs that uniquely mapped to the human genome (Figure 6B). ASOs 1136, 1137, 1149, and 1150 reduced exon 14N inclusion and increased the ratio of non-NMD transcripts in the stable cell line (Figure 6C and Supplemental Figure 13E). ASOs 1136 and 1137 targeted the upstream intron of exon 14N and overlapped with core PTBP motifs, suggesting the ASOs may interfere with PTBP1/2 binding. The top 2 ASOs, 1137 and 1150, were further validated in the HEK293T-GRIA2 stable cells and the SH-SY5Y cell line, where both ASOs suppressed exon 14N inclusion and significantly increased the ratio of non-NMD isoforms (Figure 6, D and E, and Supplemental Figure 13F). The RT-qPCR results showed that the functional non-NMD isoforms were increased up to 3.8-fold (ASO1150), with the non-NMD isoforms using more exon 14a than exon 14b (Figure 6F). Additionally, ASOs 1137 and 1150 showed dose-dependent effects: higher ASO doses resulted in higher ratios of functional isoforms in both HEK293T-GRIA2 stable cells and SH-SY5Y cells (Supplemental Figure 13, G–J). These results suggest that ASOs 1137 and 1150 redirect *GRIA2* splicing to functional isoforms and can potentially alleviate *GRIA2* haploinsufficient conditions.

## Discussion

We present a bioinformatic tool, EANMD, to identify AS events that trigger NMD. We analyzed developing mouse and human brains and uncovered thousands of developmentally regulated AS-NMD events. Interestingly, AS-NMD suppresses neuronal genes during brain development and is regulated by RBPs such as Ptbp1/2 and SR proteins. We further nominate potential therapeutic targets for haploinsufficient diseases and report ASOs that upregulate *GRIA2* expression by redirecting splicing.

Analyses of NMD exons in the developing brains indicated that AS-NMD critically regulates synaptic genes. The EANMD analysis recapitulated previously reported AS-NMD exons in *Psd95* (*Dlg4*) and *Syngap1* (40, 66). This study significantly expanded this group of genes related to the synapse. Further enrichment analyses showed that AS-NMD targets cellular components such as transporter complex and neuronal cell body and regulates biological processes such as trans-synaptic signaling and second messenger–mediated signaling. GSEA showed that NMD substantially regulates channel activity (Supplemental Table 2). Conversely, we also recapitulate AS-NMD exons in genes such as *Flna* (27) and *Bak1* (29) that were increasingly included during brain development. Additionally, we found 24 NMD_in genes related to chromosome organization (Figure 2E), which is consistent with a previous report (25). While AS-NMD was suggested to suppress non-neuronal genes in cultured neurons (53), our analyses of mouse and human brains indicate that AS-NMD plays an important role in suppressing neuronal genes during development. Taken together, developmentally regulated AS-NMD shapes the transcriptome in brain cells.

SR proteins are master splicing regulators, and most SR genes harbor ultraconserved AS-NMD exons for homeostatic or epistatic gene regulation (22, 67). Interestingly, SR protein binding motifs, such as those of Srsf10, Srsf3, and Srsf5, were enriched for AS-NMD events in the developing mouse brain. These observations suggest that SR proteins may function as hub regulators to selectively control neuronal genes through AS-NMD. Our analysis identified dozens of AS-NMD exons, such as the ones in *Iqgap1* and *Rock1*, that are directly targeted by Ptbp1/2 proteins. Interestingly, the Ptbp1/2 binding motifs are enriched inside the AS-NMD exons instead of their upstream introns. Our results also suggest additional *cis*-regulatory sequences of AS-NMD.

The presence of MXEs can introduce ambiguity for NMD prediction. We incorporated MXE references and employed an upstream-downstream count balance approach to mitigate their impact. However, multiple MXE events were still predicted to trigger NMD, such as *Dlg1* (chr16:31847635-31847669), *Gria2* (chr3:80690403-80690518), and *Gria4* (chr9:4424319-4424454). Close examination of RNA-seq reads and RT-PCR validation showed that MXE exons could be simultaneously included and trigger NMD (such as the *Gira2* and *Gria4* downstream MXEs in Figure 2, F and G). Indeed, the MXE exons in *Pkm2* have been reported to trigger NMD when both were included (68). MXEs are frequently seen in neuronal genes, and our observations indicate that their simultaneous inclusion can induce NMD and may be targeted for gene regulation.

Analysis of human brains identified thousands of AS-NMD events, some of which were region specific. Independent prediction of conserved mouse and human NMD exons showed high concordance, suggesting the reliability of EANMD. We identified AS-NMD exons in haploinsufficient genes, such as *GRIA2*, as potential therapeutic targets to treat human diseases. We identified ASOs that upregulated *GRIA2* expression by redirecting NMD isoforms to functional forms in cultured cells. In summary, this work presents a resource for investigating the functions of AS-NMD in brain development and nominates splice-switching targets for the treatment of neurodevelopmental disorders.

Our study has limitations. (a) Distant and coordinated splicing events in the same gene may be inaccurately annotated as AS-NMD exons. The challenge posed by complex isoforms, such as coordinated splicing events and alternative start codons, may be addressed by long reads that cover full-length transcripts. Our recent long-read analysis of human cerebral organoids identified coordinately spliced exons that are far apart in the same transcript (8), and in this study, long-read sequencing confirmed a subset of nonannotated AS-NMD events. Future computational and experimental investigations are needed to fully understand the functions of complex splice isoforms. (b) The *GRIA2* ASOs reported here would benefit from in vivo testing in human disease models to assess their safety, efficacy in gene regulation, and rescue effects on animal phenotypes.

## Methods

### Sex as a biological variant.

Sex was not considered a biological variable in this study.

### RNA-seq of Upf1-KD, Ptbp1/2-KD, and iPSC-derived neuron samples.

We knocked down *Upf1* using siRNAs in Neuro2a cells, as reported before (27). We performed RNA-seq on both control and *Upf1*-KD cells, using 2 biological replicates for each condition. Briefly, RNA was extracted from cultured cells using RNeasy Mini Kits (Qiagen, 74104). Sequencing libraries were built using the Illumina TruSeq Stranded Total RNA Library Prep Kit and sequenced on the Illumina HiSeq 2500 with 75 bp paired-end reactions.

Primary neurons from E16.5 CD1 mouse dorsal cortices were dissociated with papain (Worthington), resuspended, and cultured in neurobasal medium with GlutaMax, N2, and B27 supplements. On the first day in vitro, primary neurons were treated with 50 μg/mL CHX (Sigma-Aldrich, C4859) or an equal concentration of DMSO as control for 8 hours before RNA extraction with the Quick-RNA MiniPrep Kit (Zymo) and sequencing (75 bp paired end). We knocked down *Ptbp1* and *Ptbp2* genes separately and in combination using shRNAs (lentivirus) in Neuro2a cells, as reported before (27). Cells were harvested 5 days after transduction for RNA extraction with the Quick-RNA MiniPrep Kit. RNA-seq libraries (2 biological replicates for each) were prepared using the Illumina TruSeq Stranded mRNA Library Prep Kit and Illumina NextSeq 500 (100 bp paired end).

Human iPSCs (69) were cultured and induced into neurons as reported before (8). iNeurons (day 4) were treated with CHX (200 μg/mL for 5 hours, 3 replicates in parallel with 3 DMSO controls) in 12-well plates with 1 mL culture media in each well, followed by RNA extraction and RNA-seq library preparation (Illumina Stranded mRNA Prep Ligation Kit, 20040534). RNA-seq libraries were sequenced on an Illumina NovaSeq (50 bp paired end) at the University of Chicago.

### Long-read sequencing of E14.5 and E18.5 mouse brains.

The full-length cDNAs (SMART RT and amplification) (70) of E14.5 and E18.5 C57BL/6J mouse brains were first captured using Drop-seq, and the untagmented cDNA libraries were subjected to long-read sequencing with PacBio Sequel I. PacBio libraries were prepared with 1 μg amplified cDNA using the SMRTbell Express Template Prep Kit V2.0. The E14.5 dataset was published previously (24). The E18.5 dataset is available in the NCBI Sequence Read Archive (SRR29089092).

### Detection of dynamic SEs during brain development.

The mouse ENCODE RNA-seq dataset was used to study splicing profiles of the embryonic central nervous systems (E11.5, E14.5, and E18.5) and 20 adult (8 weeks) tissues, including the adrenal glands, bladder, colon, brain (cortex, frontal lobe, and cerebellum), genital fat pad, heart, kidney, large intestine, liver, lung, mammary gland, ovary, placenta, subcutaneous fat pad, small intestine, spleen, testis, and thymus (2 replicates each, GEO accession GSE36025) (52). The raw FASTQ files were cleaned using fastp (71). We mapped the reads to the mouse reference genome (GRCm38/mm10) using the STAR v2.7.9 aligner (72) and identified splicing events using the rMATS default parameters (--chimSegmentMin 2 --outFilterMismatchNmax 3 --alignEndsType EndToEnd --outSAMstrandField intronMotif --alignSJDBoverhangMin 3 --alignIntronMax 299999) (73). We used a branch version of rMATS turbo v4.1.1 (individual counts) to count reads supporting the upstream junction (UJC), downstream junction (DJC), and skipping junction separately. We applied a filter of |ΔPSI| > 0.10, FDR < 0.05, and excluded MXEs by (a) ensuring the average minimum reads of UJC and DJC counts were ≥ 2 and (b) setting the ratio of Min(UJC, DJC)/Max(UJC, DJC) > 0.05. We applied the same parameters to the human forebrain samples (PRJEB26969). There were 32 human forebrain samples across 14 time points after quality control (GW4, GW7, GW8, GW9, GW11, GW13, GW19, infant, toddler, school-age child, adolescent, young adult, middle-aged adult, and elderly).

### Annotation of AS-NMD events.

We developed EANMD to predict AS-NMD events (https://github.com/dontkme/EANMD; commit ID 86a6496). EANMD accepts GENCODE-style GTFs as reference input (other GTFs could be adapted with the GTFaddExonNums.pl script) and supports AS-NMD annotation using multiple threads. For input AS events, rMATS output (SE.JCEC.txt) was processed using the GetSEinput script, and SUPPA2 output was converted using the TransIOE2SEinput script. The EANMD combined output was filtered with the EANMDFilterOut script to exclude noncanonical start codons (non-ATG) and MXE events. Final annotation summaries were generated using the EANMDflagcount.R script. Known MXEs were annotated using the MISO MXE reference (version 2) (74). We used the GRCh38 and GENCODE v38 annotations (75) for human SE identification and the GENCODE v43 annotation for SUPPA2 AS detection and NMD prediction. Cross-species exon coordinate conversions were performed using UCSC LiftOver (76).

### Machine learning model for NMD efficiency.

The EANMD intermediate outputs were used to train a machine learning model for predicting NMD efficiency. The XGBoost model was constructed with the R xgboost package with the following parameters: seed = 321, max_depth = 7, eta = 0.05, subsample = 0.5495662, colsample_bytree = 0.8133278, nthread = 4, nrounds = 50000, early_stopping_rounds = 100, alpha = 0, lambda = 1, gamma = 0.2, min_child_weight = 1, eval_metric = “rmse,” and objective = “reg:squarederror.” Optimal parameters were determined using the ParBayesianOptimization R package. The dataset was randomly split into training and testing sets with a ratio of 0.85:0.15 (seed = 321). As the training dataset was balanced (NMD, 102; non-NMD, 94), only the area under the ROC curve (AUC) was evaluated by ROCR (77) and pROC (78) packages. The final model incorporated 9 features from 21 tested features, including (a) minimum stop codon position fraction of isoform (Min stop Pos F), (b) maximum stop codon distance to the last exon-exon junction (Max DJ), (c) interaction between Min stop Pos F and Max DJ (Min stop Pos F * Max DJ), (d) 3′-UTR length, (e) SE length, (f) last exon number minus SE number (SE Pos to LE), (g) minimum stop codon position to the isoform end (Min stop Pos to End), (h) exon number of the start codon exon (Start_exon), and (i) average minimum free energy per nucleotide of the original 3′-UTR (Ori 3′-UTR3seq MFE per nt). Minimum free energy was calculated by RNAfold (79). Our EANMDflagcount_withUTRMFE.R script applied the XGBoost model to generate the NMD score for each AS event.

### EANMD performance evaluations.

We used the *Upf1*-KD dataset to evaluate EANMD results. We manually confirmed the SE events’ NMD flags by the 50 nt rule and the GENCODE M25 transcript_type (nonsense_mediated_decay). NMD Classifier analysis (43) was performed with the HISAT2-Stringtie-NMD Classifier workflow (80). Because the NMD Classifier only uses the Ensembl and NCBI annotations, we used the Ensembl-release 102 Mus_musculus GTF as the reference (81). SpliceTools prediction was done by querying the same SE rMATS output file to the server (42). Processed rates (coverage), precision (true positive [TP]/(TP + false positive)), recall (TP/(TP + false negative)), and F1 scores ((2 × precision × recall)/(precision + recall)) were calculated to evaluate performances.

### RNA-seq downstream analysis.

Differentially expressed gene analysis was performed using the STAR-FeatureCounts-DESeq2 workflow (72, 82, 83). Briefly, short reads were aligned to the reference genome using the STAR aligner with the same parameters as AS event detection, and the gene expression quantification was performed using FeatureCounts. DESeq2 was then used to identify differentially expressed genes based on the criteria of adjusted *P* < 0.001 and |log_2_FC|> 1 as default. TPMs were calculated using TBtools (84). Intron retention analysis was performed using IRFinder (85). The original transcript and modified transcript expression level (TPM) was quantified with the salmon-tximport workflow. PSI pattern clustering was based on pairwise FCs between near time points, where FC > 1.1 was defined as increased, FC < 0.9 as decreased, and 0.9 ≤ FC ≤ 1.1 as unchanged. Trends across developmental stages from E11.5 to E18.5 were also incorporated into the clustering. The raw long-read sequences generated by PacBio were processed as previously reported (8). The NMD status of long-read isoforms was predicted with SQANTI3 (86) and EANMDcheckGTFNMD.pl script (this study). Long-read AS events were detected by FLAIR align, correct, collapse (87), and the SUPPA2 generateEvents workflow (88). Sashimi plots were generated using the Integrative Genomics Viewer (89) and ggshashimi (90). Gene Ontology enrichment analysis was performed using the PANTHER online tool (91). Synaptic gene enrichment analysis was performed with SynGO (92). GSEA was performed on WebGestalt (93) using ΔPSI as the ranking value; NMD_ex ΔPSI was reversed in all NMD analysis. Disease enrichment analyses were performed with Enrichr (94) using the DisGeNET database (95). Coordinates of SEs and their flanking 20-nt intronic sequences were intersected with the ClinVar pathogenic/likely pathogenic de novo mutation list (SNP/Indel, 17910, July 22, 2024). Disease causal genes were annotated by OMIM (May 19, 2025), Genes4Epilepsy (v2025-03) (96), and SFARI (v2025-04-03, score = 1 and syndromic = 1).

### Sequence feature analysis.

Dynamic SE events in mice were analyzed by retrieving the SE, upstream exon, downstream exon, upstream intron, and downstream intron sequences using BEDTools (97). The UCSC bigWigAverageOverBed tool was used to obtain the phastCons conservation score.

### CLIP-seq peak calling and RBP motif enrichment analysis.

To identify the binding targets of Ptbp1 and Ptbp2, we reanalyzed CLIP-seq data using previously published datasets for iCLIP of Ptbp1 in mouse embryonic stem cell (GEO GSM1828887), mouse neural progenitor cells (GEO GSM1828888), HITS-CLIP of Ptbp2 in mouse neural progenitor cells (GEO GSE47564), and human PTBP2 eCLIP (41). CLIP-seq data analysis was conducted using the CTK pipeline (98). To identify the distribution of CLIP peaks on SE upstream and downstream regions, we used rMAPS2 CLIP map analysis (57). Other RBP motif enrichment of SE-NMD events was analyzed using rMAPS2 with the default settings (intron, 250; exon, 50; sliding window size, 50; interval, 1). XSTREME was used for motif discovery and enrichment analysis (99) using the second group as background in each comparison.

### Cell culture.

HEK293T (CRL-3216) and SH-SY5Y (CRL-2266) cell lines were obtained from the American Type Culture Collection. HEK293T cells were cultured in DMEM (Gibco, catalog 11965-092) supplemented with 10% FBS (Gibco, catalog A31605-01) and 100 U/mL penicillin-streptomycin (Gibco, catalog 15140-122) at 37°C in a humidified incubator with 5% CO_2_. SH-SY5Y cells were cultured in a 1:1 mixture of DMEM and Ham’s F12 (Gibco, catalog 11320-033) supplemented with 10% FBS and 100 U/mL penicillin-streptomycin.

### The GRIA2 minigene reporter in HEK293T cells.

To generate a *GRIA2* minigene splicing reporter construct, a genomic fragment spanning exons 13 to 16 of the human *GRIA2* gene was amplified (primers CH1131 and CH1132) and cloned into the pZ070 plasmid using Gibson Assembly (New England Biolabs, catalog E2611L). The construct was verified by Sanger sequencing and transfected into HEK293T cells using Lipofectamine 3000 (Thermo Fisher Scientific, catalog L3000-008) according to the manufacturer’s instructions. Stable cells were selected with 2 μg/mL puromycin for 7 days prior to downstream analysis.

### ASO design and screening.

ASO window sliding was designed with 20 nt length and 6 nt offsets. ASO sequences were aligned to the human genome (hg38) with BWA (aln -n 0.06), and only uniquely mapped sequences were kept (65). All ASOs were chemically modified with 2′-*O*-methoxyethyl with the phosphorothioate backbone (IDT). ASOs were dissolved in Dulbecco’s PBS (Gibco, catalog 14190-144). HEK293T-GRIA2 and SH-SY5Y cells were seeded in 24-well plates and transfected with Lipofectamine RNAiMAX (Thermo Fisher Scientific, catalog 13778-150) according to the manufacturer’s instructions. After 18 hours of incubation, CHX (Sigma-Aldrich, catalog C4859) was added to the culture medium at a final concentration of 100 μg/mL, and cells were incubated for an additional 6 hours. Cells were harvested after a total of 24 hours for downstream analysis. ASO sequences are listed in Supplemental Table 7.

### RNA extraction and RT-PCR/qPCR.

Total RNA was extracted using TRIzol reagent (Thermo Fisher Scientific, catalog 15596018) and the Direct-zol RNA Microprep Kit (Zymo Research, catalog 11-330MB), following the manufacturers’ protocols. Reverse transcription was performed using SuperScript IV (Thermo Fisher Scientific, catalog 18-090-050). qPCR was performed on a QuantStudio 3 Real-Time PCR System (Applied Biosystems) using Luna Universal qPCR Master Mix (New England Biolabs, catalog M3003E). Primers for PCR are listed in Supplemental Table 7.

### Statistics.

Statistical analysis was performed using R (v4.2.3) and GraphPad (v8.0.2). Heatmaps were generated using the pheatmap and ComplexHeatmap R packages (100). UpSet plots were created using the UpSet R package (101). Other figures were plotted with ggplot2 (102) and ggpubr (103). Pearson’s correlation was used to analyze correlations between variables. Pairwise comparisons were performed using a 2-sided Student’s *t* test or 2-sided Wilcoxon’s rank-sum test. ANOVA, followed by Tukey’s multiple comparisons, was used for comparisons of multiple groups. *P* values are presented as follows: *P ≤ 0.05, **P ≤ 0.01, ***P ≤ 0.001, and ****P ≤ 0.0001.

### Study approval.

Analyses of deidentified RNA-seq samples were approved (exempted) by the University of Chicago Institutional Review Board.

### Data availability.

RNA-seq data reported in this study are available under NCBI BioProject ID PRJNA1079809 and NCBI GEO GSE324908. The EANMD software is available on GitHub: (https://github.com/dontkme/EANMD; commit ID 86a6496) and CodeOcean (http://codeocean.com/capsule/3402124/tree). The interactive EANMDnet web page is https://zlab1.shinyapps.io/EANMDnet. Values of reported data points are provided in the Supporting Data Values file. Raw gel images are available in the supplemental materials.

## Author contributions

KH led the EANMD program development, ASO design, and data analyses. RY validated AS-NMD events and prepared samples from iPSC-induced neurons for RNA-seq. JQ performed the ASO screening and ASO-associated experiments. XF prepared the *siUpf1*, CHX, and *shPtbp1/2* RNA-seq libraries. KJL and JT validated the *GRIA2* NMD exon. YY processed the long-read data. XZ conceived and supervised the project. KH and XZ wrote the manuscript with input from all coauthors.

## Conflict of interest 

Findings in this work are included in a provisional patent, “Antisense Oligonucleotides and Uses Thereof for Splicing Regulation,” authored by XZ, KH, JQ.

## Funding support

This work is the result of NIH funding, in whole or in part, and is subject to the NIH Public Access Policy. Through acceptance of this federal funding, the NIH has been given a right to make the work publicly available in PubMed Central.

- Simons Foundation Autism Research Initiative (Pilot Progression 00008999) to XZ.
- National Institute of Mental Health (R01 MH130594) to XZ.
- National Institute of General Medical Sciences (R35 GM152177) to XZ.

## Supplementary Material

Supplemental data

Unedited blot and gel images

Supplemental table 1

Supplemental table 2

Supplemental table 3

Supplemental table 4

Supplemental table 5

Supplemental table 6

Supplemental table 7

Supporting data values

## Version 1 02/12/2026

In-Press Preview

## Version 2 04/15/2026

Electronic publication
