# Supplementary material for: Alternative splicing–triggered mRNA decay informs splice-switching targets for neurodevelopmental disorders
Source: J Clin Invest. 2026 Feb 12;136(8):e197271. doi: 10.1172/JCI197271 (PMC13078869; doi:10.1172/JCI197271)
Supplement: Supplemental data [file jci-136-197271-s058.pdf]

Alternative Splicing-triggered mRNA Decay Informs Splice-switching Targets for Neurodevelopmental Disorders

Kaining Hu[1], Runwei Yang[1], Jiaming Qiu[1], Xinran Feng[1], Kayleigh J. LaPre[1], Jessica Tanouye[1], Yalan Yang[1], Xiaochang Zhang[1,*]

1. Department of Human Genetics, The Neuroscience Institute, University of Chicago, Chicago, IL 60637, USA

* Correspondence: Cummings Life Science Center 507A, 920 E. 58th St., Chicago, IL 60637; Phone: 773-834-5369; Email: xczhang@uchicago.edu (X. Z.)

Supplemental Figures 1-13 and Figure Legends

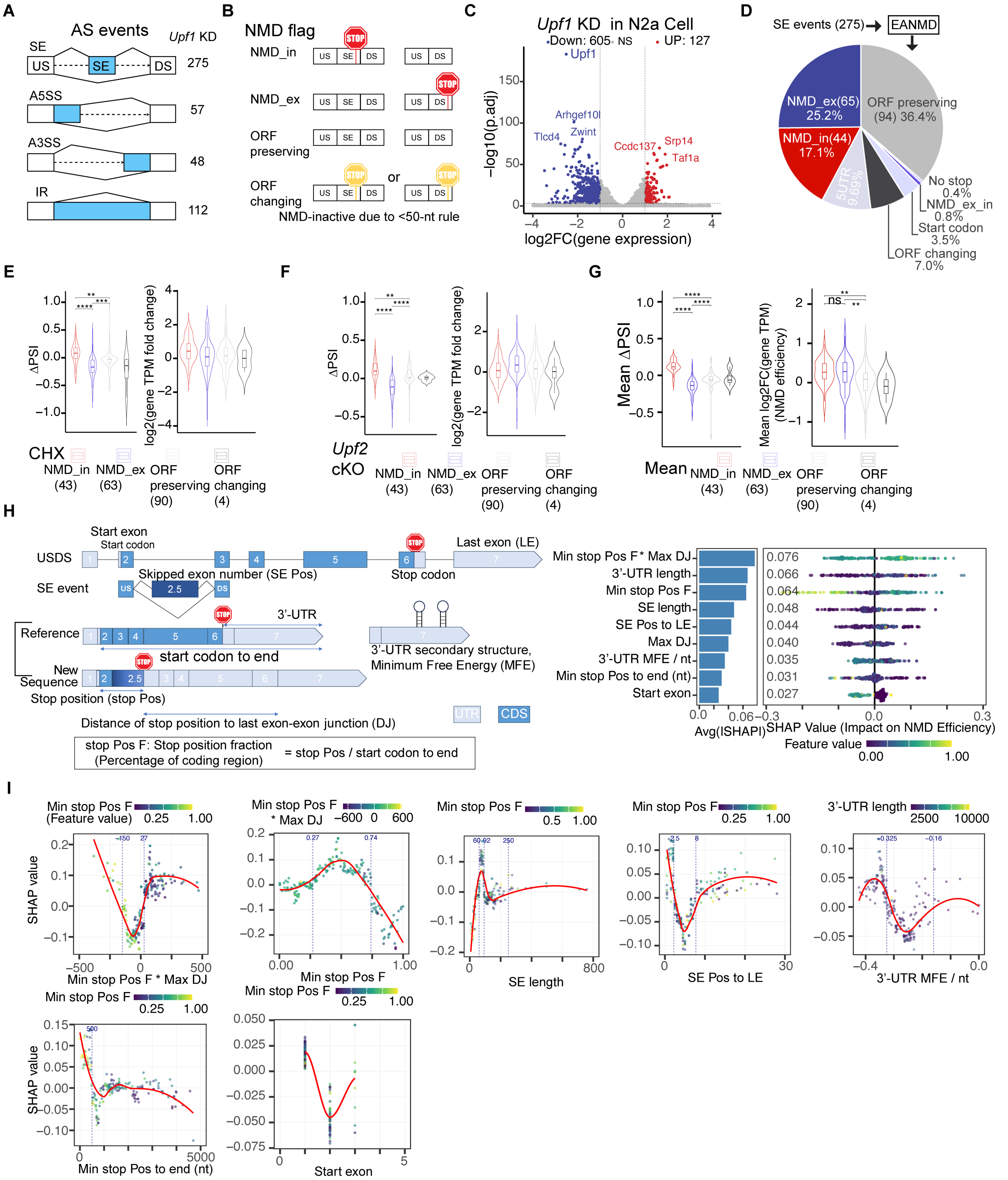

Supplemental Figure 1. Identifying AS-NMD events with EANMD.

(**A**) Alternative splicing (AS) events used for EANMD prediction. Right side: numbers of AS events detected in Neuro2a *Upf1* KD samples. SE: skipped exons. A5SS: alternative 5' splice site. A3SS: alternative 3' splice site. IR: intron retention.

(**B**) Schematic illustration of the four main AS-NMD flags: NMD_in, NMD_ex, ORF preserving, and ORF changing.

(**C**) Volcano plot showing differentially expressed genes (DEGs) in Neuro2a cells following *Upf1* KD (2 biological replicates per group, $|\log_2FC| > 1$ and adjusted $p < 0.001$).

(**D**) Pie chart showing the distribution of AS-NMD flags among differentially spliced SEs upon *Upf1* KD.

(**E**) ΔPSI (left, percent spliced in) and $\log_2FC$ in gene expression (TPM, right) for SE events in primary neurons treated with cycloheximide (CHX). One-way ANOVA followed by Tukey's multiple comparisons tes in E-G) t, NMD_in: n = 43, NMD_ex: n = 63, ORF preserving: n = 90, and ORF changing: n = 4.

(**F**) ΔPSI (left) and $\log_2FC$ in gene expression (TPM, right) for SE events in E13.5 *Upf2* conditional knockout (cKO) mouse brains (1). NMD_in: n = 43, NMD_ex: n = 63, ORF preserving: n = 90, and ORF changing: n = 4.

(**G**) Mean ΔPSI (left) and mean $\log_2FC$ in gene expression (TPM, right) across the three NMD inhibition conditions. NMD_in: n = 43, NMD_ex: n = 63, ORF preserving: n = 90, and ORF changing: n = 4.

(**H**) Schematic illustration of SE features used in the XGBoost-based model for NMD efficiency prediction (left). Summary plot of SHAP (SHapley Additive exPlanations) values showing the impact of individual features on the output NMD score (right).

(**I**) SHAP value distributions (y axis) and feature values (x axis) for seven contributors to NMD efficiency prediction: the minimum stop codon position fraction (*Min stop Pos F*), maximum stop distance to the last exon-exon junction (*Max DJ*), SE length, SE position relative to last exon (*SE Pos to LE*), 3′-UTR minimum free energy per nucleotide, minimum length of stop position to the transcript end and exon number of start codon exon.

A

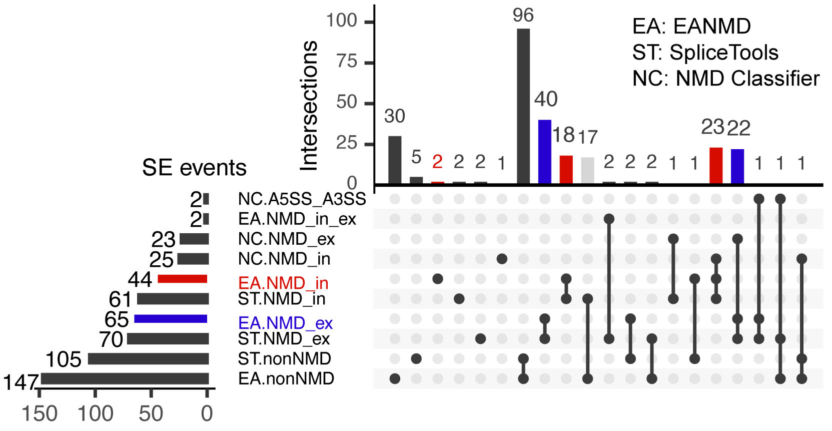

B

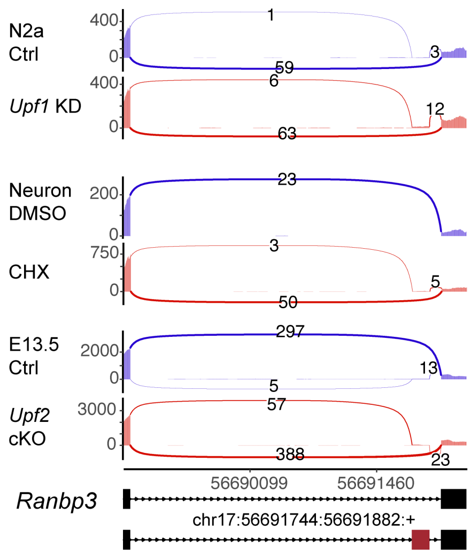

C

Prediction of 271 Coding Gene SE Events

		EANMD: 258		SpliceTools: 236		NMD Classifier: 50	
		NMD	Non-NMD	NMD	Non-NMD	NMD	Non-NMD
Only 50-nt rule — Actual	NMD(118)	109	9	112	6	49	69
	Non-NMD (153)	2	151	18	135	1	152
	Precision	0.982		0.862		0.980	
	Recall	0.924		0.950		0.415	
	F1	0.952		0.903		0.582	
		NMD	Non-NMD	NMD	Non-NMD	NMD	Non-NMD
50-nt rule & NMD sensitive — Actual	NMD(88)	71	17	84	4	40	48
	Non-NMD (183)	8	175	46	137	10	173
	Precision	0.899		0.646		0.800	
	Recall	0.807		0.955		0.455	
	F1	0.850		0.771		0.580	

Supplemental Figure 2. Benchmarking of EANMD predictions.

(**A**) UpSet plot comparing AS-NMD prediction between EANMD, SpliceTools, and NMD Classifier.

(**B**) Sashimi plot showing an AS-NMD event in *Ranbp3*, identified by EANMD but not the other tools. The AS-NMD prediction was supported by transcriptomic data from *Upf1* KD in Neuro2a cells, CHX-treated primary neurons, and E13.5 *Upf2* cKO mouse brains.

(**C**) Comparative performance of EANMD, SpliceTools, and NMD Classifier on *Upf1* KD datasets. Top: performance based on the 50-nt rule. Bottom: performance based on 50-nt rule combined with the XGBoost model derived NMD efficiency.

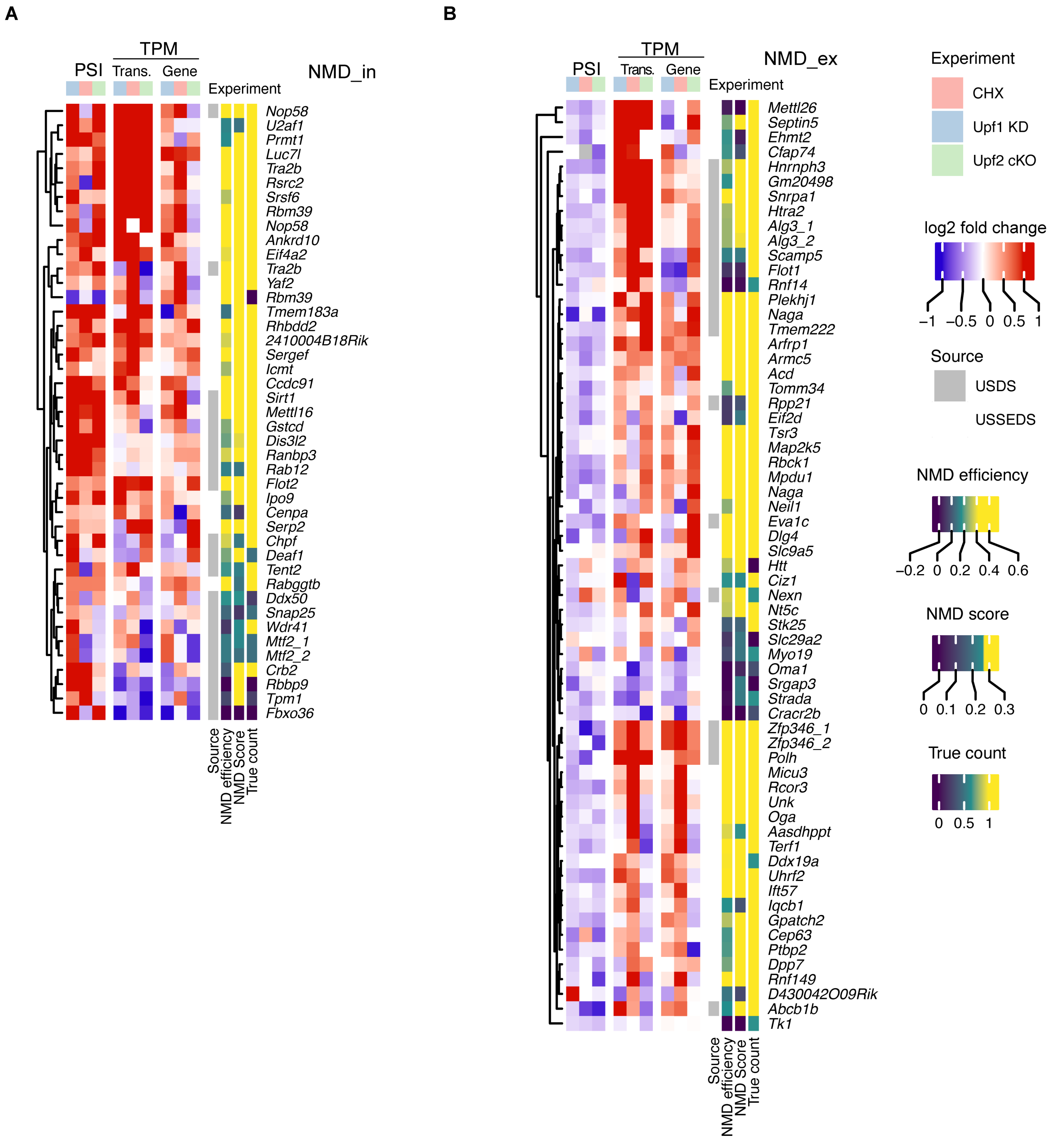

Supplemental Figure 3. EANMD-predicted NMD events following *Upf1* KD.
(**A** and **B**) Heatmaps displaying PSI and $\log_2$FC(transcript- and gene-level TPM) for predicted NMD_in (**A**) and NMD_ex (**B**) events in *Upf1* KD samples. The panel also includes $\log_2$FC (NMD efficiency), XGBoost model predicted NMD efficiency (NMD score), and the PSI-TPM concordance rate (True count, NMD_in: PSI increase and TPM increase; NMD_ex: PSI decrease and TPM increase).

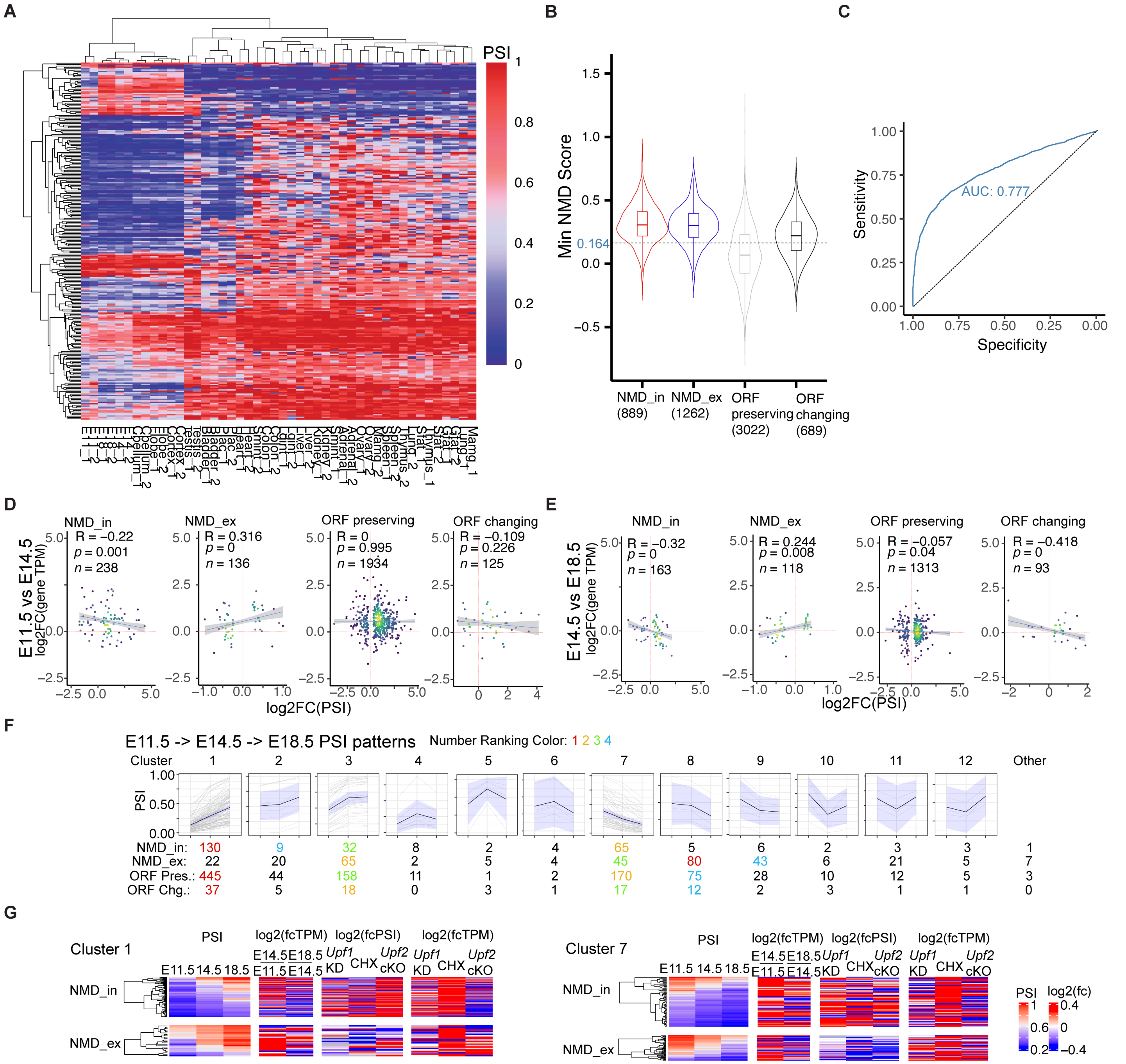

Supplemental Figure 4. Dynamic SE-NMD events in mice.

(**A**) Heatmap showing a subset of brain-specific SE events (|mean PSI of E11.5 and E14.5 – mean PSI of adult non-brain tissues| ≥ 0.25, n = 260 exons).

(**B**) Boxplot of EANMD-predicted NMD scores across different NMD flags in the mouse brain.

(**C**) Classification of NMD versus non-NMD using an optimized threshold for minimum NMD score per AS events (0.164), yielding an AUC of 0.777.

(**D** and **E**) Correlations between log_2FC(PSI) of NMD events and corresponding gene-level TPM fold changes from E11.5 to E14.5 (**D**, NMD_in: n = 238, NMD_ex: n = 136, ORF preserving: n = 1934, and ORF changing: n = 125) and from E14.5 to E18.5 (**E**, NMD_in: n = 163, NMD_ex: n = 118, ORF preserving: n = 1313, and ORF changing: n = 93). Non-NMD events (low NMD score, ORF preserving or ORF changing) showed no significant correlation (Pearson correlation).

(**F**) PSI trend clustering of predicted SE-NMD events across E11.5 to E18.5. Clusters 1–5 and 12 represent exons with increasing PSI during brain development (Up), while clusters 6–11 represent those with decreasing PSI (Down).

(**G**) Heatmaps showing PSI and corresponding gene TPM values for dynamically regulated SE events (left: Cluster 1 upregulated, right: Cluster 7 downregulated) across three NMD perturbation conditions: *Upf1* KD in Neuro2a cells, CHX treatment in primary neurons, and *Upf2* cKO in E13.5 mouse brains. NMD_in events showed increased PSI and NMD_ex exons showed decreased PSI after blocking NMD.

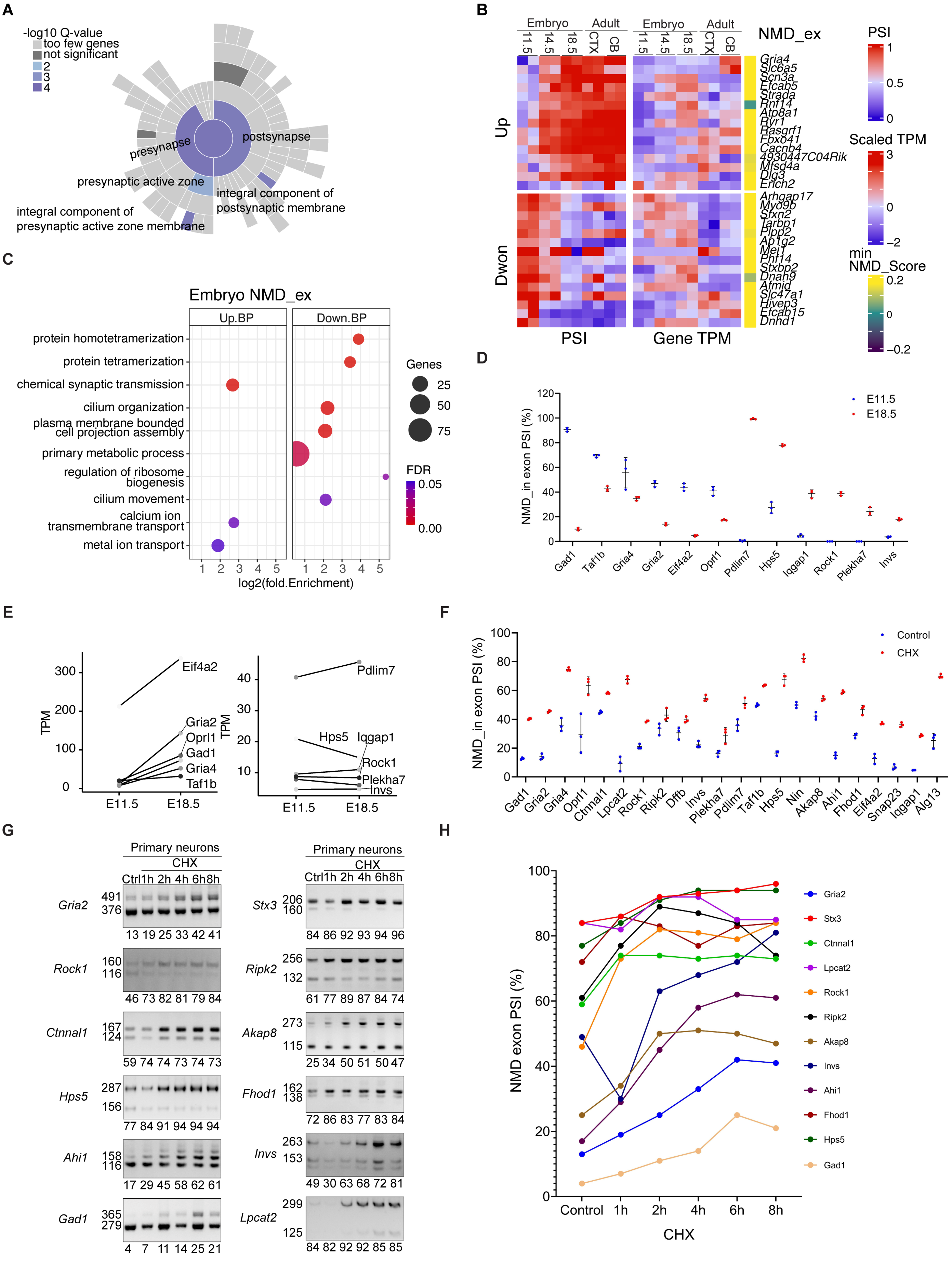
A
-log10 Q-value
too few genes
not significant
2
3
4
presynapse
postsynapse
presynaptic active zone
integral component of postsynaptic membrane
integral component of presynaptic active zone membrane
B
Embryo
Adult
11.5
14.5
18.5
CTX
CB
NMD_ex
PSI
Scaled TPM
min NMD_Score
Up
Dwon
Gria4
Slc6a5
Scn3a
Efcab5
Strada
Rnf14
Atp8a1
Ryr1
Rasgrf1
Fbxo41
Cacnb4
4930447C04Rik
Mfsd4a
Dlg3
Erich2
Arhgap17
Myo9b
Stxn2
Tarbp1
Plpp2
Ap1g2
Mei1
Phf14
Stxbp2
Dnah9
Afmid
Slc47a1
Hivep3
Efcab15
Dnhd1
PSI
Gene TPM
C
Embryo NMD_ex
Up.BP
Down.BP
protein homotetramerization
protein tetramerization
chemical synaptic transmission
cilium organization
plasma membrane bounded cell projection assembly
primary metabolic process
regulation of ribosome biogenesis
cilium movement
calcium ion transmembrane transport
metal ion transport
log2(fold.Enrichment)
Genes
FDR
D
NMD_in exon PSI (%)
E11.5
E18.5
Gad1
Taf1b
Gria4
Gria2
Eif4a2
Oprl1
Pdlim7
Hps5
Iqgap1
Rock1
Plekha7
Invs
E
TPM
Eif4a2
Gria2
Oprl1
Gad1
Gria4
Taf1b
Pdlim7
Hps5
Iqgap1
Rock1
Plekha7
Invs
E11.5
E18.5
F
NMD_in exon PSI (%)
Control
CHX
Gad1
Gria2
Gria4
Oprl1
Ctnnal1
Lpcat2
Rock1
Ripk2
Dffb
Invs
Plekha7
Pdlim7
Taf1b
Hps5
Nin
Akap8
Ahi1
Fhod1
Eif4a2
Snap23
Iqgap1
Alg13
G
Primary neurons
CHX
Ctrl 1h 2h 4h 6h 8h
Gria2 491 376
13 19 25 33 42 41
Rock1 160 116
46 73 82 81 79 84
Ctnnal1 167 124
59 74 74 73 74 73
Hps5 287 156
77 84 91 94 94 94
Ahi1 158 116
17 29 45 58 62 61
Gad1 365 279
4 7 11 14 25 21
Stx3 206 160
84 86 92 93 94 96
Ripk2 256 132
61 77 89 87 84 74
Akap8 273 115
25 34 50 51 50 47
Fhod1 162 138
72 86 83 77 83 84
Invs 263 153
49 30 63 68 72 81
Lpcat2 299 125
84 82 92 92 85 85
H
NMD exon PSI (%)
Control
1h
2h
4h
6h
8h
CHX
Gria2
Stx3
Ctnnal1
Lpcat2
Rock1
Ripk2
Akap8
Invs
Ahi1
Fhod1
Hps5
Gad1

Supplemental Figure 5. Validation of SE-NMD events in the mouse brain.

(A) SynGO enrichment analysis of genes with developmentally downregulated NMD_in or upregulated NMD_ex exons (E18.5 vs E11.5). All brain-expressed genes were used as the background set.

(B) Heatmap displaying the PSI values and scaled TPM of the top 15 and bottom 15 NMD_ex SE events showing the largest PSI changes from E11.5 to E18.5.

(C) Gene Ontology biological process (BP) enrichment for dynamic NMD_ex genes (E11.5 vs E18.5).

(**D**) RT-PCR validation (Figure 2F) and quantification of NMD_in events in mouse E11.5 and E18.5 dorsal forebrains. All pairwise comparisons are statistically significant ($p < 0.05$, t-test, $n = 3$ per group).

(**E**) Line plots showing transcript per million (TPM) changes of RT-PCR-validated NMD_in genes (Figure 2F). Left panel: genes with developmentally downregulated PSI; right panel: genes with increased PSI.

(**F**) RT-PCR validation and quantification of NMD_in events in mouse primary neurons under CHX treatment. All events are statistically significant ($p < 0.05$, t-test, $n = 3$ per group).

(**G**) RT-PCR validation of NMD_in events in mouse primary neurons upon CHX treatment for 0-8 hours (1 sample at each time point).

(**H**) The line plot showing the PSI trends of validated NMD_in events after CHX treatment in (**G**).

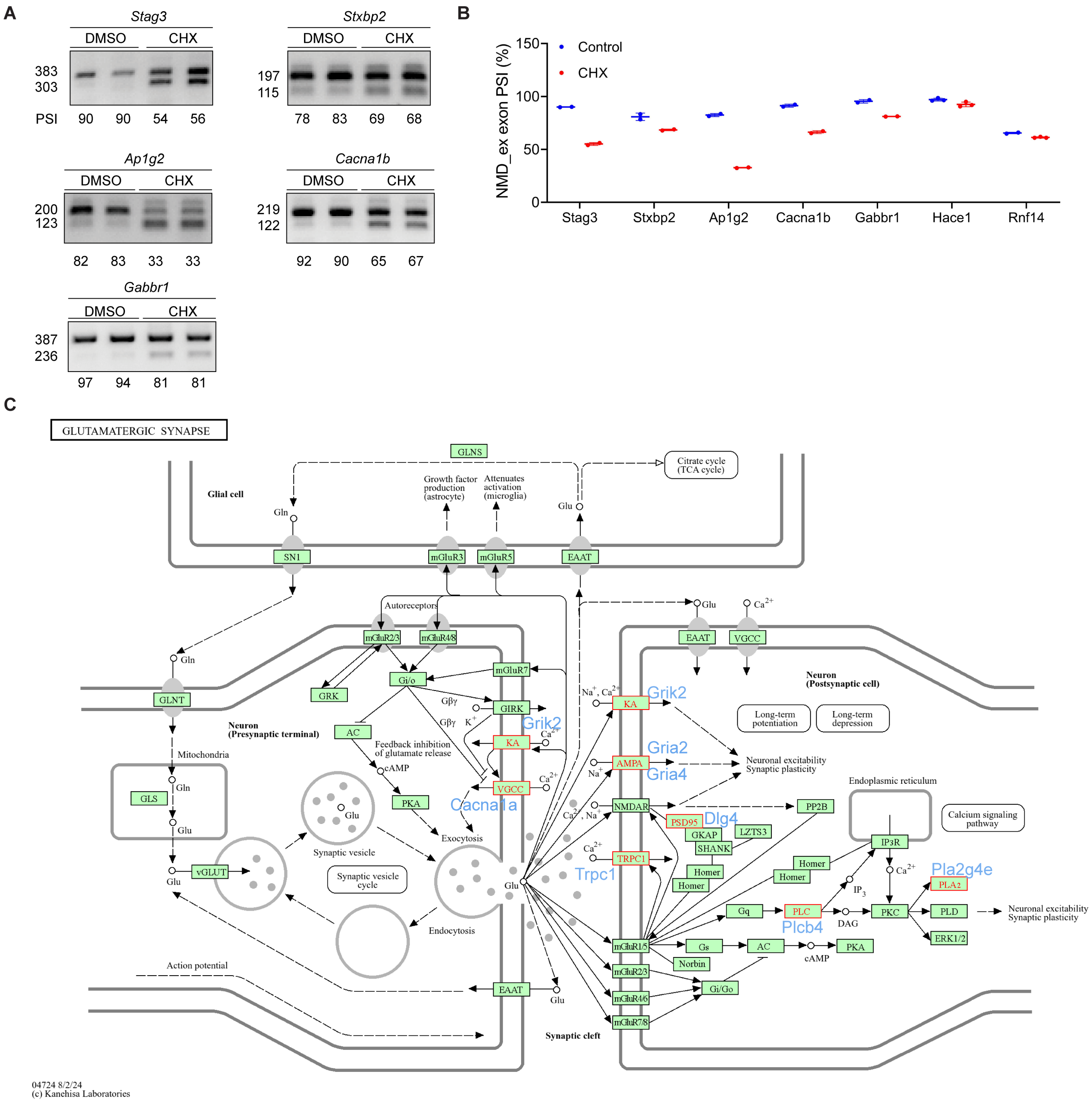

Supplemental Figure 6. Validation of SE-NMD_ex events in the developing mouse brain.

(**A** and **B**) RT-PCR validation of NMD_ex events in primary mouse cortical neurons treated with CHX (**A**). All comparisons are statistically significant (**B**, $p < 0.05$, t-test, $n = 2$ or 3 per group).

(**C**) AS-NMD-regulated genes are enriched in the KEGG “Glutamatergic synapse” pathway. Red nodes indicate enriched terms; blue gene symbols indicate GSEA lead genes with decreased NMD_in or increased NMD_ex PSI.

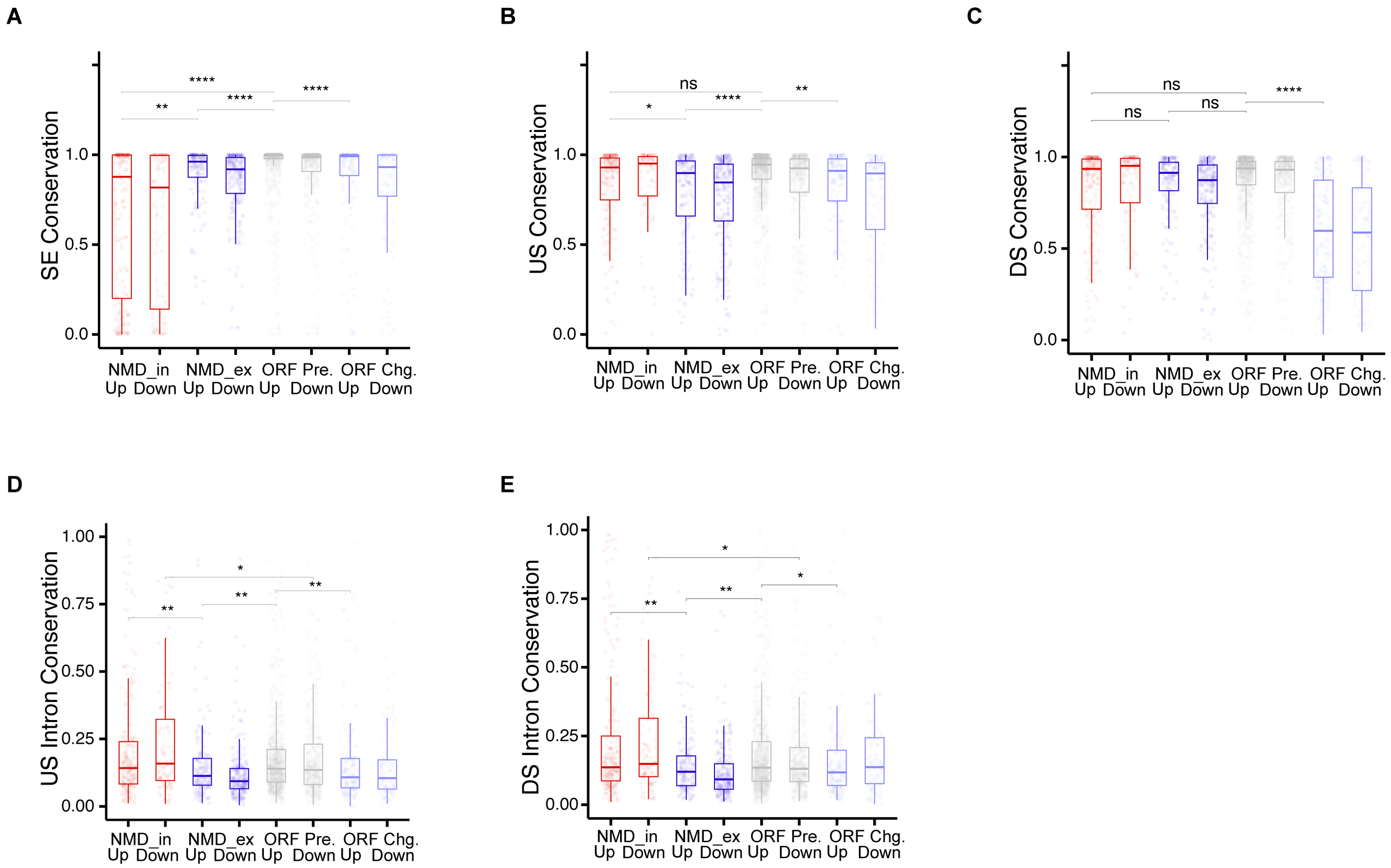

Supplemental Figure 7. Conservation analysis of SEs and their flanking intronic regions.

(**A**–**E**) Comparison of average conservation scores (UCSC phastCons60) across different components of significantly regulated SE events during mouse brain development: SE exon (**A**), upstream exon (**B**), downstream exon (**C**), upstream intron (**D**), and downstream intron (**E**). Groups include NMD_in_Up (*n* = 209, Up represents upregulated during brain development), NMD_in_Down (*n* = 98, Down represents downregulated in brain development), NMD_ex_Up (*n* = 157), NMD_ex_Down (*n* = 231), ORF_Preserving Up (*n* = 928), ORF_Preserving Down (*n* = 456), ORF_Changing Up (*n* = 150), and ORF_Changing Down (*n* = 92). Statistical significance was evaluated using the Wilcoxon rank-sum test. ($p \leq 0.05$: *, $p \leq 0.01$: **, $p \leq 0.001$: ***, $p \leq 0.0001$: ****)

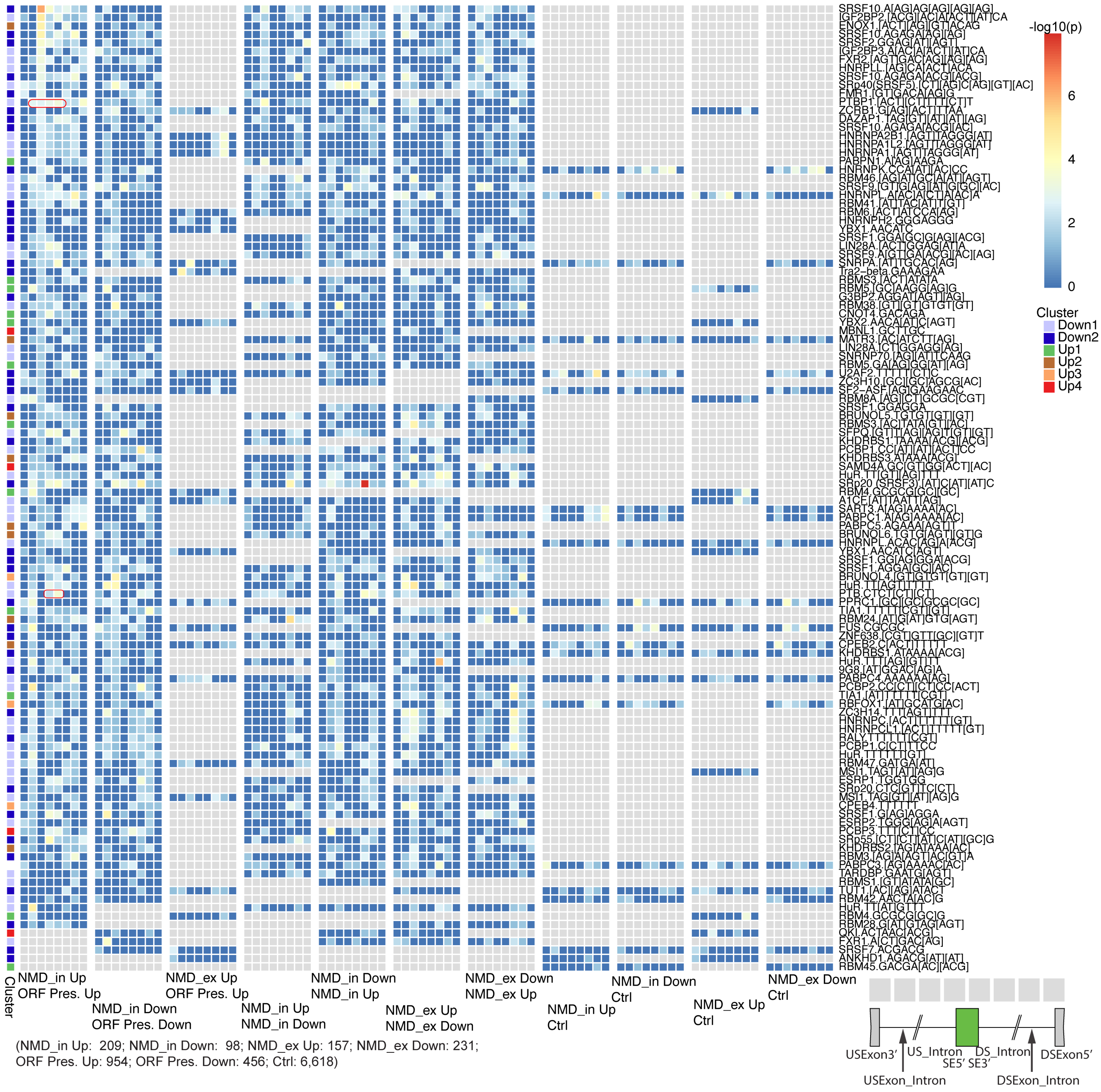

Supplemental Figure 8. RBP motif distributions for AS-NMD skipped exons and flanking introns. The heatmap displays the $-\log_{10}(p)$ values for RBP motif enrichment across eight defined regions surrounding SEs, including the 3' 50 nt of the upstream exon (USExon3'), the 250 nt intronic region immediately downstream of the upstream exon (USExon_intron), the 250 nt upstream intron of the skipped exon (US_intron), the 5' 50 nt (SE5') and 3' 50 nt (SE3') of the skipped exon, the 250 nt downstream intron (DS_intron), the 250 nt intron upstream of the downstream exon (DSExon_intron), and the 5' 50 nt of the downstream exon (DSExon5'). Higher $-\log_{10}(p)$ values indicate stronger motif enrichment, with $p > 0.05$ values replaced by 1 ($-\log_{10}(p) = 0$, shown in dark blue), and gray indicating non-significance. The top and bottom x-axis labels for each panel represent foreground and background groups, respectively. Control SE events were filtered using the following criteria: FDR > 0.5, MaxIncLevel > 0.15, and MinIncLevel < 0.85 from the E18.5-E11.5 splicing comparison.

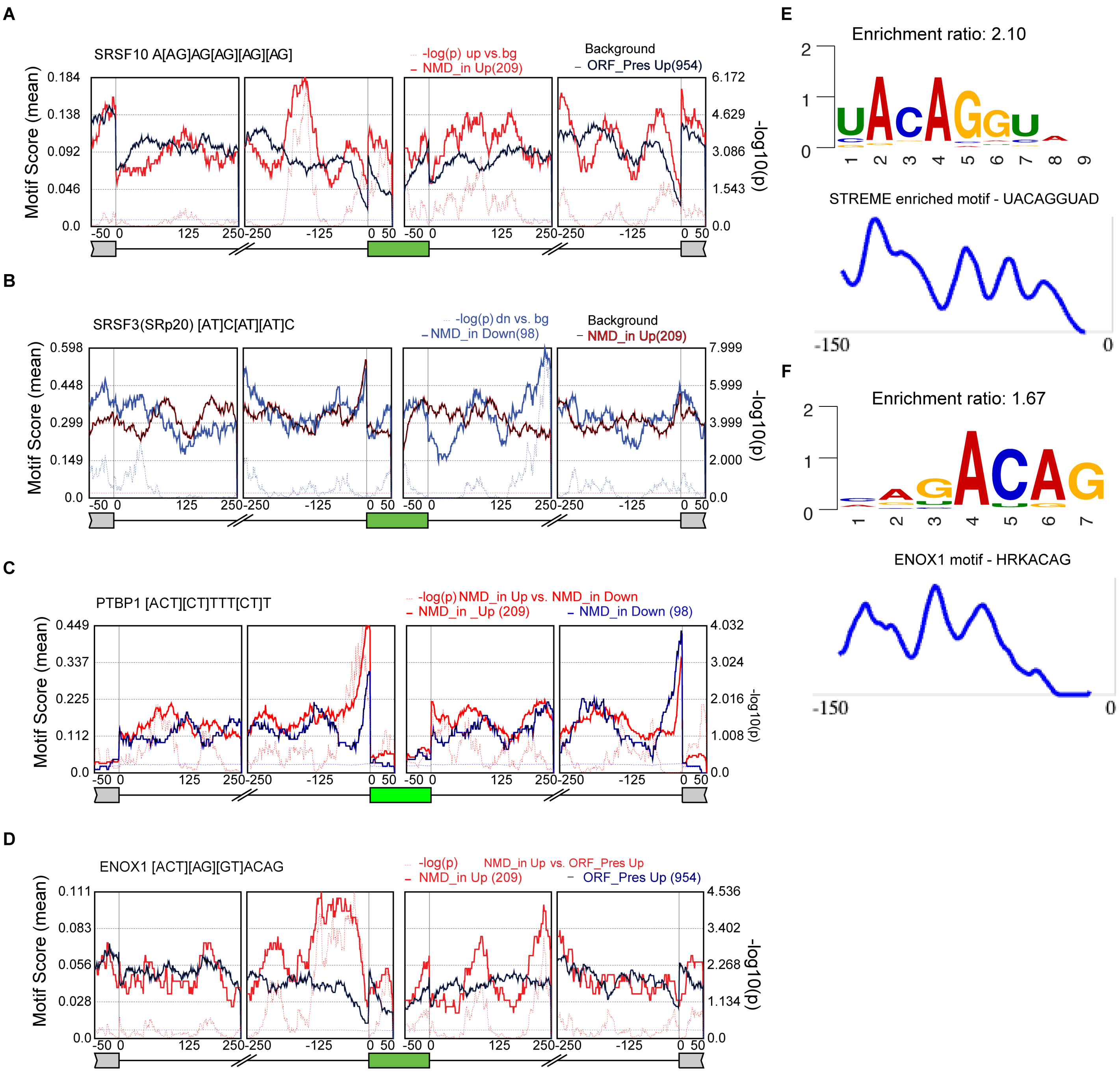

Supplemental Figure 9. Enrichment of selected RBP binding motifs.

(**A**) SRSF10 motifs were significantly enriched in the upstream introns of developmentally upregulated NMD_in exons compared to upregulated ORF-preserving exons, with additional enrichment observed at the 5' end of the skipped exon.

(**B**) SRSF3 motifs were enriched in the downstream introns of developmentally downregulated NMD_in exons compared to upregulated NMD_in events.

(**C**) The PTBP1 motif differed between developmentally upregulated and downregulated NMD_in exons.

(**D**) The [ACT][AG][GT]ACAG motif was enriched in the −125 nt to −50 nt upstream intron of the developmentally upregulated NMD_in exons when compared with upregulated ORF-preserving exons.

(**E** and **F**) STREME analysis identified an enriched motif from the comparison between upregulated NMD_in and ORF-preserving events (**E**), which shares a similar distribution and core "ACAG" sequence (**F**).

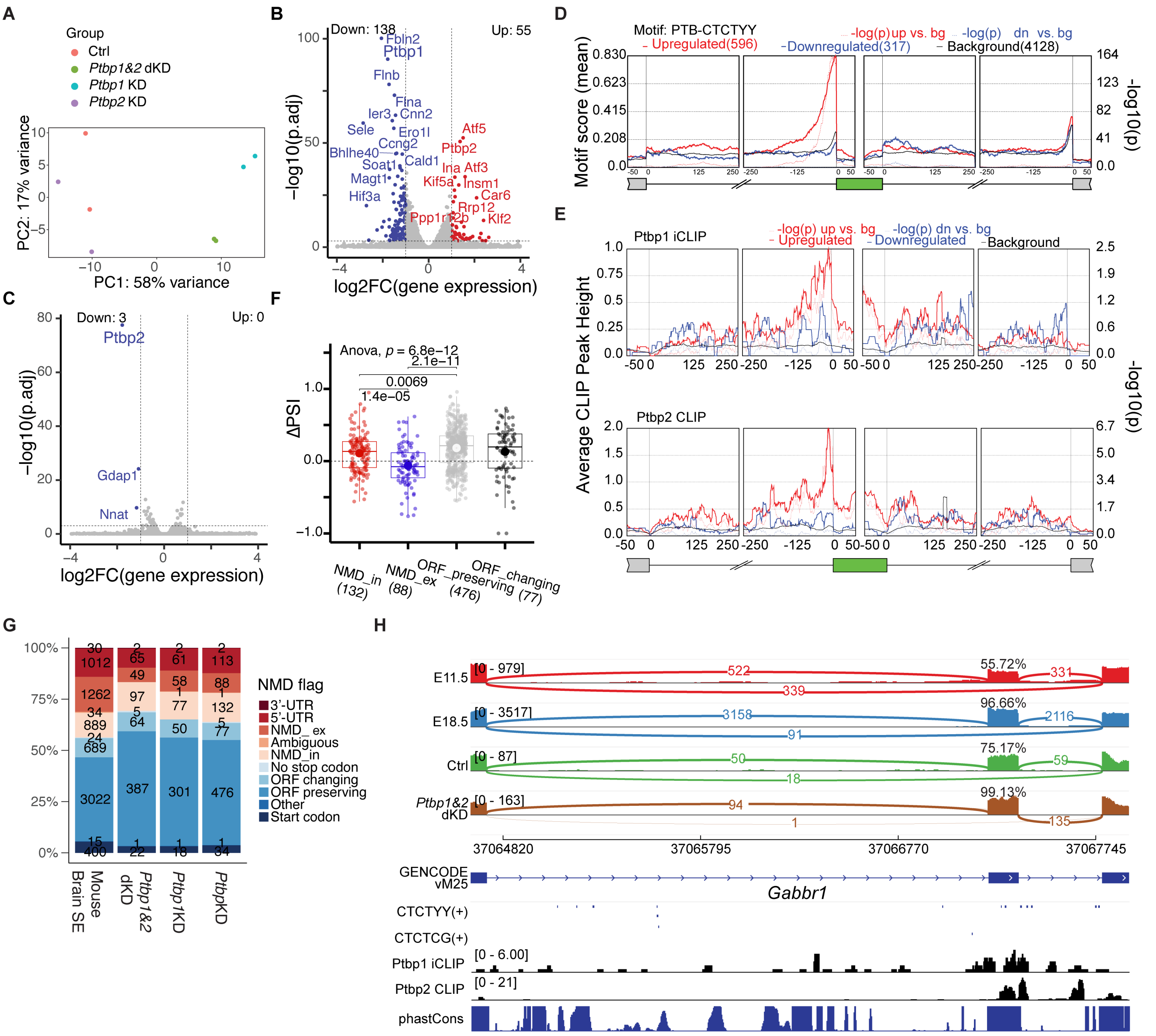

Supplemental Figure 10. Ptbp1/2 directly regulate a subset of AS-NMD exons.

(A) PCA of gene expression profiles showing separation between control, *Ptbp1* KD, and *Ptbp1/2* double KD (dKD) samples (2 biological replicates for each of the 4 groups).

(**B** and **C**) Volcano plots highlight differentially expressed genes (DEGs) upon *Ptbp1* KD (**B**) and *Ptbp2* KD (**C**) (|$\log_2$FC| ≥ 1 and adjusted $p < 0.001$).

(**D**) The CTCTYY motif was significantly enriched in upstream intronic regions of differentially spliced SEs following *Ptbp1/2* dKD.

(E) CLIP-seq data showing that Ptbp1 and Ptbp2 bind to differentially spliced SEs in *Ptbp1* KD.

(**F**) PSI changes upon *Upf1* KD in Neuro2a cells (y-axis) for exons that were differentially spliced in *Ptbp1/2* dKD. NMD_in ($n = 132$, mean: 0.11, median: 0.13), ORF preserving ($n = 476$, mean: 0.18, median: 0.21), ORF changing ($n = 77$, mean: 0.13, median: 0.20), and NMD_ex ($n = 88$, mean: −0.06, median: −0.08). The mean is marked by the middle dot (Anova and t-test).

(**G**) Distribution of NMD flags for differentially spliced SEs in *Ptbp1* and/or *Ptbp2* KD, along with developmentally regulated SEs in the mouse brain.

(**H**) An example of the NMD_ex *Gabbr1* exon 14|15 skipped event (chr17:37067213-37067364), which exhibited increased PSI after *Ptbp1/2* dKD.

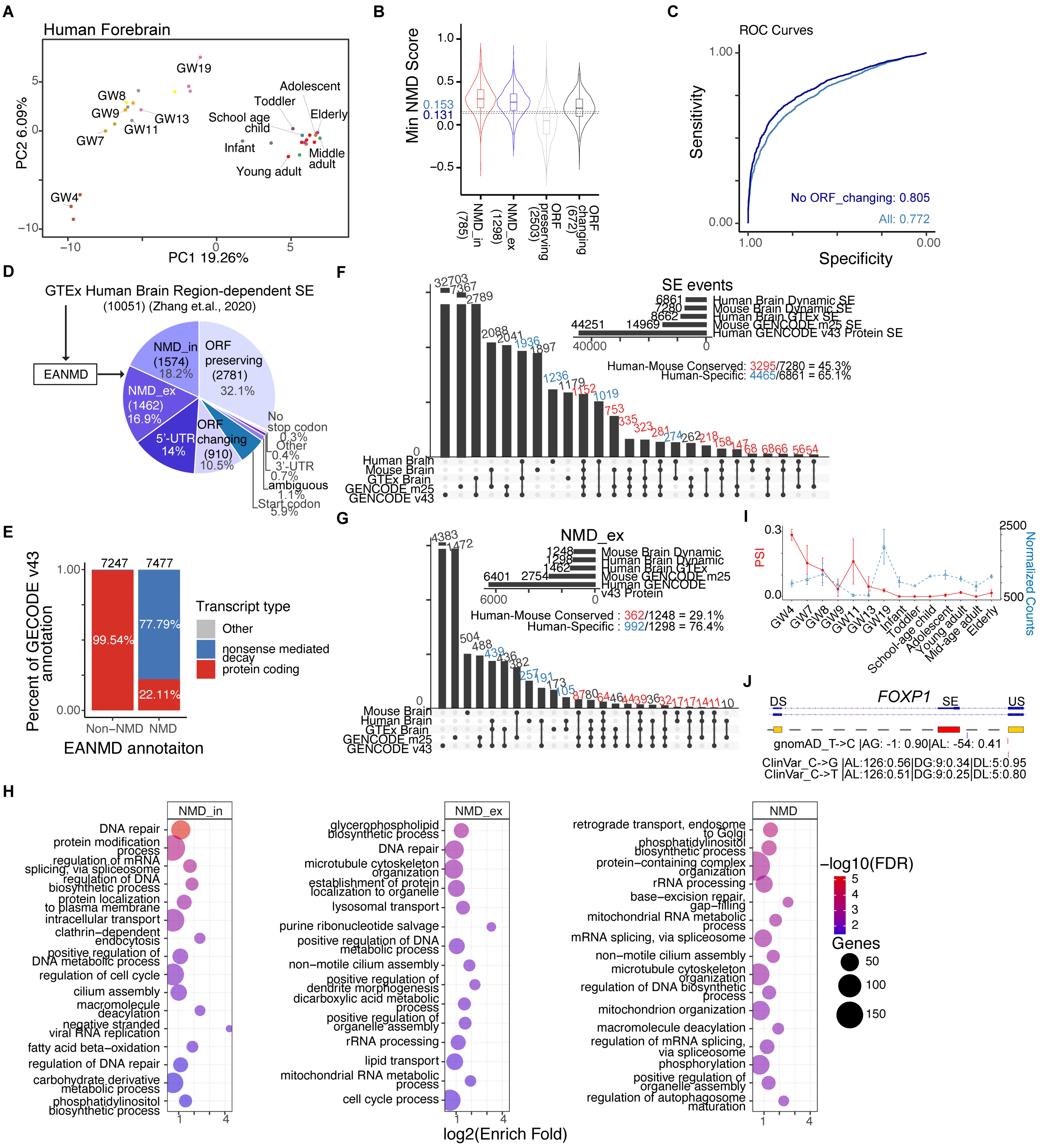

Supplemental Figure 11. AS-NMD in the human brain.

(**A**) PCA of human forebrain samples based on PSI of dynamic SE events (same numbers of samples as shown in Figure 4A).

(**B**) Boxplot of EANMD-predicted NMD scores in the human forebrain.

(**C**) ROC curves for NMD vs. non-NMD classification using optimized NMD score thresholds. The navy line excludes “NMD changing” events (0.131, AUC: 0.805), while the blue line includes all SEs (0.153, AUC: 0.772).

(**D**) EANMD prediction for human brain region dependent SEs (GTEx v7).

(**E**) Barplot showing GENCODE annotations for genes classified by EANMD annotations (SE-NMD).

(**F**) UpSet plot showing overlaps of SE events across developing human and mouse brains.
(**G**) Intersections between mouse and human brain NMD_ex SEs.
(**H**) Enriched Biological Processes of human-specific SE-NMD genes.
(**I**) Expression of *FOXP1* (blue line) is inversely correlated with PSI values (red line) of the AS-NMD exon (GRCh38, chr3:70972010–70972180) during brain development.
(**J**) Splicing-impacting variants (gnomAD and ClinVar, predicted via SpliceAI) around the *FOXP1* AS-NMD exon. DG: donor gain. AG: acceptor gain. DL: donor loss. AL: acceptor loss.

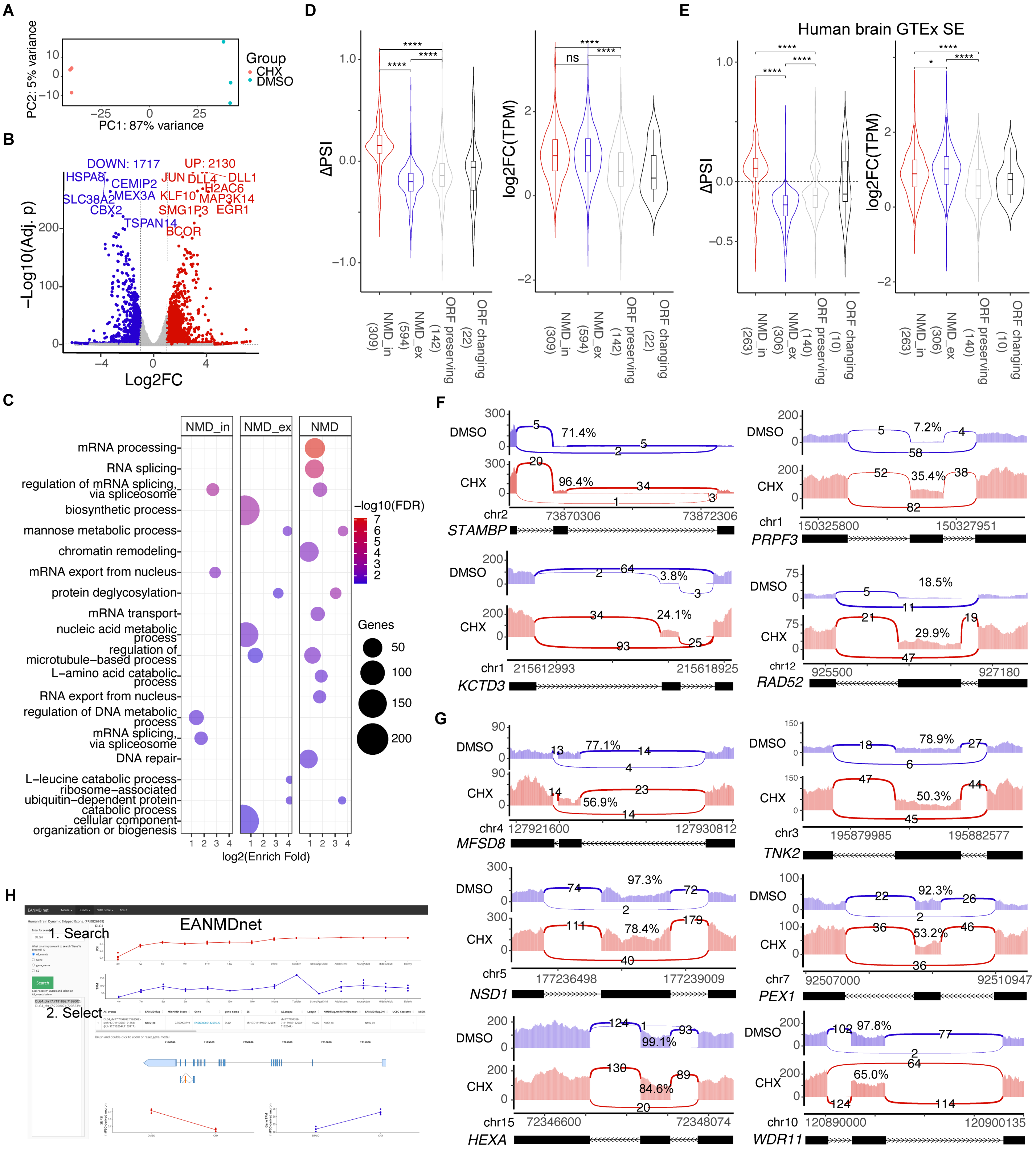

Supplemental Figure 12. iPSC-derived neuron validation of the human brain SE types.

(**A**) PCA of iPSC-derived neuron RNA-seq samples (DMSO and CHX treated, n = 3 biological replicates).

(**B**) Volcano plot of differentially expressed genes in iPSC-derived neurons after CHX treatment.

(**C**) Enriched GO BP terms of AS-NMD genes in iPSC-derived neurons responded to CHX treatment, with all expressed genes as background.

(**D** and **E**) Violin plots showing the PSI (left) and mRNA level changes (right) of AS-NMD exons of iNeuron significant (|ΔPSI| > 0.1, FDR < 0.05, min NMD score > 0.132) SEs (**D,** NMD_in: n = 309, NMD_ex: n = 594, ORF preserving: n = 142, and ORF changing: n = 22) and GTEx SEs with significant (FDR < 0.05, min

NMD score > 0.132) iNeurons SEs (**E,** NMD_in: *n* = 263, NMD_ex: *n* = 306, ORF preserving: *n* = 140, and ORF changing: *n* = 10), one-way ANOVA followed by Tukey's multiple comparisons test, 195 NMD_in and 294 NMD_ex events showed expected changes in CHX-treated iNeurons.

(**F** and **G**) Sashimi plots showing validated NMD_in (**F**) and NMD_ex (**G**) events in iPSC-derived neurons.

(**H**) A snapshot of EANMDnet (https://zlab1.shinyapps.io/EANMDnet), an interactive web portal presenting developmentally regulated AS-NMD events in mouse and human brains.

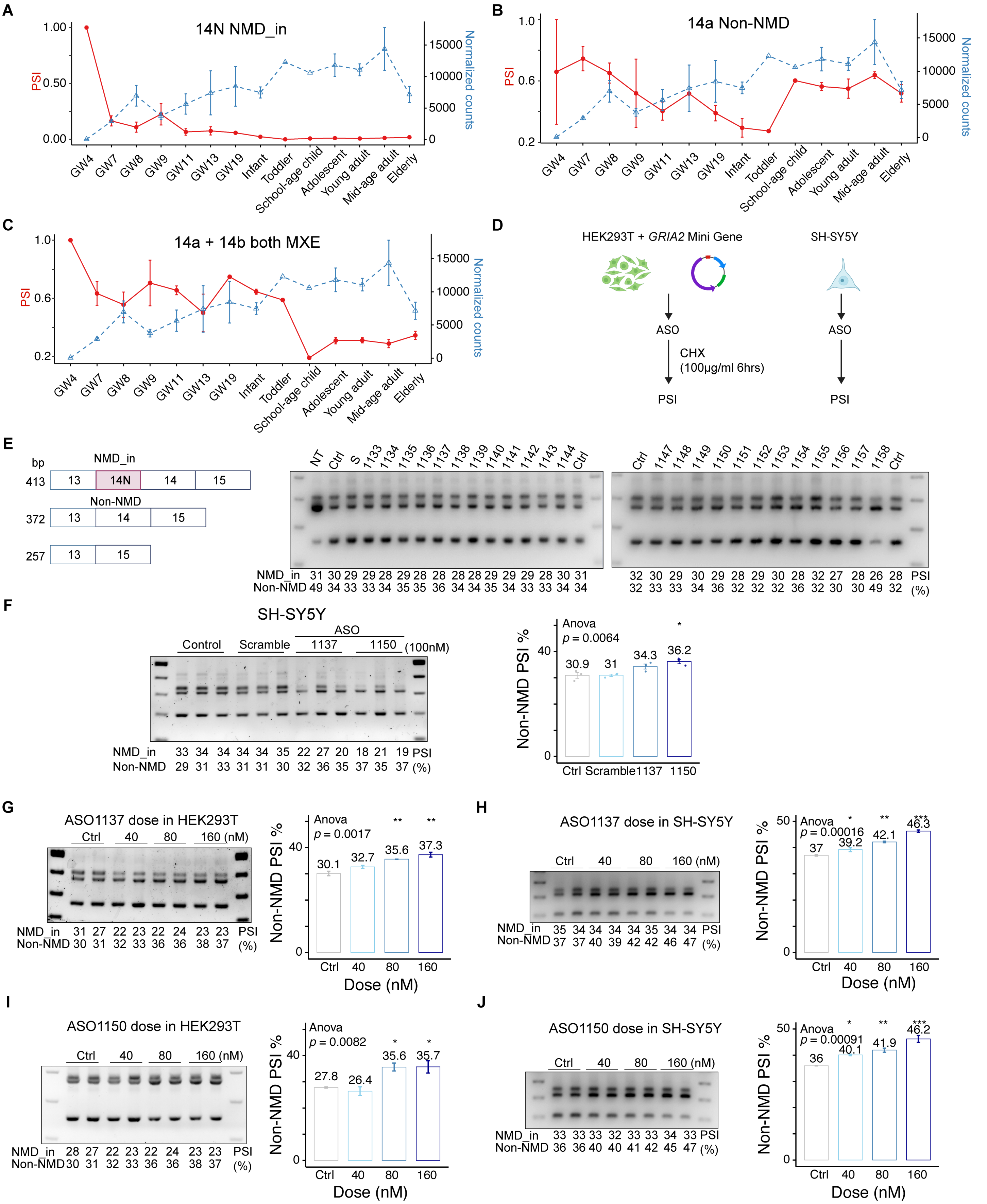

Supplemental Figure 13. Upregulation of *GRIA2* expression by suppressing the AS-NMD exon 14N with ASOs.

(**A**) PSI and normalized expression of *GRIA2* exon 14N (NMD_in) across human brain development.

(**B**) PSI and normalized expression trajectories of *GRIA2* exon 14a (ORF_preserving) event.
(**C**) PSI and normalized expression of *GRIA2* transcripts carrying both exons 14a and 14b.
(**D**) Illustration of ASO screens in HEK293T-*GRIA2* stable cells and the SH-SY5Y cell line.
(**E**) Illustration of the NMD_in, non-NMD, and NMD_ex isoforms for *GRIA2*, and gel electrophoresis results for the ASO screens. Bottom numbers showing the PSI values. S for Scramble.
(**F**) RT-PCR validation and quantification of ASO1137 and ASO1150 in SH-SY5Y cells with three biological replicates. Left: DNA gel; Right: Non-NMD PSI (Data represent mean ± SE, t-test n = 3 per group and ANOVA).
(**G** and **H**) Dose-dependent increase of *GRIA2* non-NMD isoform expression in HEK293T-GRIA2 cells (**G**) and in SH-SY5Y cells (**H**) by ASO1137. Left: DNA gel of RT-PCR products; Right: PSI quantification (Data represent mean ± SE, t-test n = 2 per group and ANOVA).
(**I** and **J**) Dose-dependent increase of the *GRIA2* non-NMD isoform in HEK293T-GRIA2 (**I**) and in SH-SY5Y cells (**J**) by ASO1150. Left: DNA gel; Right: PSI quantification (Data represent mean ± SE, t-test n = 2 per group and ANOVA).

References

1. Lin L, Zhao J, Kubota N, Li Z, Lam YL, Nguyen LP, et al. Epistatic interactions between NMD and TRP53 control progenitor cell maintenance and brain size. *Neuron.* 2024.
