# Supplementary material for: Alternative splicing–triggered mRNA decay informs splice-switching targets for neurodevelopmental disorders
Source: J Clin Invest. 2026 Feb 12;136(8):e197271. doi: 10.1172/JCI197271 (PMC13078869; doi:10.1172/JCI197271)

Full unedited gels for Figures 2F, 2G, S5G, S6A, 6D, S13E, S13F, S13G, S13H, S13I and S13J
(Bands in blue rectangles were showed in the figures)

Figure 2F

Gad1

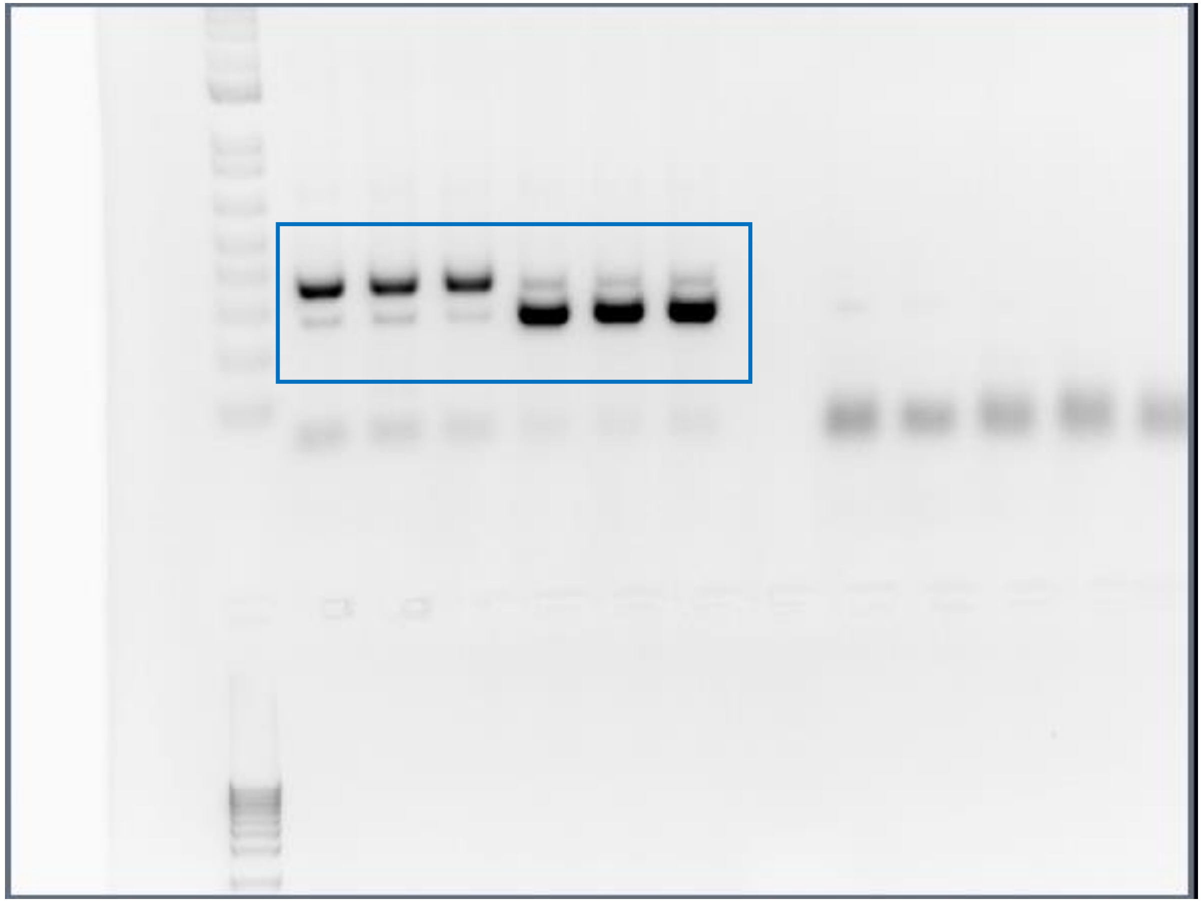

Figure 2F

Gria2

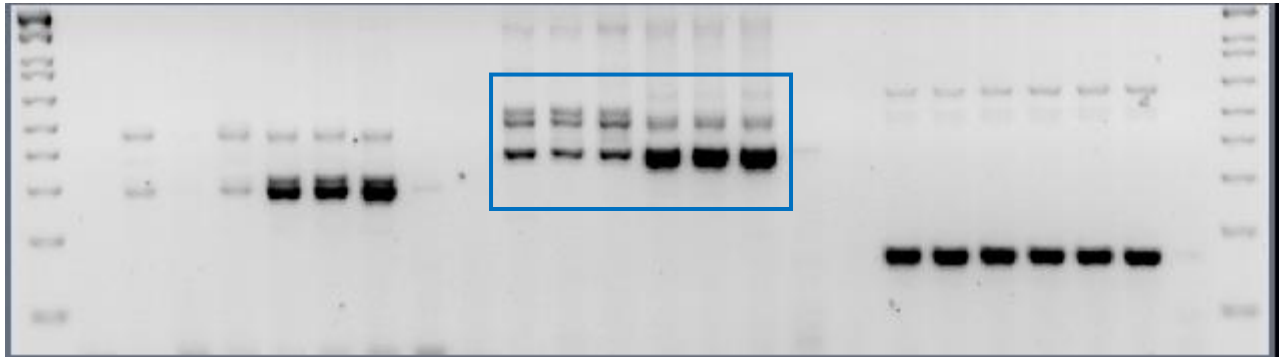

Figure 2F

Gria4

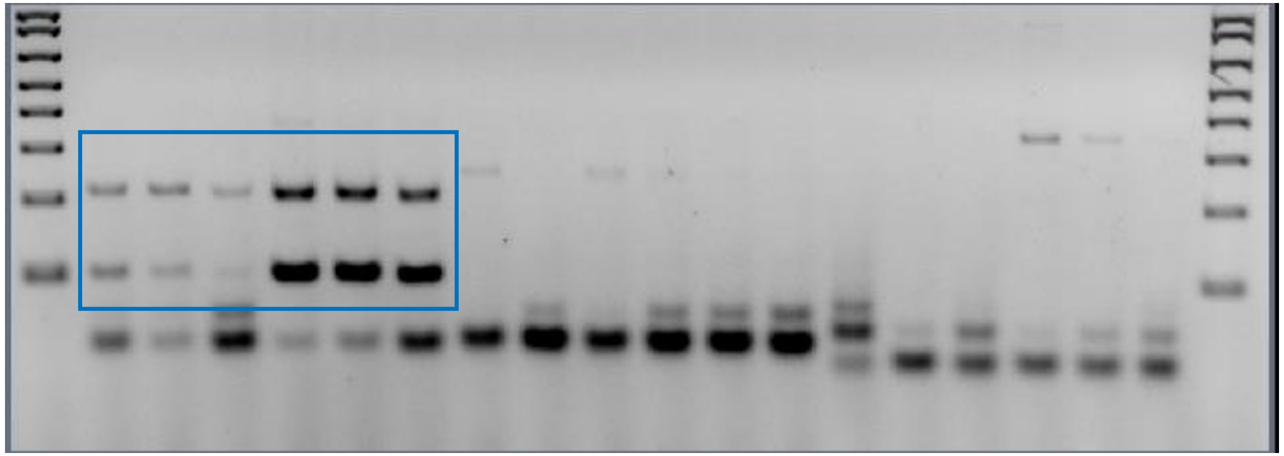

Figure 2F

Oprl1

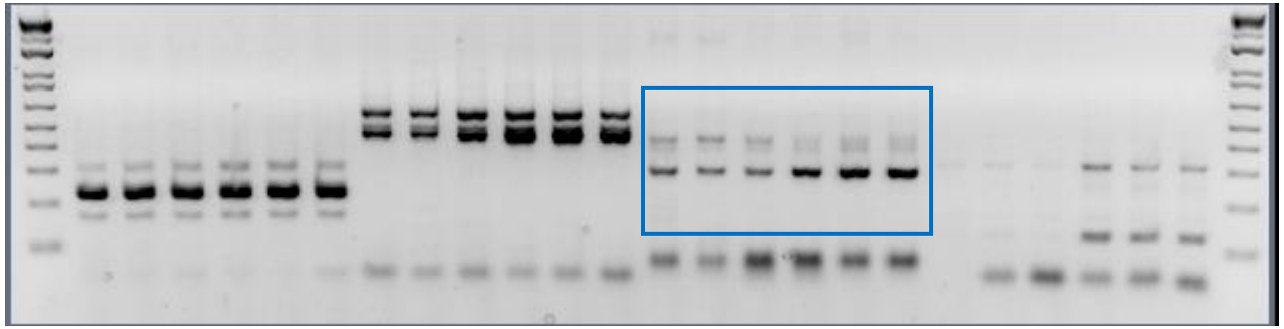

Figure 2F

Eif4a2

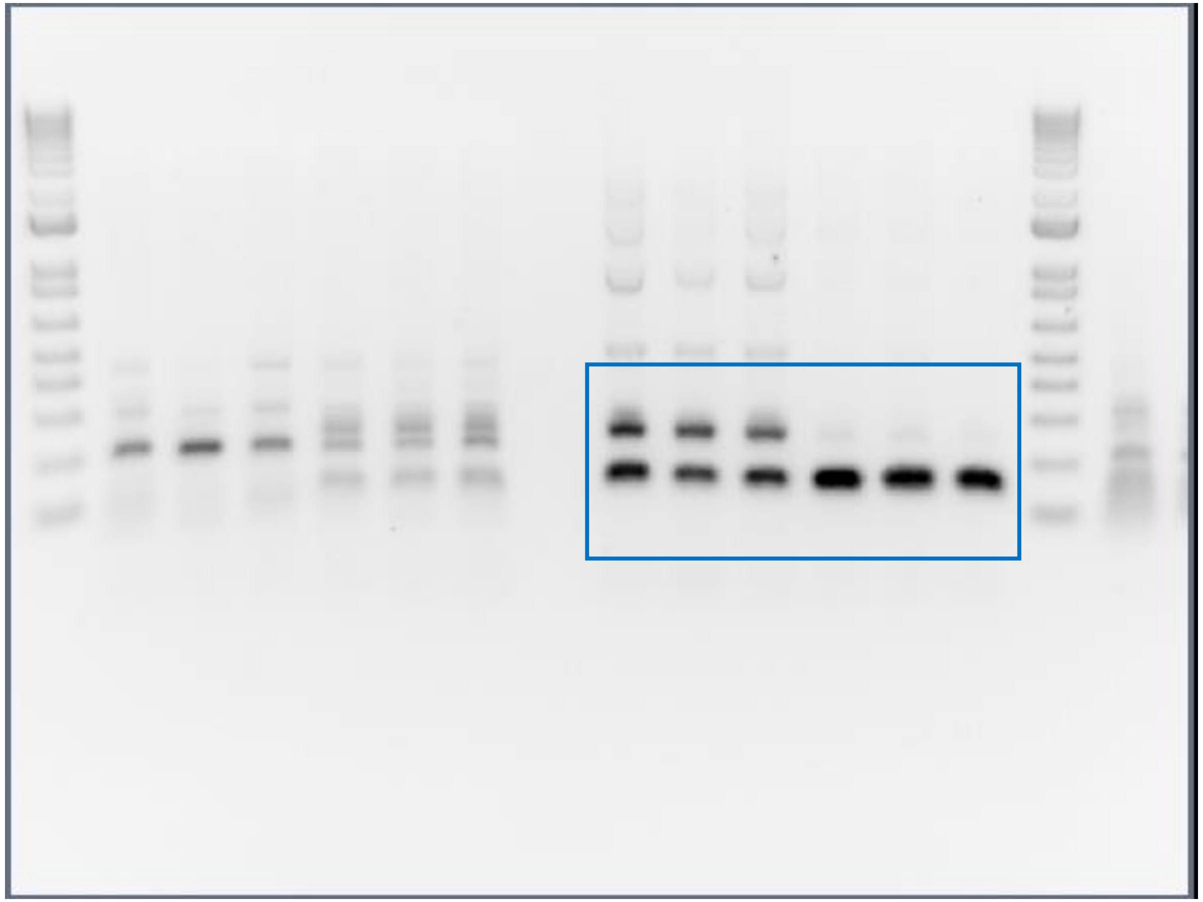

Figure 2F

Taf1b

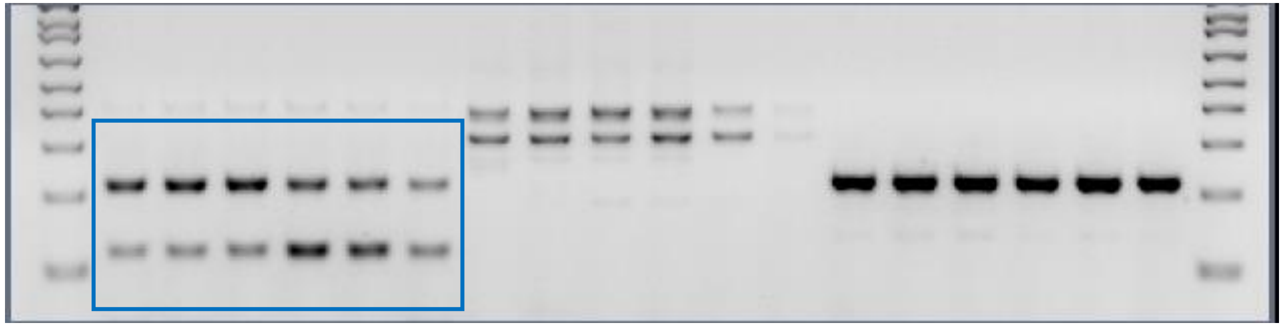

Figure 2F

Iqgap1

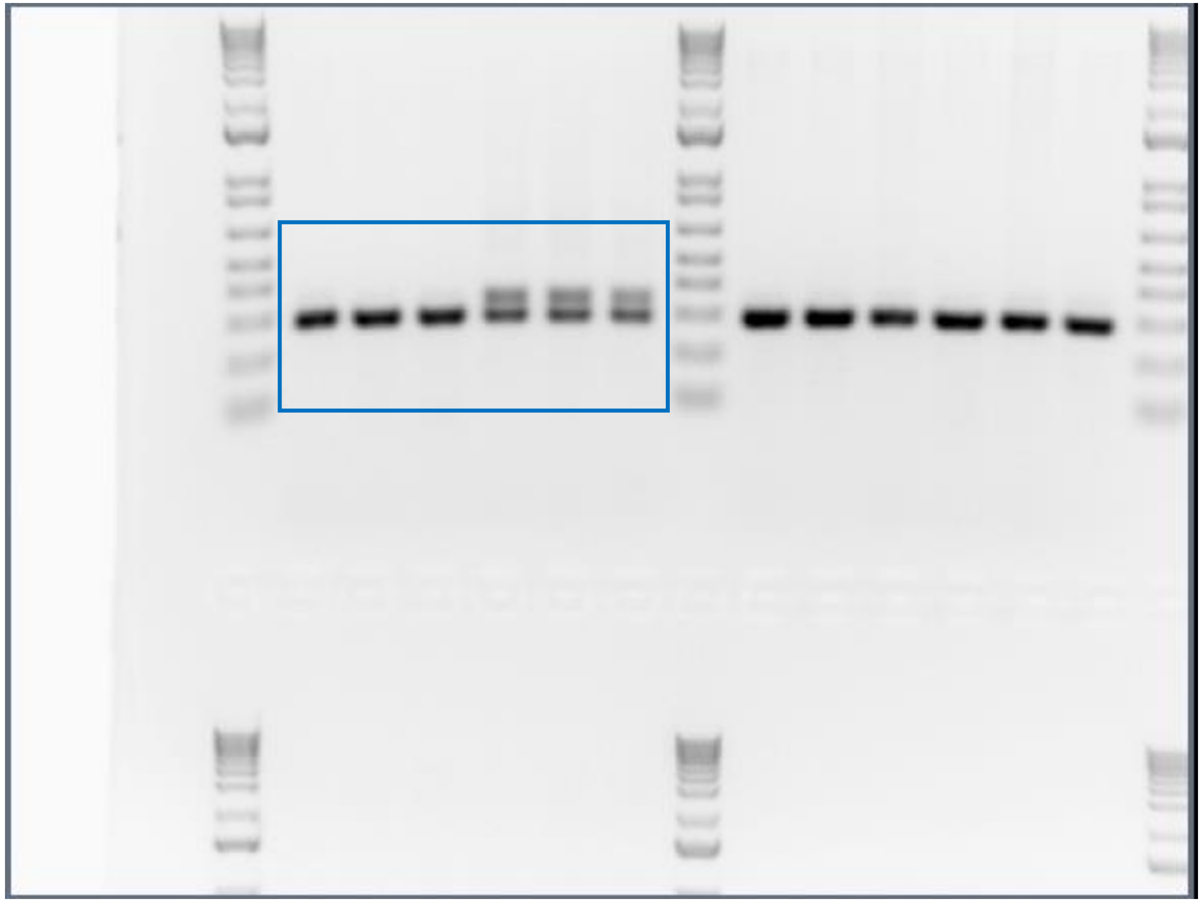

Figure 2F

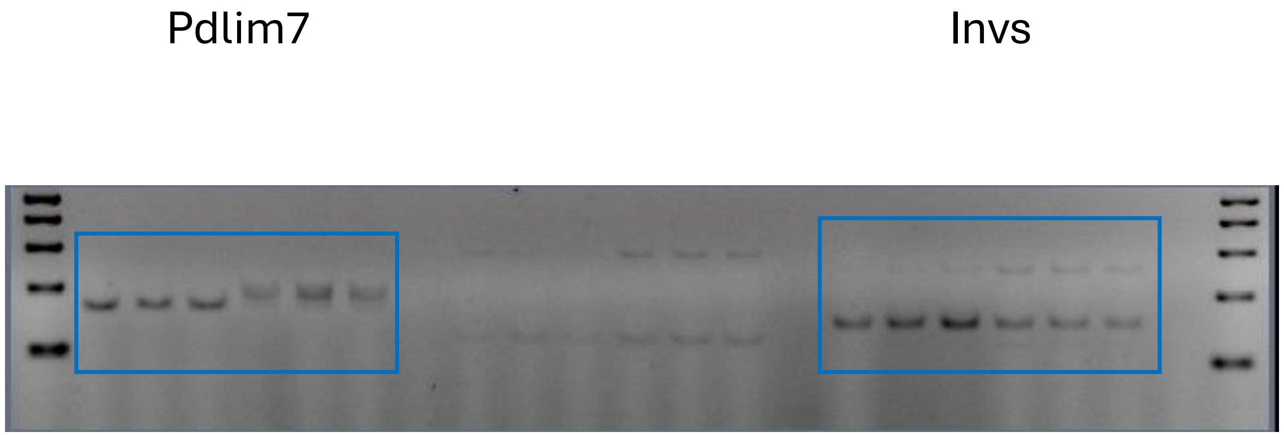

Figure 2F

Plekha7

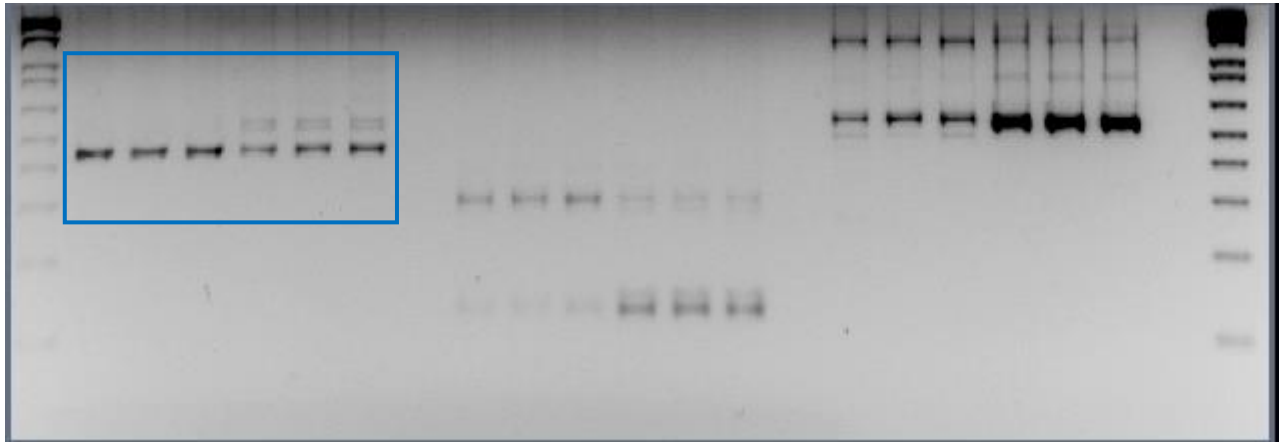

Figure 2F

Rock1

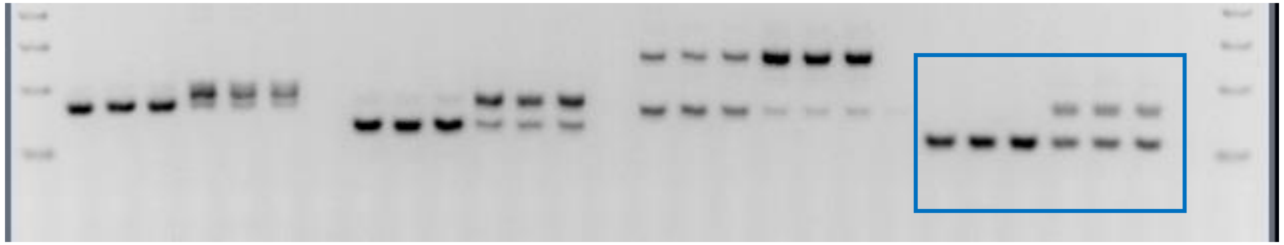

Figure 2F

Hps5

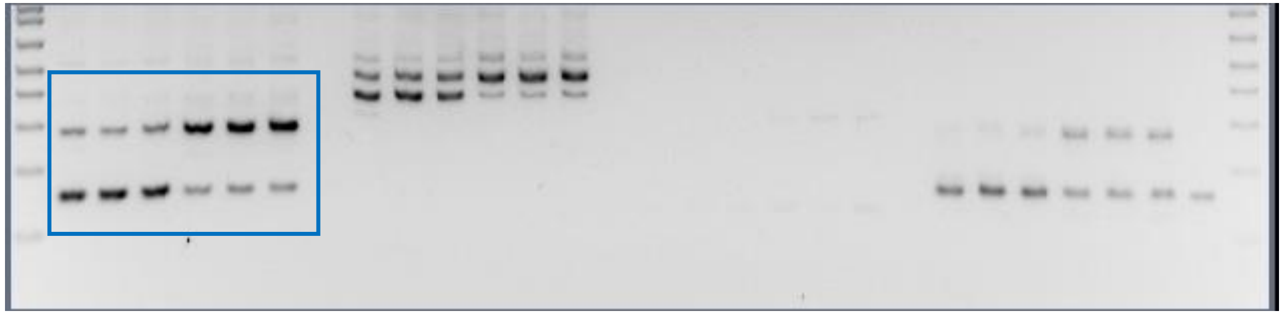

Figure 2G

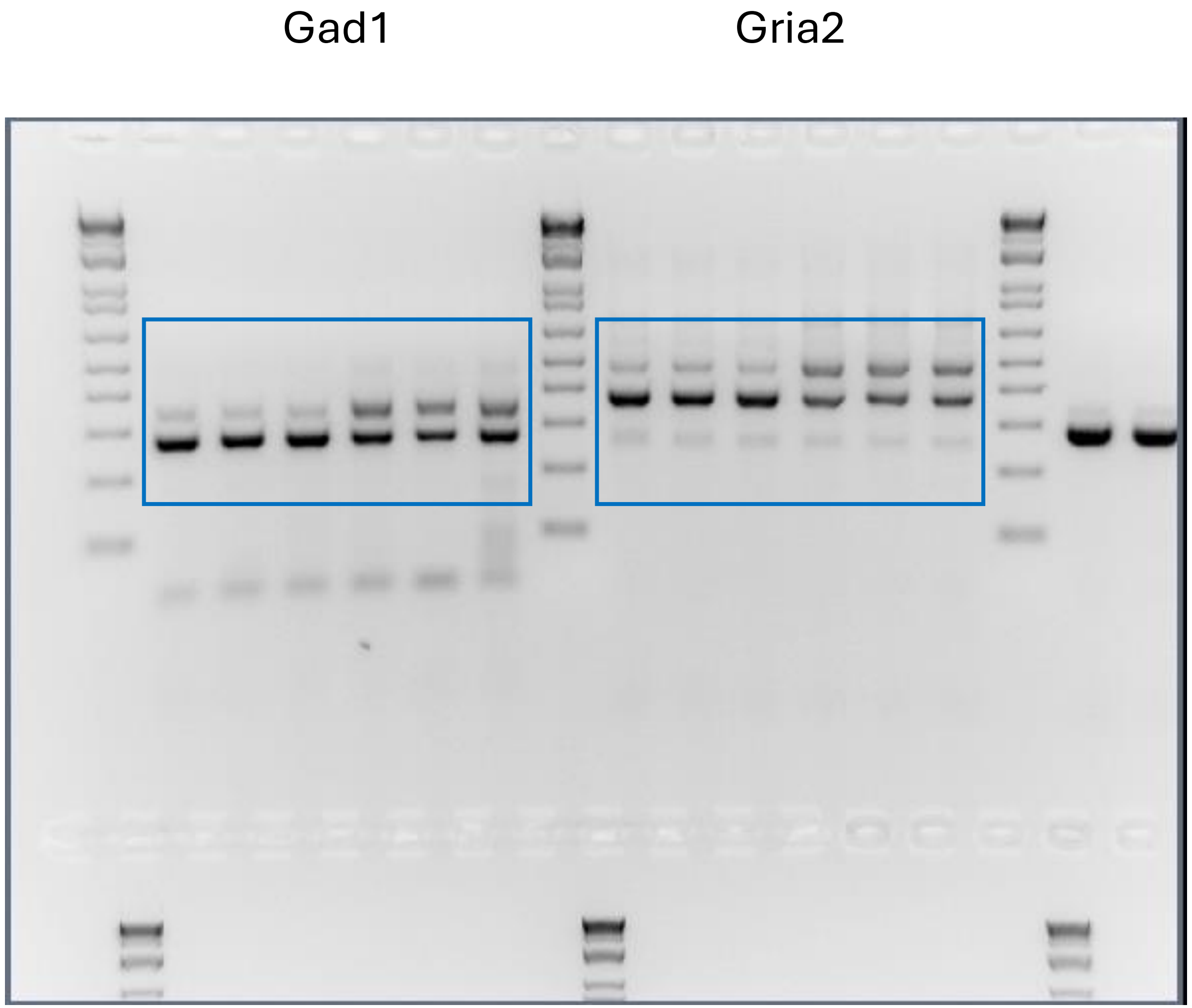

Figure 2G

Gria4

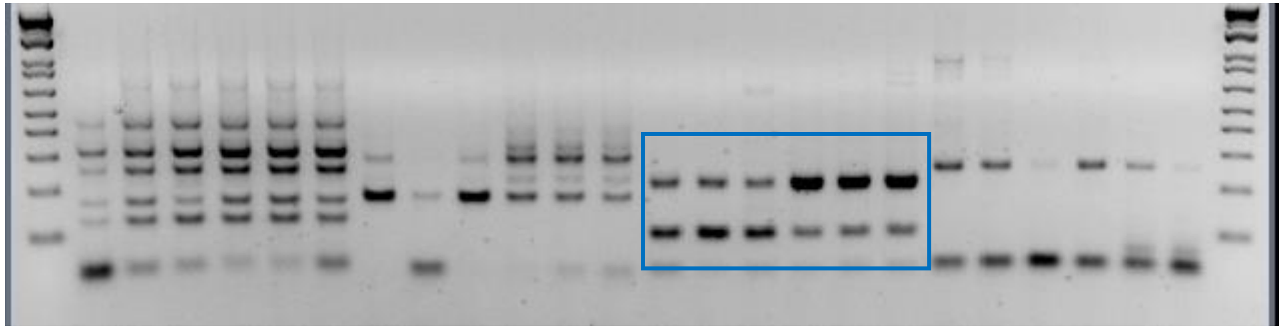

Figure 2G

Oprl1

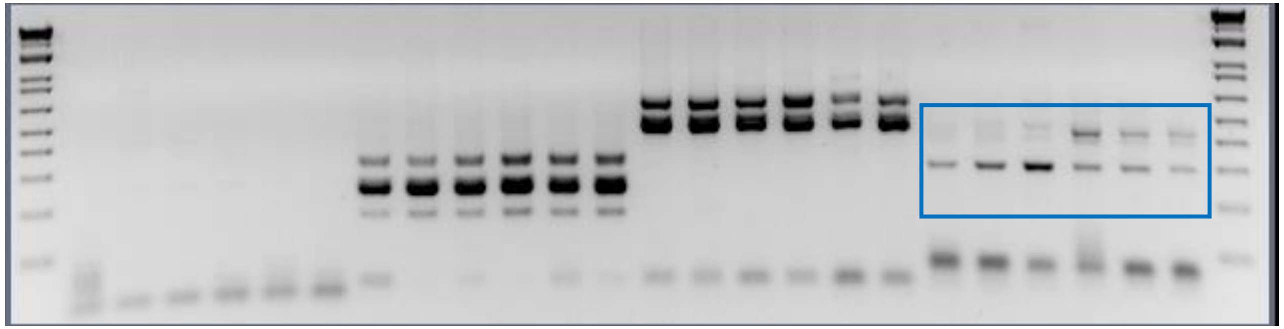

Figure 2G

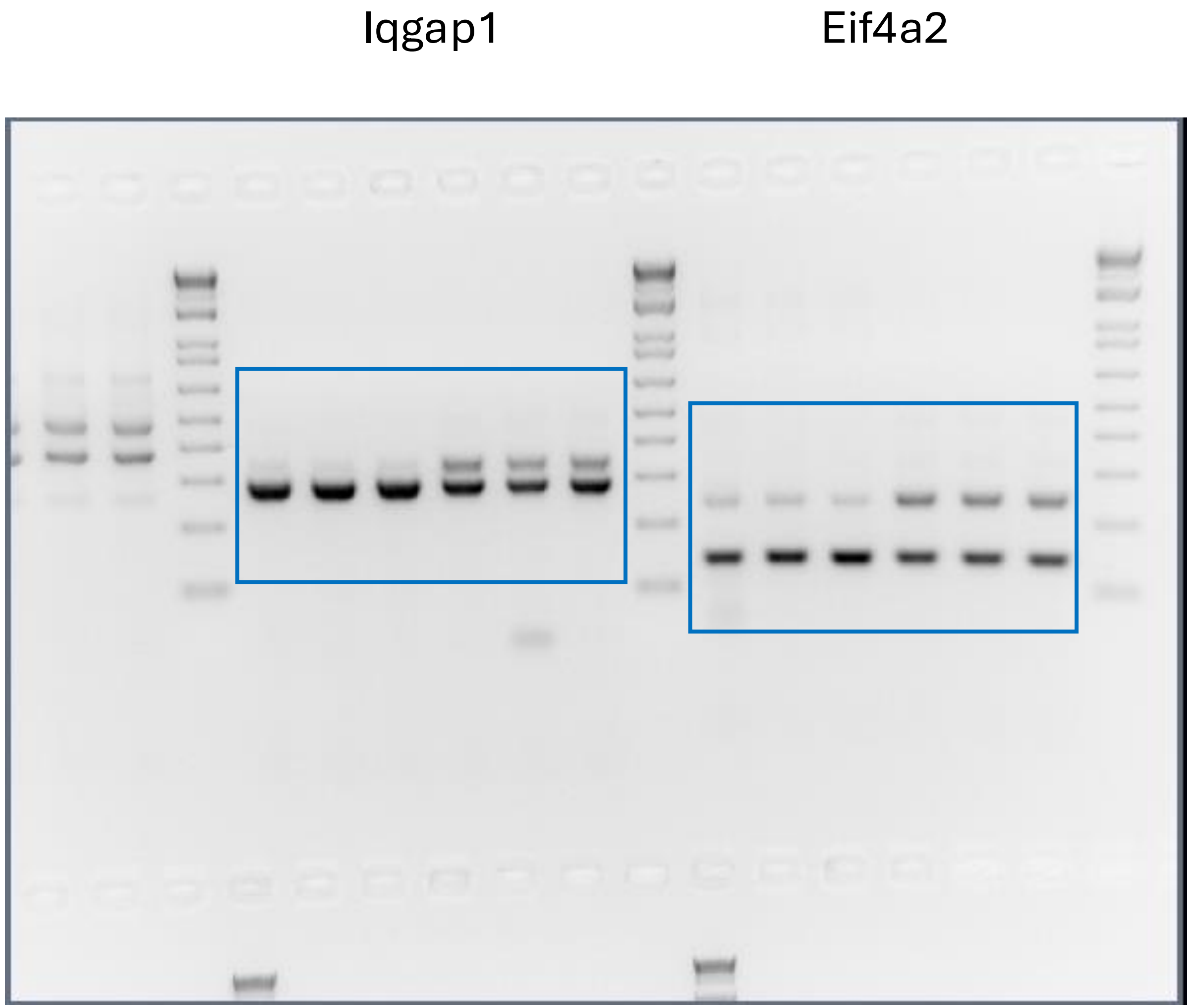

Figure 2G

Taf1b

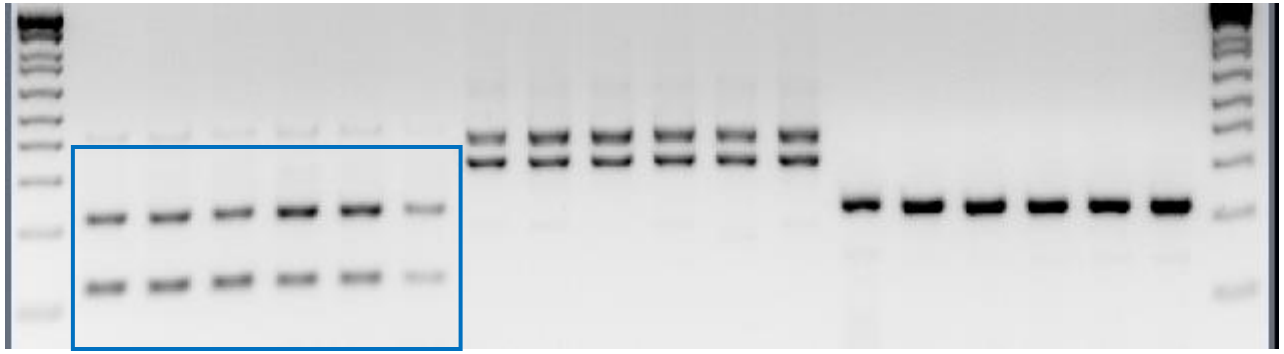

Figure 2G

Snap23

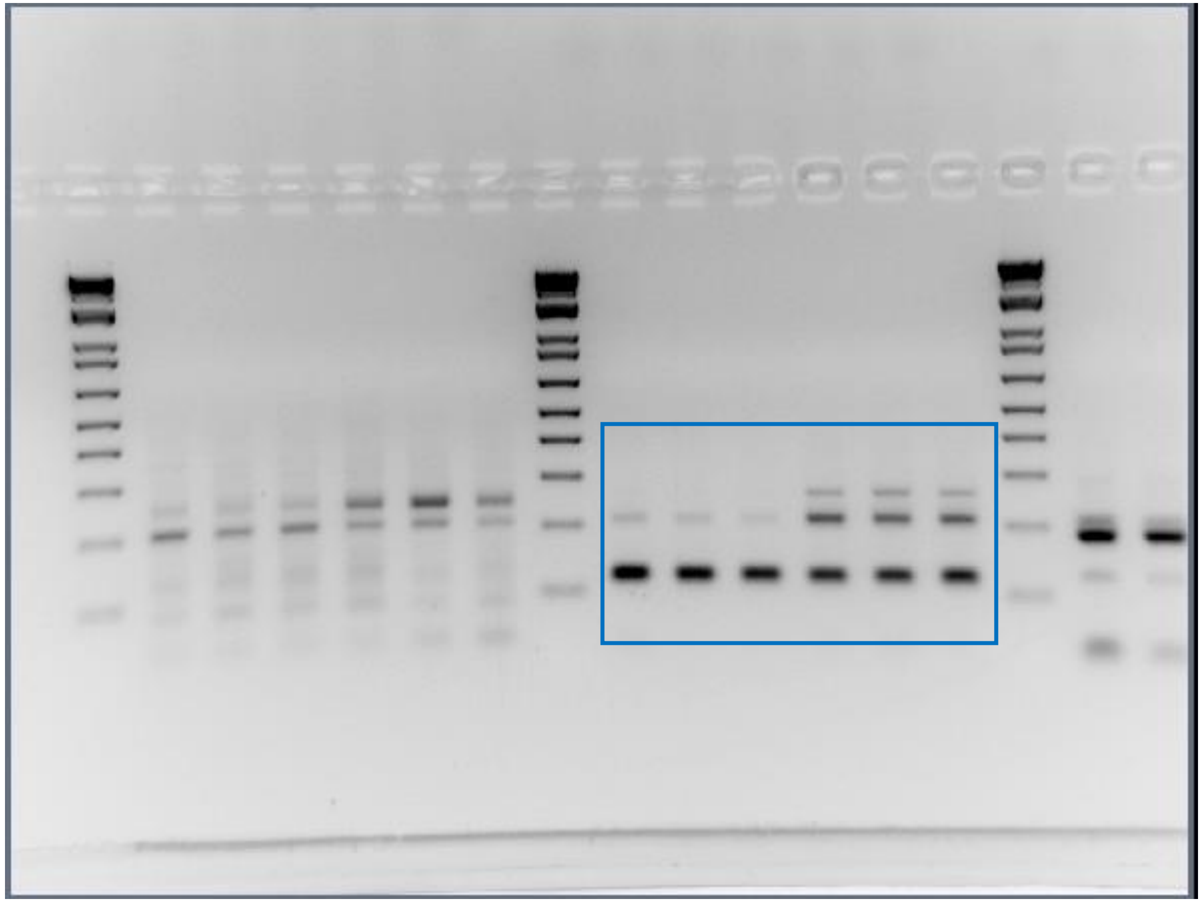

Figure 2G

Hps5

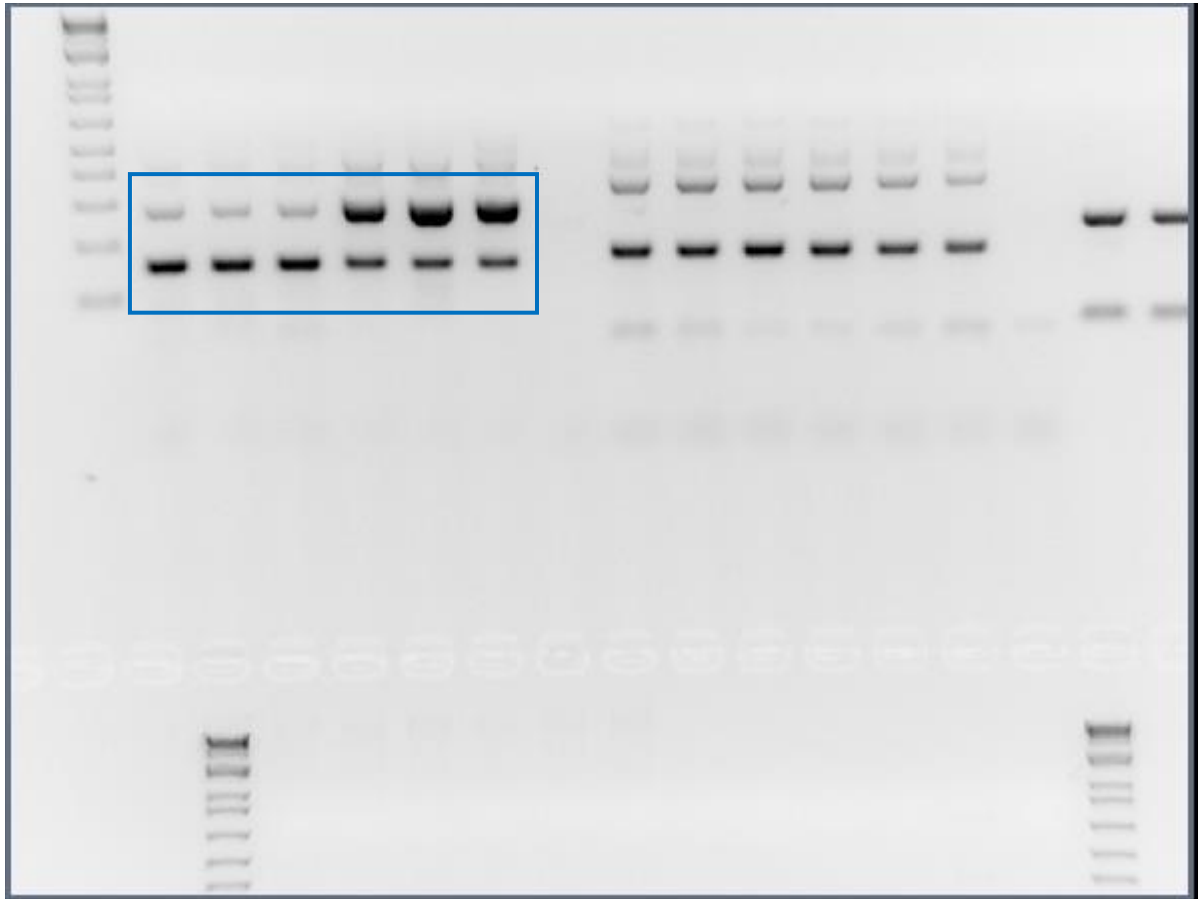

Figure 2G

Pdlim7

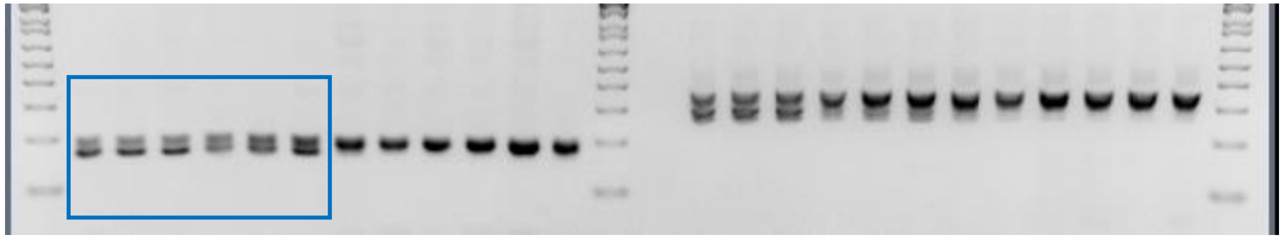

Figure 2G

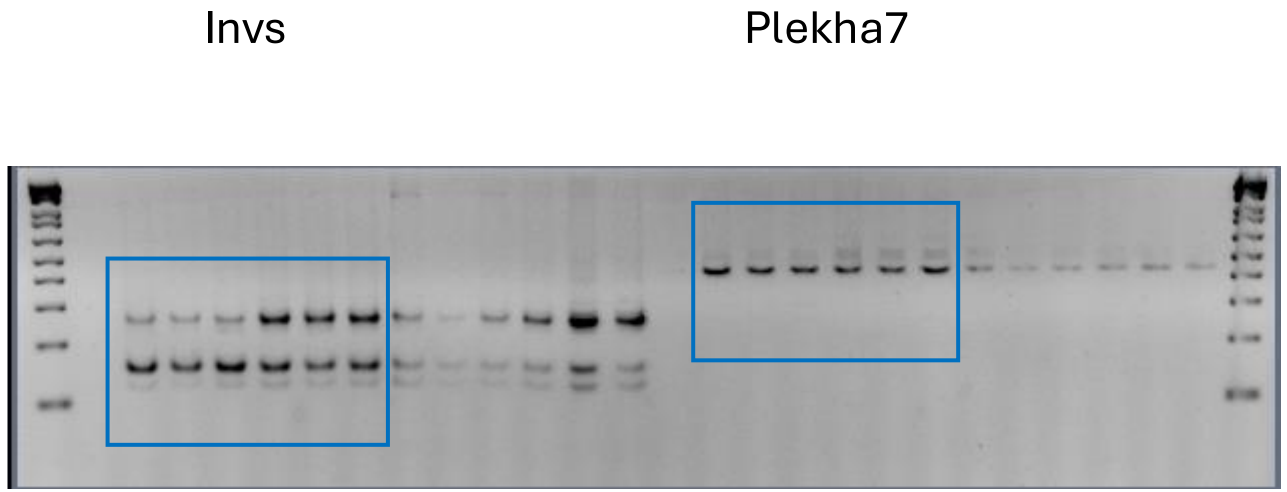

Figure 2G

Rock1

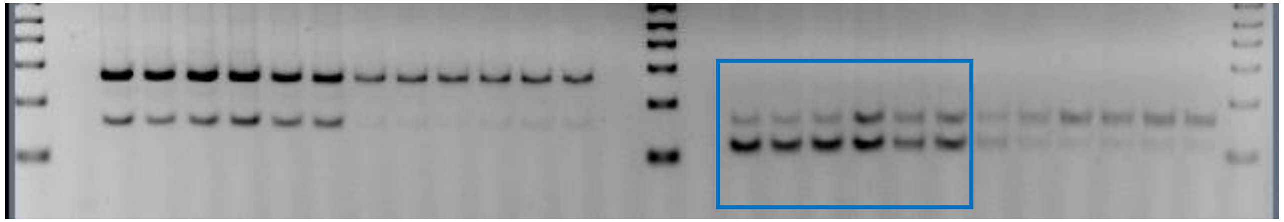

Figure 2G

Alg13

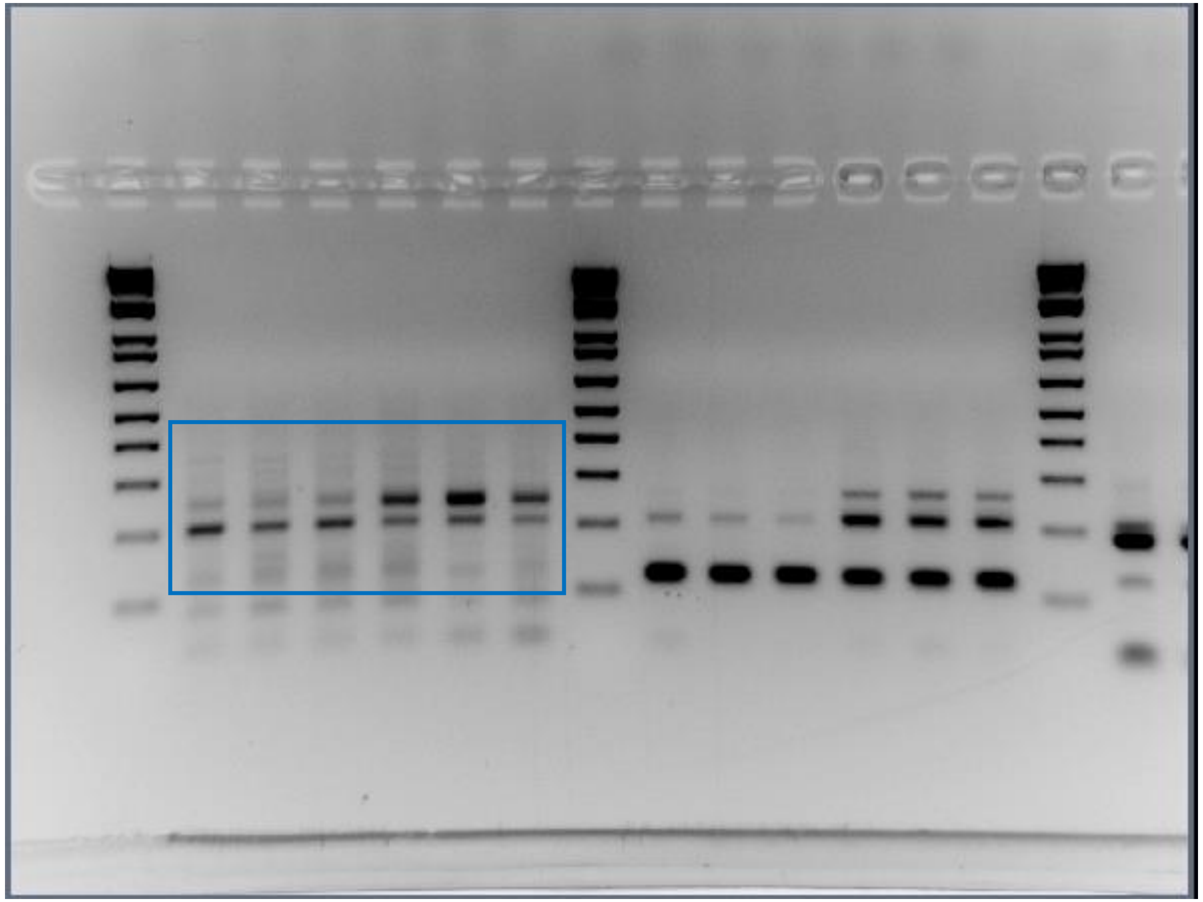

Figure 2G

Ctnnal1

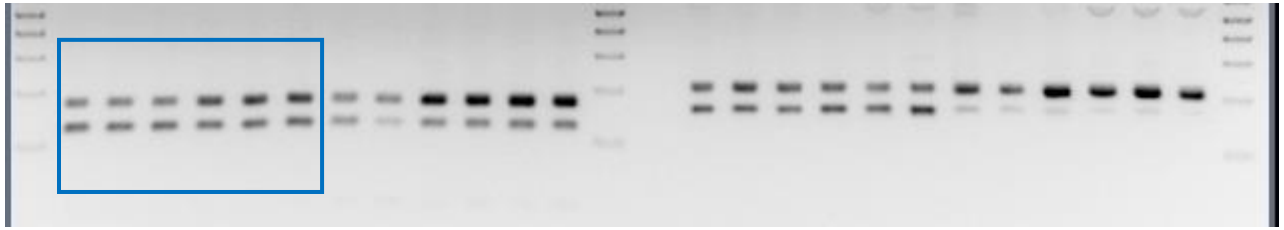

Figure 2G

Ripk2

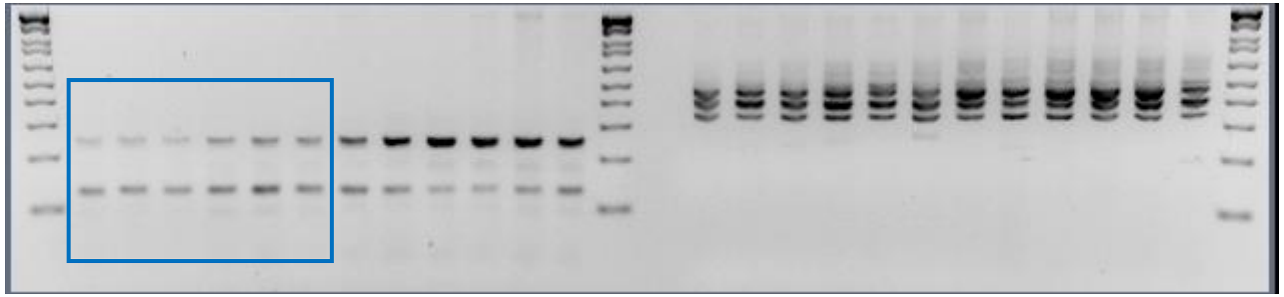

Figure 2G

Dffb

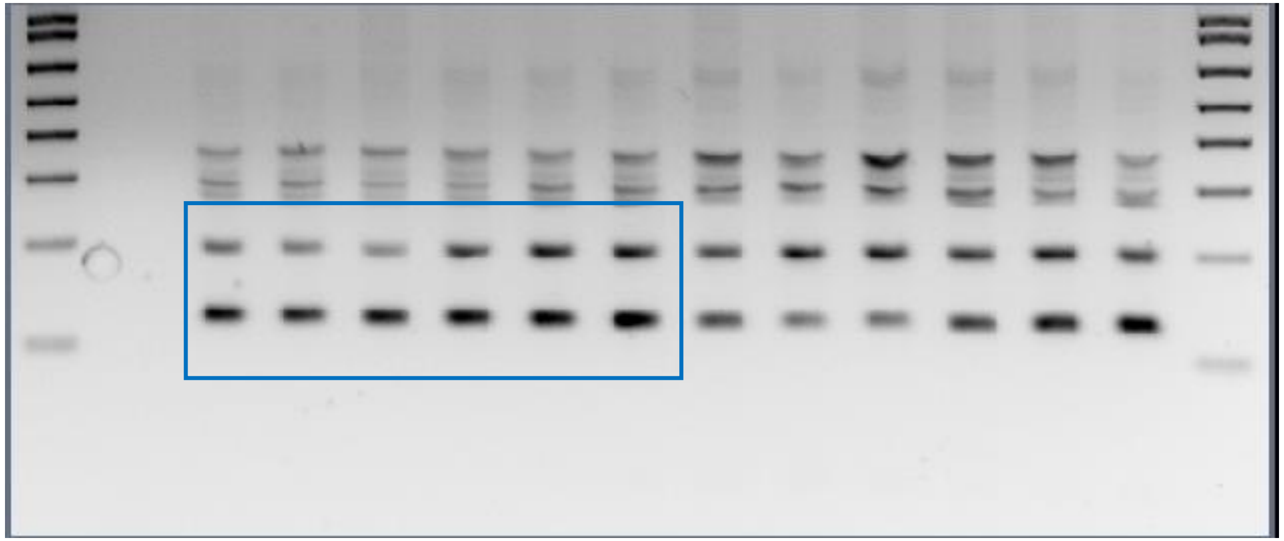

Figure 2G

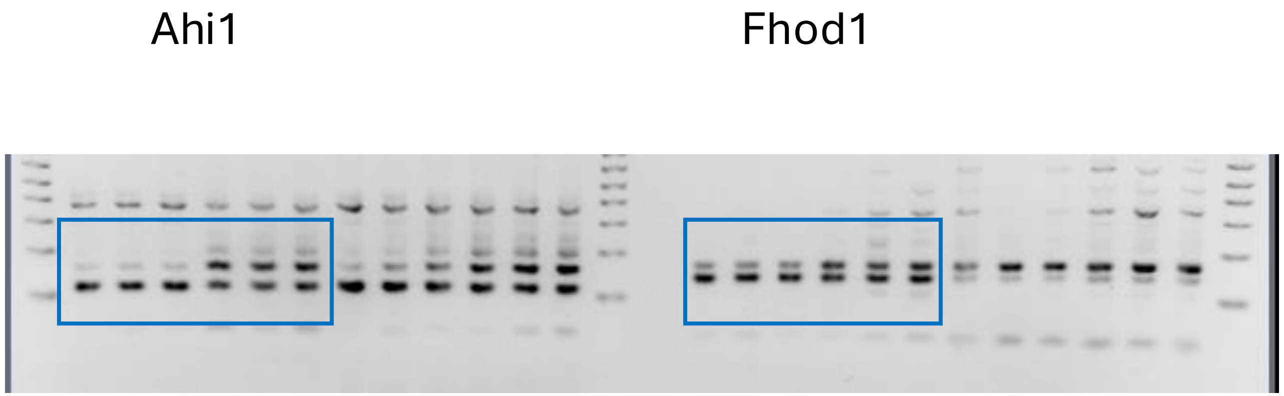

Figure 2G

Akap8

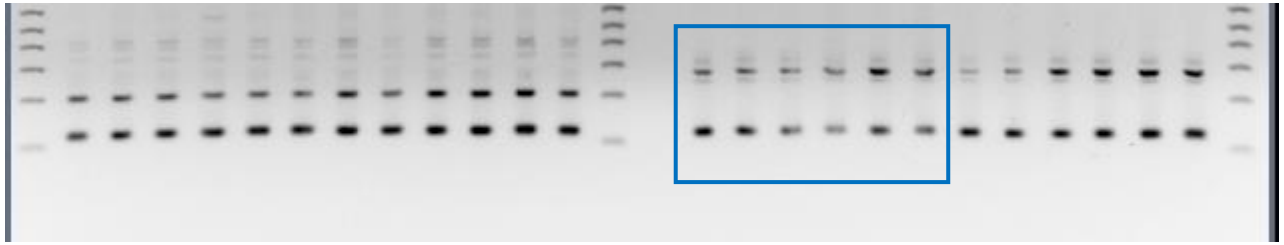

Figure 2G

Lpcat2

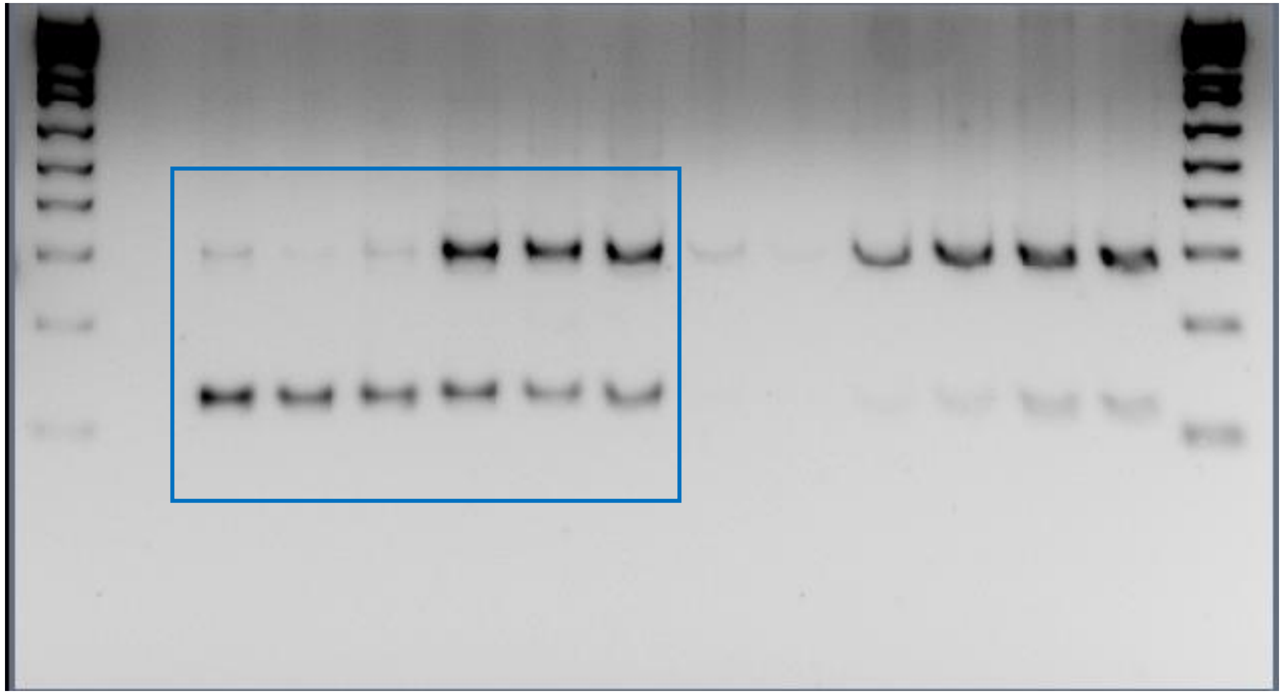

Figure S5G

Gria2

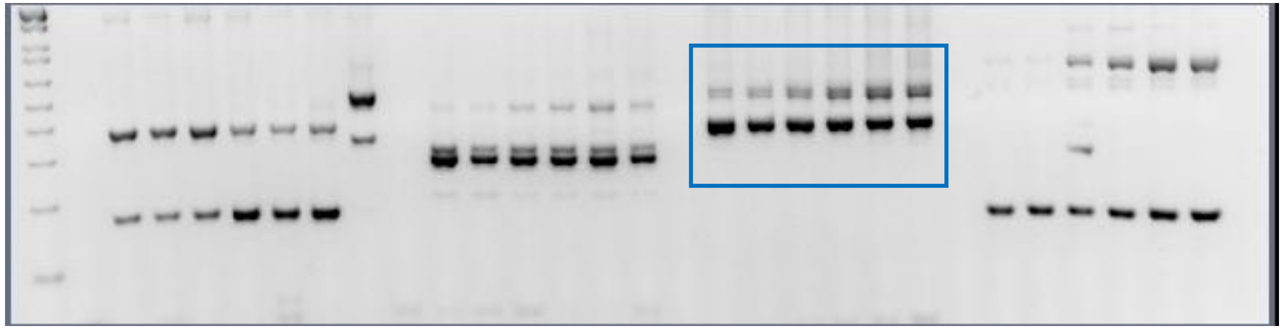

Figure S5G

Rock1

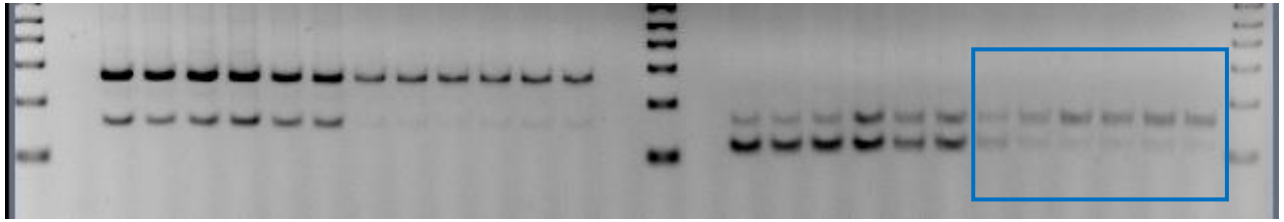

Figure S5G

Ctnnal1

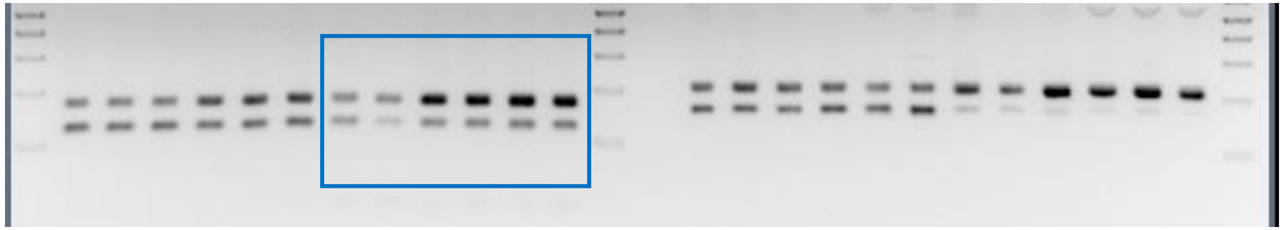

Figure S5G

Hps5

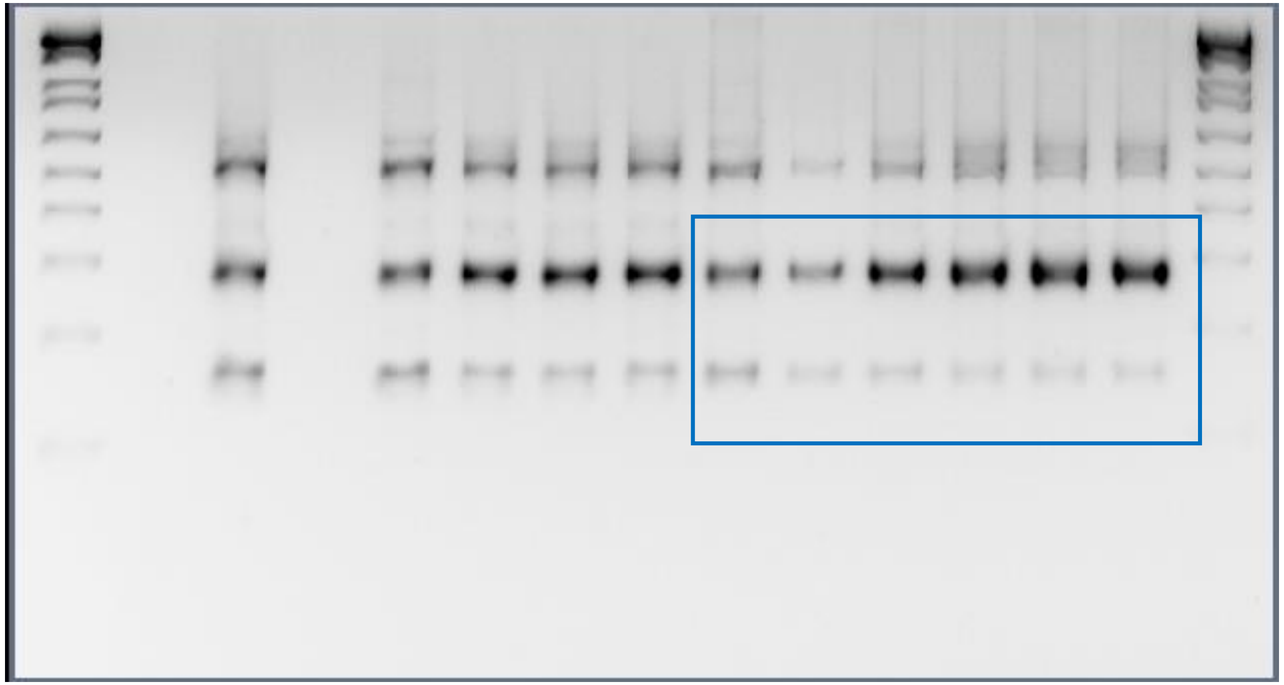

Figure S5G

Gad1

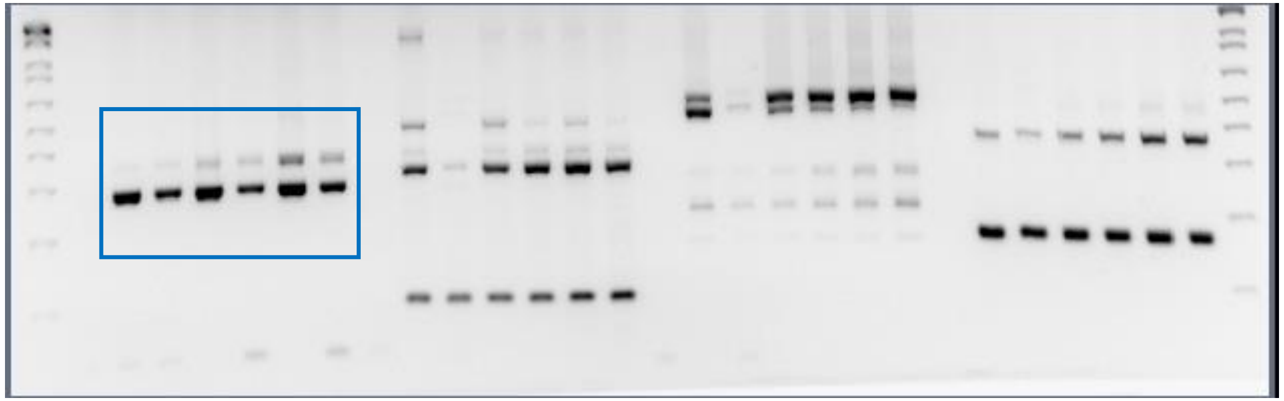

Figure S5G

Stx3

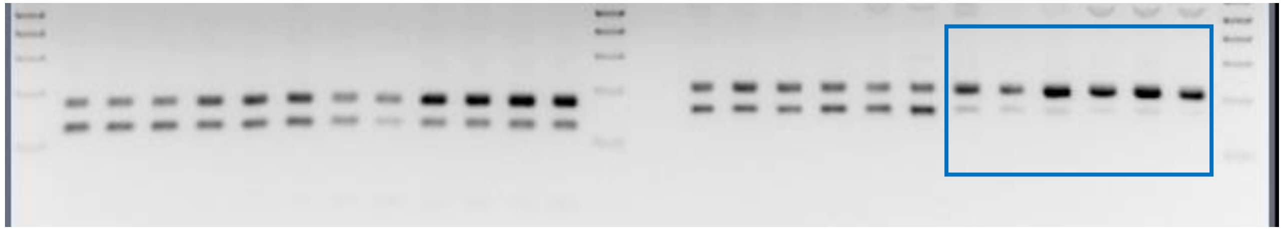

Figure S5G

Ripk2

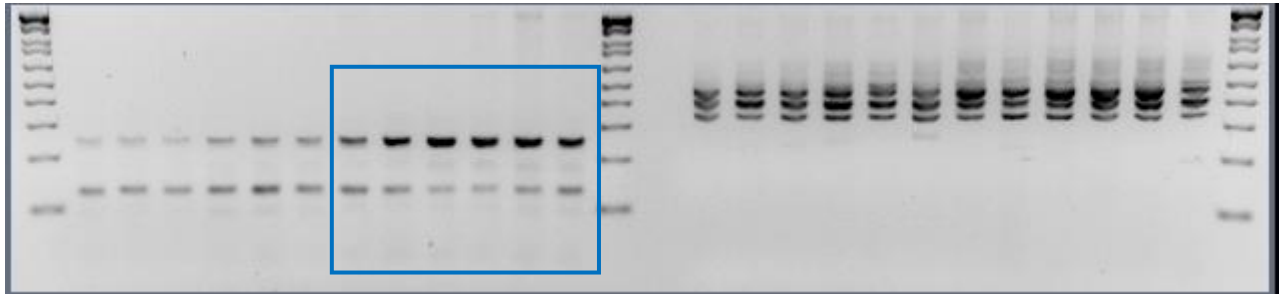

Figure S5G

Akap8

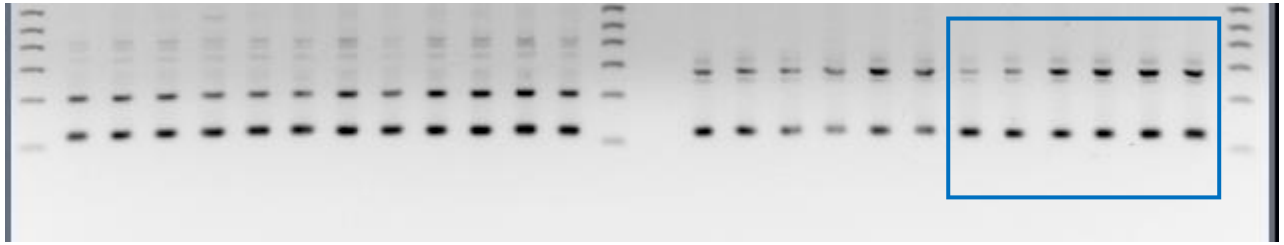

Figure S5G

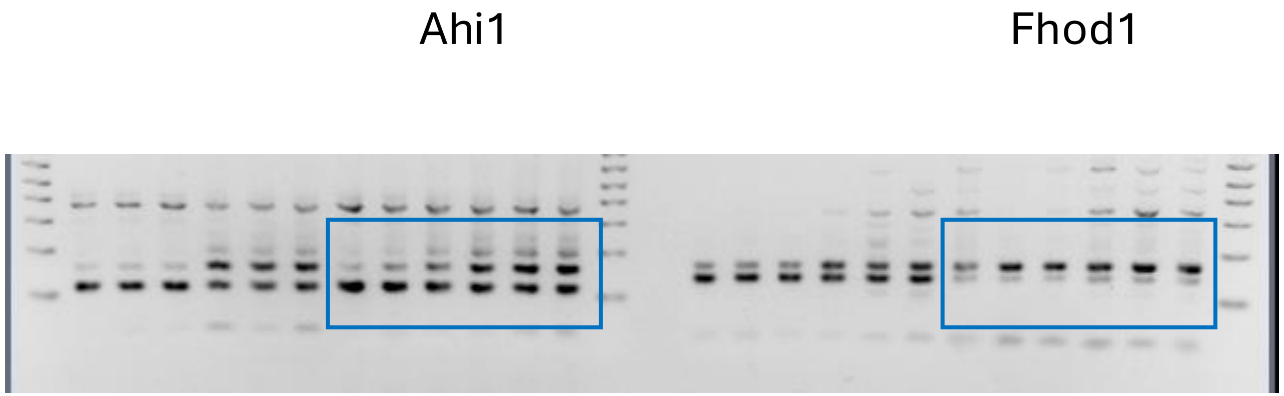

Figure S5G

Invs

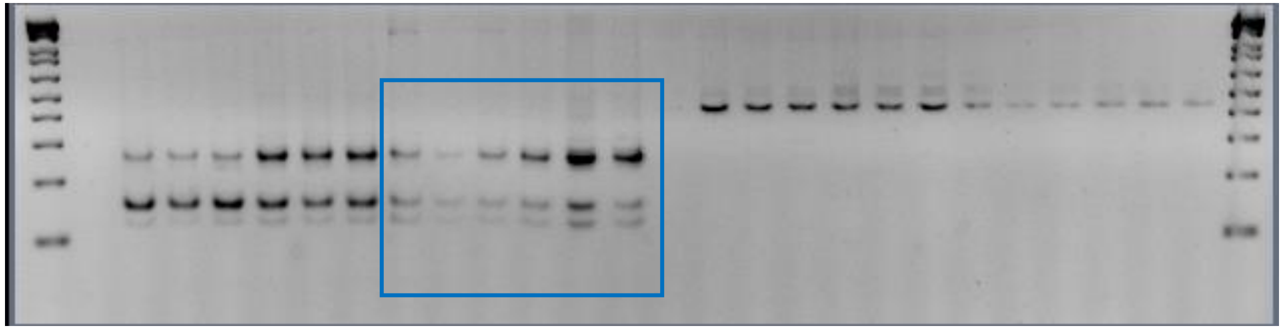

Figure S5G

Lpcat2

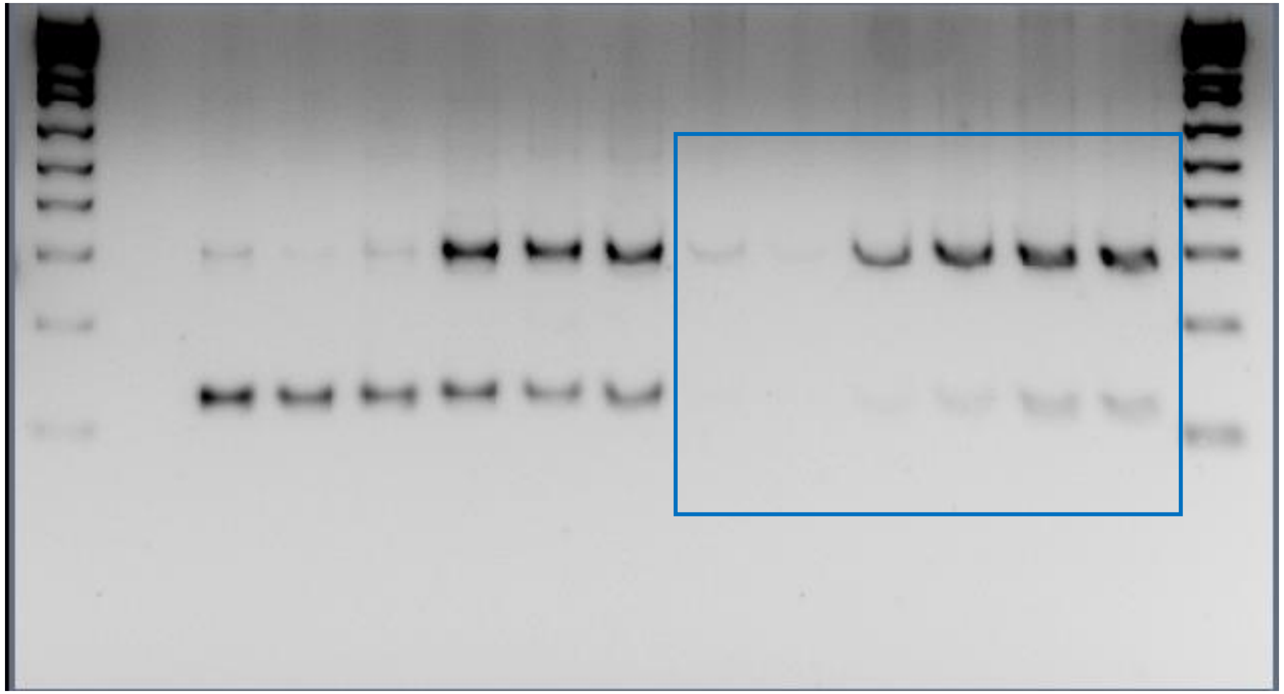

Figure S6A

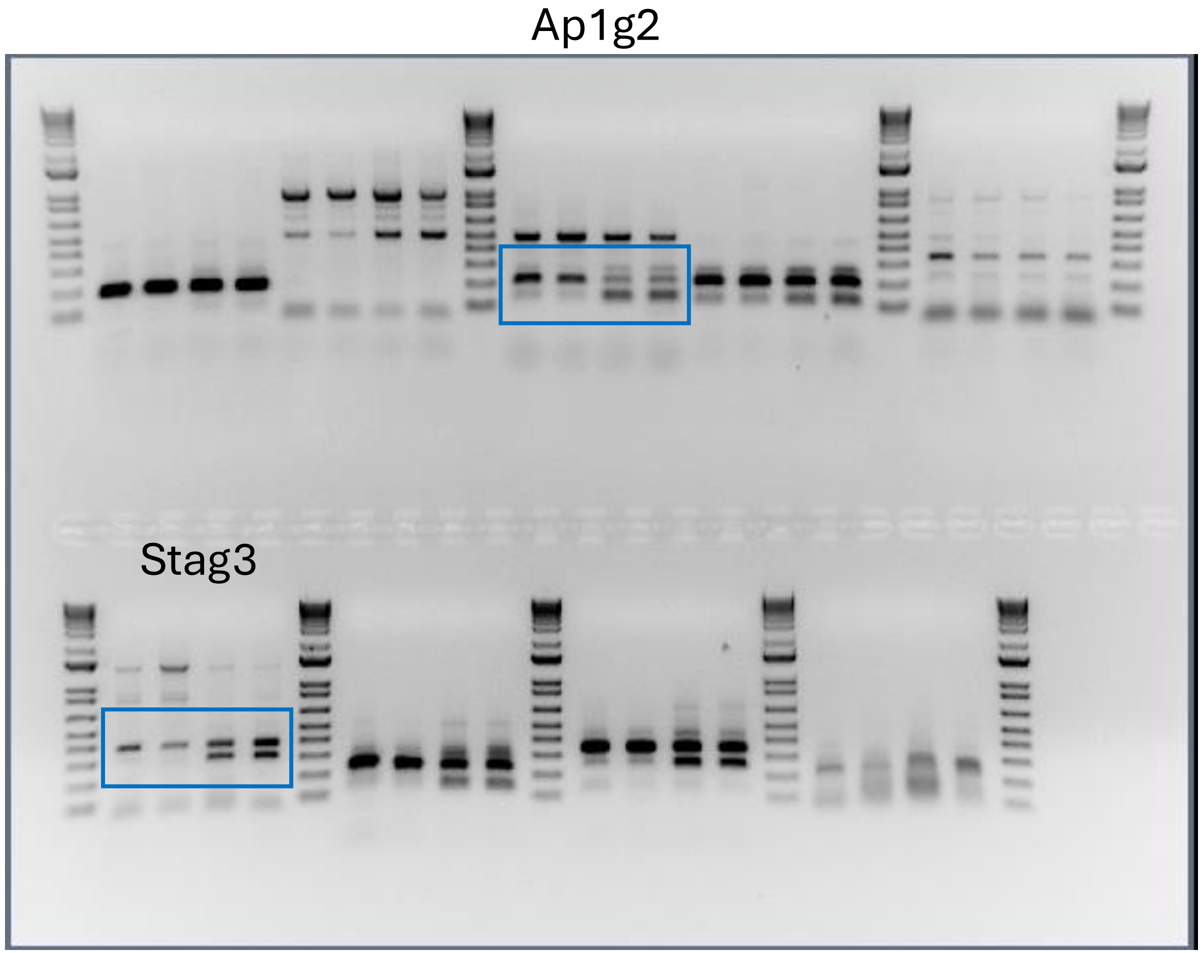

Figure S6A

Stxbp2

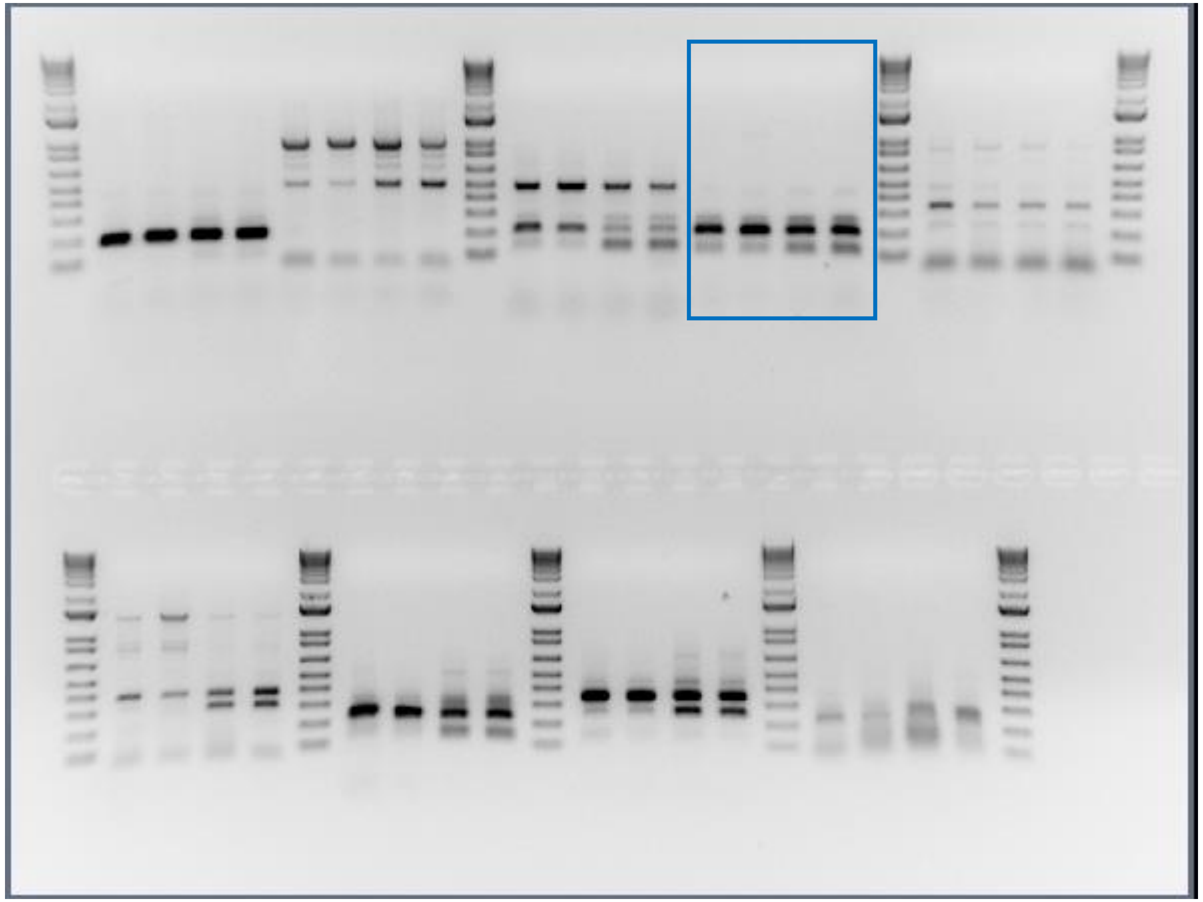

Figure S6A

Cacna1b

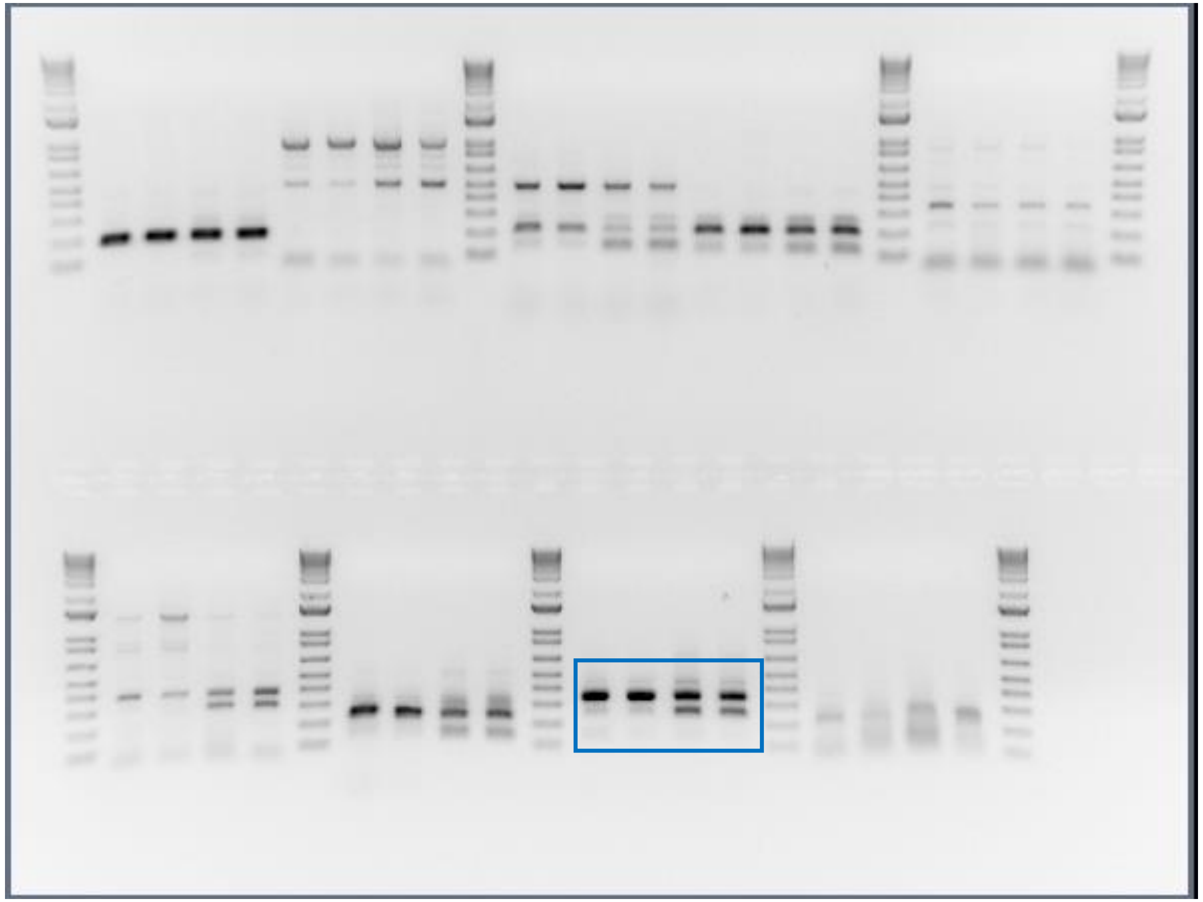

Figure S6A

Gabbr1

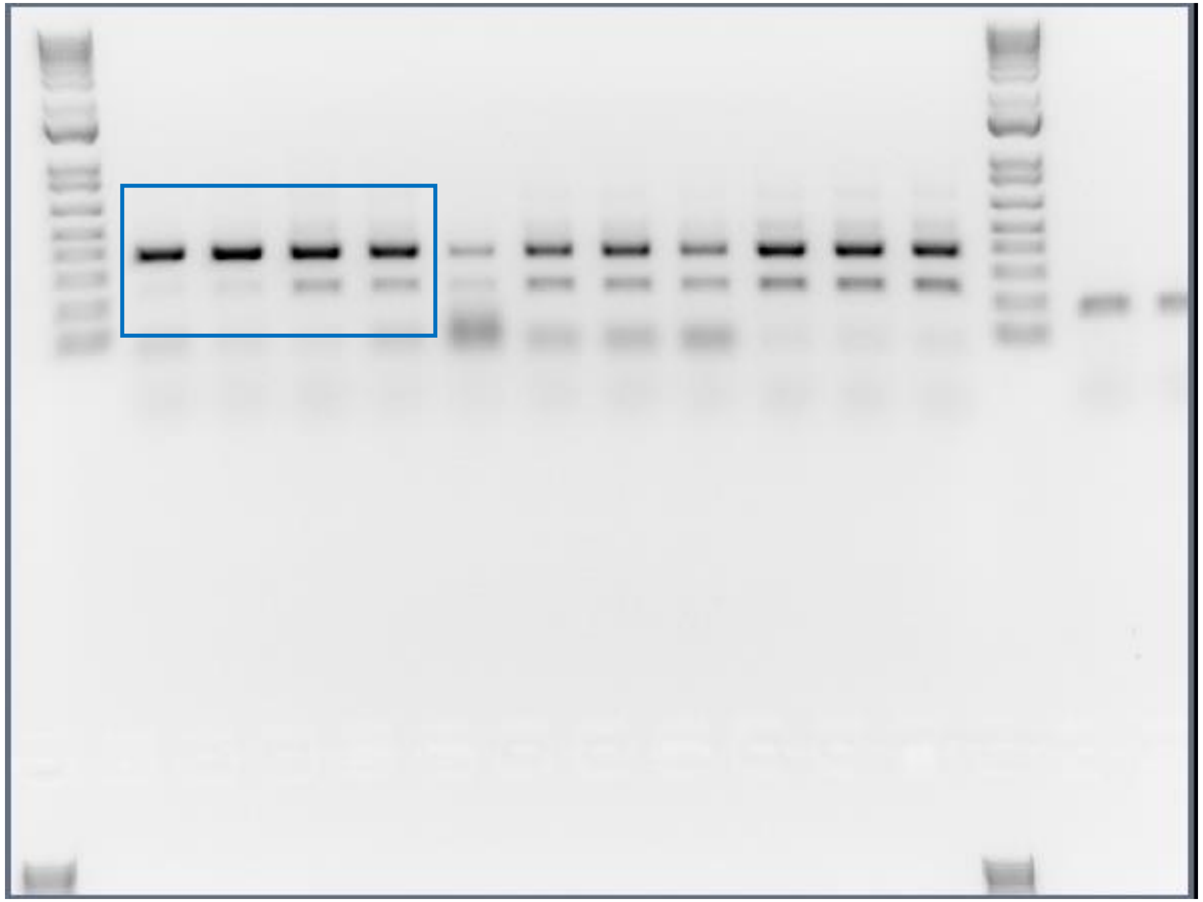

Figure 6D

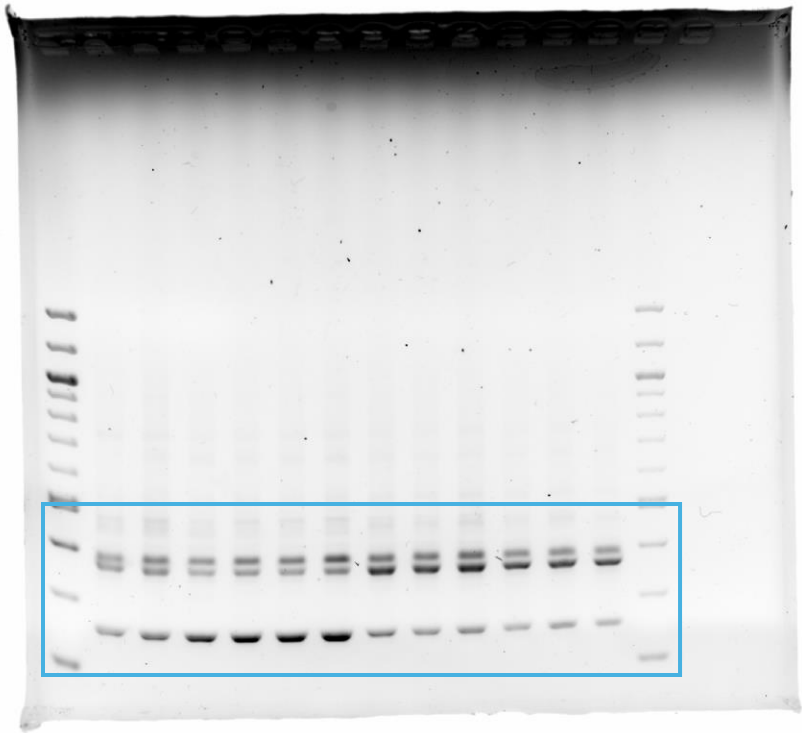

Figure S13E

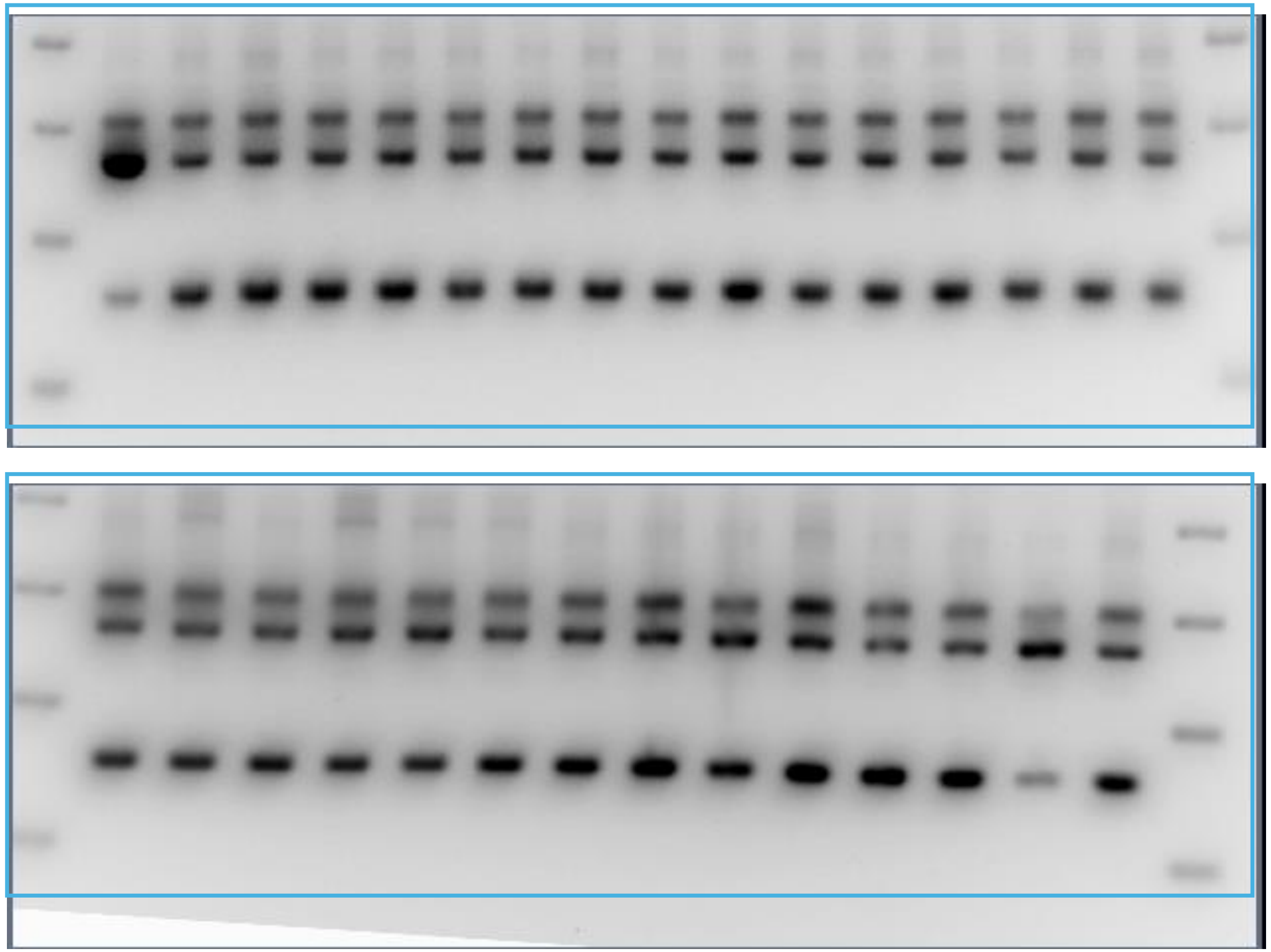

Figure S13F

ASO 1137 AND 1150 in SH-SY5Y

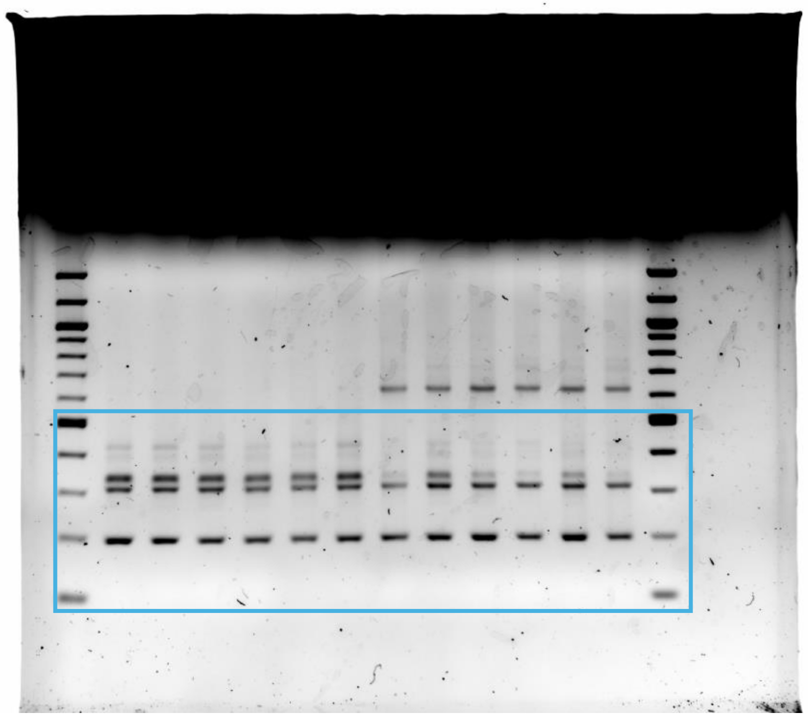

Figure S13G

ASO1137 dose in HEK293T

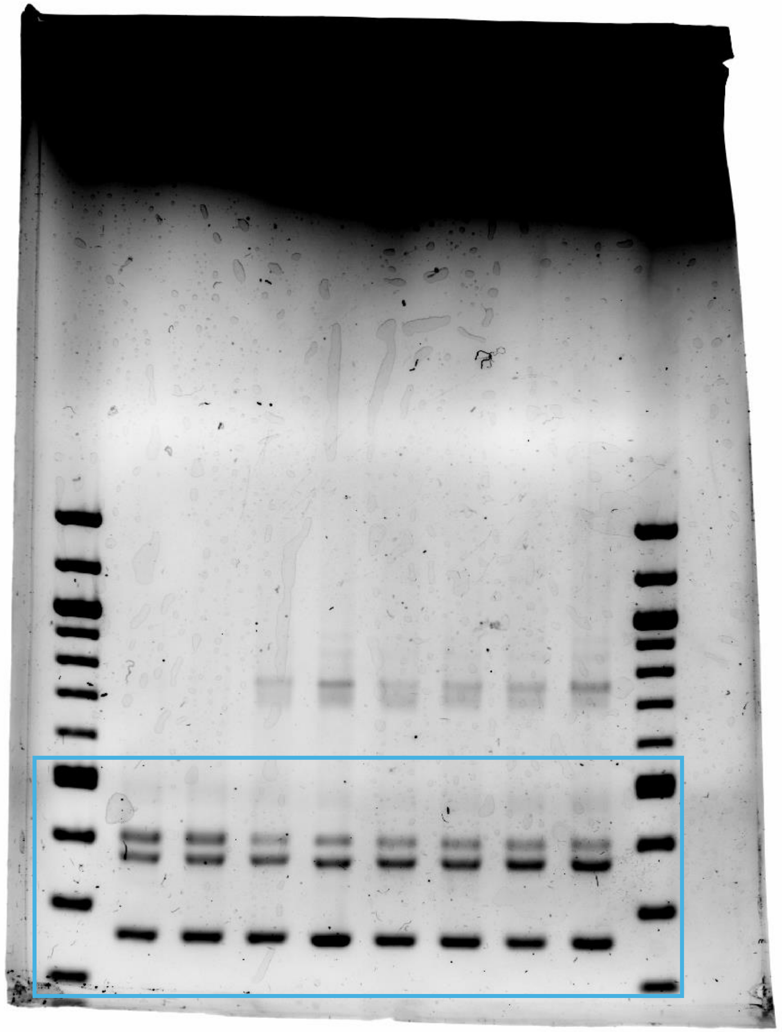

Figure S13H

ASO1137 dose in SH-SY5Y

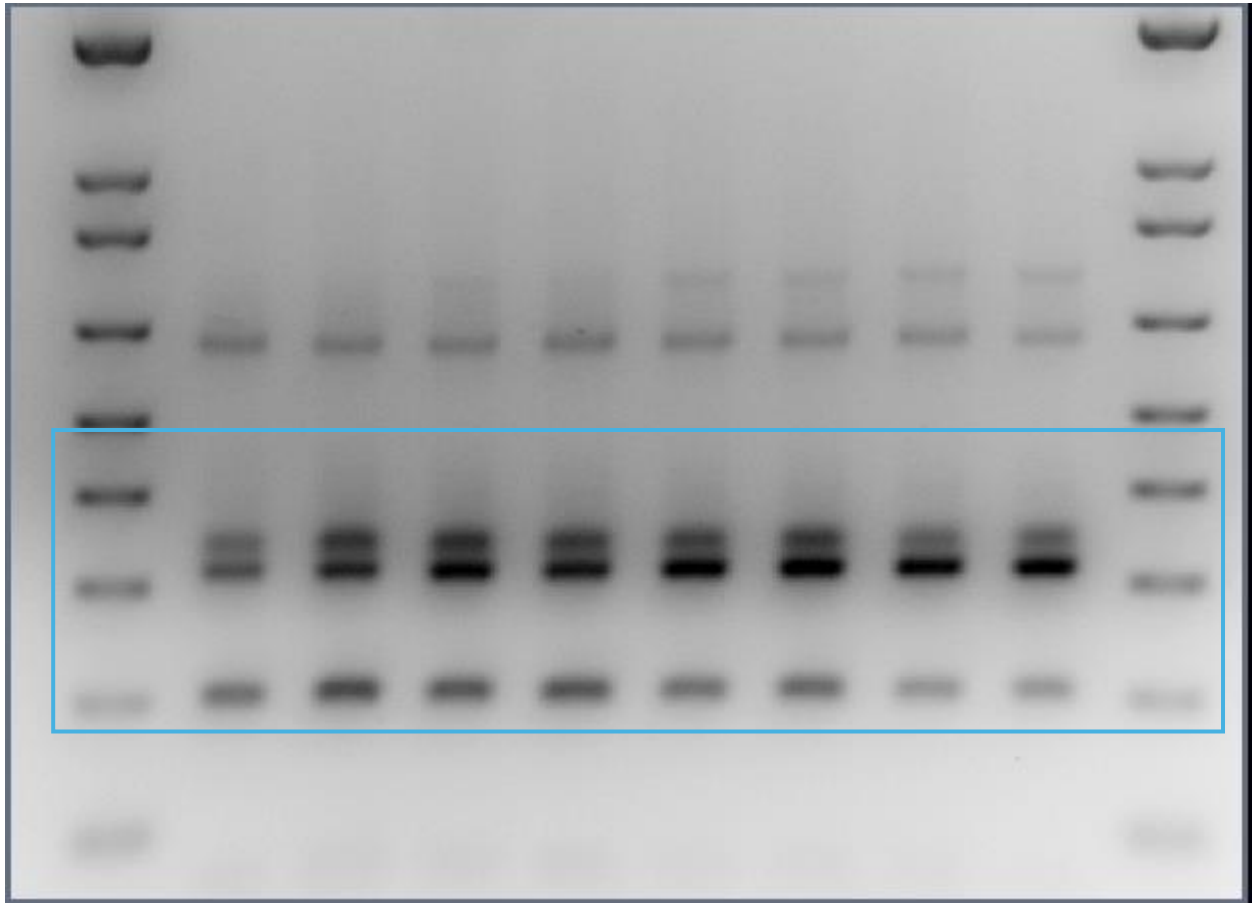

Figure S13I

ASO1150 dose in HEI

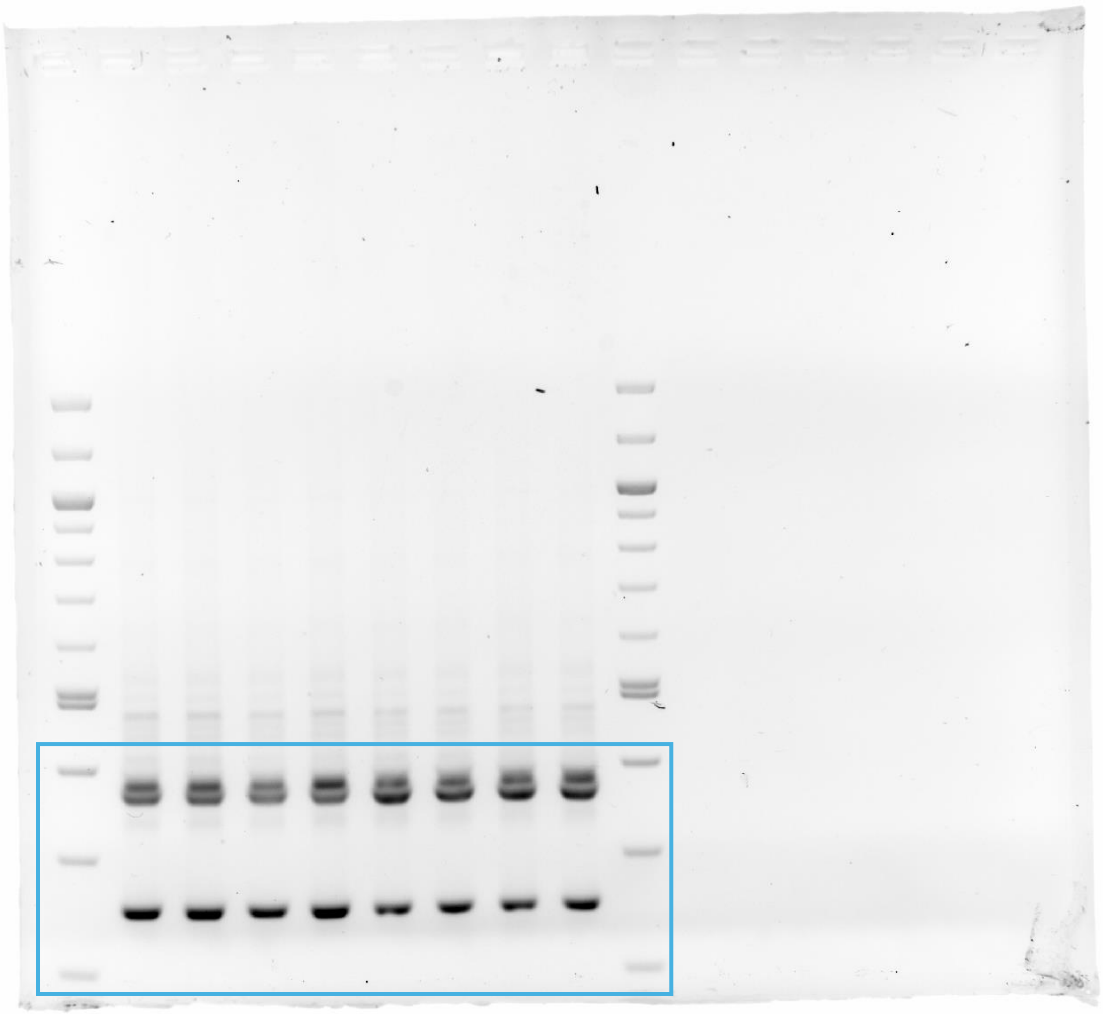

Figure S13J

ASO1150 dose in SH-SY5Y

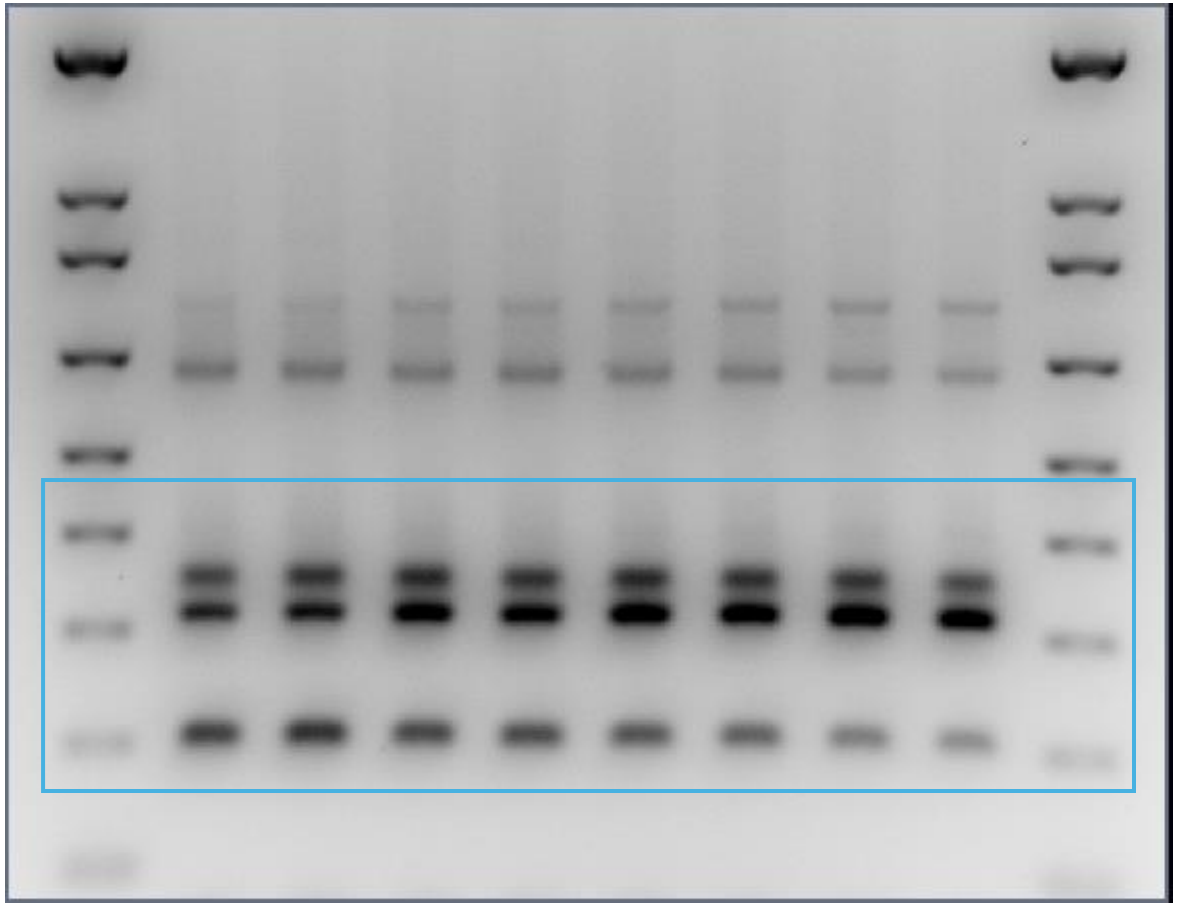

Supplement: Unedited blot and gel images [file jci-136-197271-s059.pdf]
